# Structure and receptor recognition of type-II feline infectious peritonitis virus spike glycoprotein

**DOI:** 10.1038/s44318-026-00849-2

**Published:** 2026-07-08

**Authors:** Sheng Niu, Yuyang Tian, Linh Nguyen, Chongzhi Bai, Xiaohan Hou, Dan Wang, Ziqian Niu, Zhimin Liu, Jianle Ren, Wentao Li, Qihui Wang, Wen-xia Tian, Junqing Sun, George Fu Gao

**Affiliations:** 1 Shanxi Key Laboratory of Animal Disease Research, Prevention and Control, College of Veterinary Medicine, Shanxi Agricultural University, Jinzhong, China; 2 CAS Key Laboratory of Pathogen Microbiology and Immunology, Institute of Microbiology, Chinese Academy of Sciences (CAS), Beijing, China; 3 Central Laboratory, Shanxi Province Hospital of Traditional Chinese Medicine, Taiyuan, China; 4 National Key Laboratory of Agricultural Microbiology, Hubei Hongshan Laboratory, College of Veterinary Medicine, Huazhong Agricultural University, Wuhan, China; 5 Innovative Vaccine and Immunotherapy Research Center, the Second Affiliated Hospital, Zhejiang University School of Medicine, Hangzhou, China; 6 Transvascular Implantation Devices Research Institute, Hangzhou, China

**Keywords:** Microbiology, Virology & Host Pathogen Interaction, Structural Biology

## Abstract

Type-II feline infectious peritonitis virus (FIPV-II) is a lethal alphacoronavirus, whose high homology with the human coronavirus CCoV-HuPn-2018 carries the risk of potential zoonotic FIPV-II transmission to humans. FIPV-II infects felids using cat aminopeptidase N (cAPN) as its cell-entry receptor, but the molecular details remain unclear. Here, we resolved cryo-electron microscopy (cryo-EM) structures of the spike (S) trimer of a representative FIPV-II strain 79-1146 (FIPV-1146), and of its complex with cAPN. The reconstructions reveal structural transitions of the S protein between the “standing” and “lying” receptor-binding domain (RBD) conformation upon complex formation with cAPN in its open conformation. Structural and mutational analyses revealed that the cAPN residues R378, D751 and R779, as well as the N748-linked glycan are essential for high-affinity RBD engagement. Cross-species analysis demonstrated narrow tropism of FIPV-1146, limited to felids and canids. Introducing the N595K and Q596R substitutions found in transmissible gastroenteritis virus (TGEV) into the FIPV-1146 RBD expands its binding capacity to APNs from pig, bovine, horse and giant panda. These findings provide insights into the entry mechanism and zoonotic constraints of FIPV-II, with potential relevance for antiviral strategies against coronaviruses.

## Synopsis

Type-II feline infectious peritonitis virus (FIPV-II) is a lethal alphacoronavirus with highly homologous to human coronavirus CCoV-HuPn-2018. Here, cryo-EM structures of the FIPV-II spike (S) protein with its receptor cAPN identify key residues and glycan modifications essential for receptor binding, as well as mutations expanding its tropism beyond felids.

- Interaction of the FIPV-1146 S protein receptor-binding domains (RBDs) with cAPN induces a conformational change in spike protein.
- Mass spectrometry and cryo-EM together reveal the N- and O-glycosylation landscape of the FIPV-1146 S protein.
- High-affinity binding between FIPV-1146 RBD and cat APN primarily depends on cAPN residues R378, D751, R779, and the N748-linked glycan.
- FIPV-1146 only infects felids and canids, but N595K/Q596R mutations enable binding to four additional mammalian APNs.

## Abstract (web summary)

Structural and interaction analyses of the FIPV-II spike protein with its receptor elucidate its cell recognition mechanism and zoonotic constraints.

## Graphical abstract

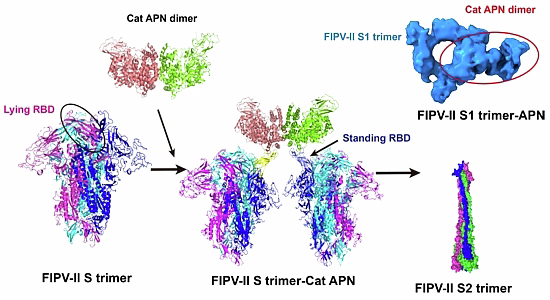

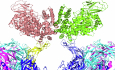

## Introduction

Zoonotic transmission of viruses from nonhuman animals poses significant threats to global public health and economic development (Gao, 2018; Liu et al, 2025; Su et al, 2016). Coronaviruses (CoVs) cause a wide range of diseases in both humans and animals, affecting respiratory, gastrointestinal, neurological, and other tissue tropisms, resulting in increasing attention and research priority (Lednicky et al, 2021; Liu et al, 2023b; Vlasova et al, 2022a; Zhu et al, 2020). In recent years, the detection of human-origin porcine deltacoronavirus (Hu-PDCoV) and canine CoV (Hu-CCoV) has highlighted the risk of cross-species transmission of these zoonotic CoVs (Lednicky et al, 2022; Lednicky et al, 2021; Vlasova et al, 2022a; Vlasova et al, 2022b). Felids are no exception to this trend, and the feline coronavirus (FCoV) poses a significant health threat to both wild and domestic species (Kipar and Meli, 2014). FCoV, belonging to the α-CoV genus, is commonly proposed to exist in two biotypes. Feline enteric coronavirus (FECV) primarily causes subclinical disease, whereas feline infectious peritonitis virus (FIPV) manifests as an aggressive and severe form (Jaimes et al, 2020). Feline infectious peritonitis (FIP) is one of the most fatal infectious diseases in cats, with a mortality rate reaching 100% (Balint et al, 2014; Pedersen, 2014). Currently, there are no effective vaccines available. In 2023, a newly identified FCoV-II strain (named FCoV-23) was responsible for a large FIP outbreak in cats on the Mediterranean island of Cyprus. Furthermore, this virus was documented as having spread to the UK through an import-related case (Warr et al, 2023).

Receptor binding is a prerequisite for viral transmission and infection. CoVs rely on their spike (S) proteins to recognize host cells and mediate membrane fusion, enabling viral genome entry and replication (de Wit et al, 2016; Li et al, 2022; Tan et al, 2023; Wang and Wang, 2023). Thus, blocking the interaction between S protein and host receptors is the primary antiviral strategy (Qu et al, 2023; Wu et al, 2025). Extensive researches have revealed that numerous CoVs, including severe acute respiratory syndrome coronavirus 2 (SARS-CoV-2), PDCoV, and transmissible gastroenteritis coronavirus (TGEV), can infect a wide range of hosts as demonstrated by the cross-species binding capacity of their S protein receptor-binding domains (RBDs) to heterologous receptors (Niu et al, 2021; Tian et al, 2025; Wu et al, 2020). Based on the significant antigenic differences among FIPV, they have been categorized into two distinct serotypes: type I and type II (Hohdatsu et al, 1991; Pedersen et al, 1984). The primary receptor for FIPV-I remains to be identified (Pedersen, 2014). Aminopeptidase N (APN), a common receptor for human coronavirus 229E (HCoV-229E), TGEV, and PDCoV, has been identified as a key entry receptor for FIPV-II (Tian et al, 2025; Tsai et al, 2025; Tusell et al, 2007). However, the molecular basis of FIPV-II S binding to cat APN (cAPN) remains elusive, limiting further research on the molecular mechanisms of FIPV-II invasion.

CoVs often break through the host barrier and spread among animals, sometimes during large epidemics (Su et al, 2016; Zhu et al, 2020). Epidemiological data indicate that FIPV can infect not only household and wild cats, but also other species, including African lions, cheetahs, and ferrets (Martinez et al, 2008; Pedersen, 2009). Viruses genetically similar to canine and feline CoVs have been identified in respiratory samples of patients in Haiti and Malaysia (Lednicky et al, 2022; Vlasova et al, 2022a). Therefore, the risk of cross-species FIPV transmission should be rigorously assessed.

To better understand the cellular invasion mechanism of FIPV-II, we solved the cryo-electron microscopy (cryo-EM) structure of a representative strain FIPV 79-1146 (FIPV-1146) S protein at 2.78 Å resolution, along with two lower-resolution structures of the FIPV-1146 S trimer in complex with cAPN. Additionally, we resolved the cryo-EM structure of FIPV-1146 RBD bound to cAPN at 2.85 Å resolution. We further systematically analyzed the interactions between FIPV-1146 RBD and APN orthologs from 17 different species, including domestic animals, companion pets, and some wild animals. These structural and functional findings revealed the receptor specificity of FIPV-II and provided insights into its viral entry mechanisms and pathogenesis.

## Results

### Cryo-EM structure of the FIPV-1146 S trimer

**Figure 1:**
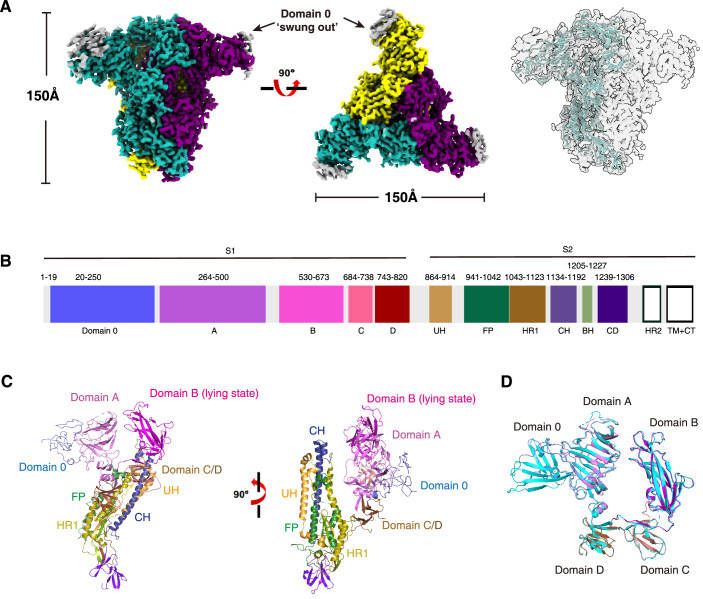
Cryo-EM structure of FIPV-1146 S protein. (**A**) The EM density maps of FIPV-1146 S ectodomain with atomic model fitted in. The maps have a contour of 7.0 σ. (**B**) Functional subunits and domains are indicated and colored as defined in the schematic representation as a function of the sequence number indicated above each functional unit. CD connector domain, CH central helix, CT cytoplasmic tail, FP fusion peptide, TM transmembrane domain, UH upstream helix. The black line boxes denote the regions that are unresolved in the density map (HR2) or not part of the construct (TM and CT). (**C**) Cartoon representative of the atomic model of monomeric FIPV-1146 S protein. The domains are indicated and colored as defined in (**B**). (**D**) Superimposition of FCoV-23 (cyan) and FIPV-1146 (colored as defined in (**B**)) S1 subunits with D0 swung out.

The cryo-EM structure of the prefusion-stabilized S glycoprotein ectodomain trimer from FIPV-1146 at 2.78 Å resolution was solved (Appendix Figs. S1 and S2; Appendix Table S1). The reconstructed 3D map revealed a propeller-shaped homotrimer with approximate dimensions of 150 × 150 Å (diameter × height) (Fig. 1A), similar to all the known CoVs structures, consisting of an N-terminal S1 subunit containing five distinct domains (0:20–250; A: 264–500; B: 530–673; C: 684–738; D: 743–820) and a C-terminal S2 subunit responsible for membrane fusion (Fig. 1B,C). As a representative FIPV-II, FIPV-1146 S shares high sequence homology (93.32%) with that of the recently reported FCoV-23, although the similarity is relatively lower in Domain 0 (83.84%) (Appendix Figs. S3 and S4). Structurally, the S protein of FIPV-1146 exhibits a conformation where all three Domain 0 regions are in the “swung out” position, a state highly similar to one of the conformations observed in FCoV-23 (S1 subunit RMSD = 0.5 Å) (Fig. 1D; Appendix Fig. S4A). The structural similarity to the S1 subunit of CCoV-HuPn-2018 is slightly lower (RMSD = 0.6 Å) (Appendix Fig. S4B). Notably, in contrast to FCoV-23, 3D classification did not identify the FIPV-1146 trimers with a Domain 0 in the proximal conformation (Appendix Fig. S1). Although FIPV-I (FIPV-UU4; Uniprot: C6GHB7) and FIPV-II S (FIPV-1146, Uniprot: P10033) proteins share only 45.9% amino acid sequence identity, they maintain a similar overall structural architecture (Appendix Fig. S4A). The S1 subunit in both types adopts a square-shaped conformation (Appendix Fig. S4B). This quaternary structure is characteristic of alpha-CoVs and delta-CoVs, setting them apart from the distinct V-shaped organization observed in the S glycoproteins of beta-CoVs and gamma-CoVs (Shang et al, 2018; Yuan et al, 2017). The most pronounced structural divergence occurred in domain 0, which adopted entirely distinct conformations between the two serotypes (Appendix Fig. S4B).

In the FIPV-1146 S trimer, the RBD consistently reside in the “lying” state, mirroring the observations in both FCoV-23 and CCoV-HuPn-2018 (Fig. 1C; Appendix Fig. S4A). Notably, a subpopulation of disassociated S1 trimers was identified during 2D classification, leading to the determination of its cryo-EM structure at 4.89 Å resolution (Appendix Fig. S1G,H). The S1 trimer forms a ring-like architecture with a markedly larger diameter than the intact S trimer (Appendix Fig. S5A,B), and could not be fitted by the S trimer atomic model (PDB: 9UE3). Domain-level fitting revealed that all three RBDs in this dissociated state adopt a “standing” conformation (Appendix Fig. S5A,C). Structural analysis further demonstrated that, unlike the sterically hindered intact trimer, the disassociated S1 trimer readily accommodates cAPN binding without clashes (Appendix Fig. S5C,D). While these findings provide a plausible structural basis for receptor accessibility, the disassociated S1 trimer may represent an experimental artifact, and its biological significance requires further validation.

**Figure 2:**
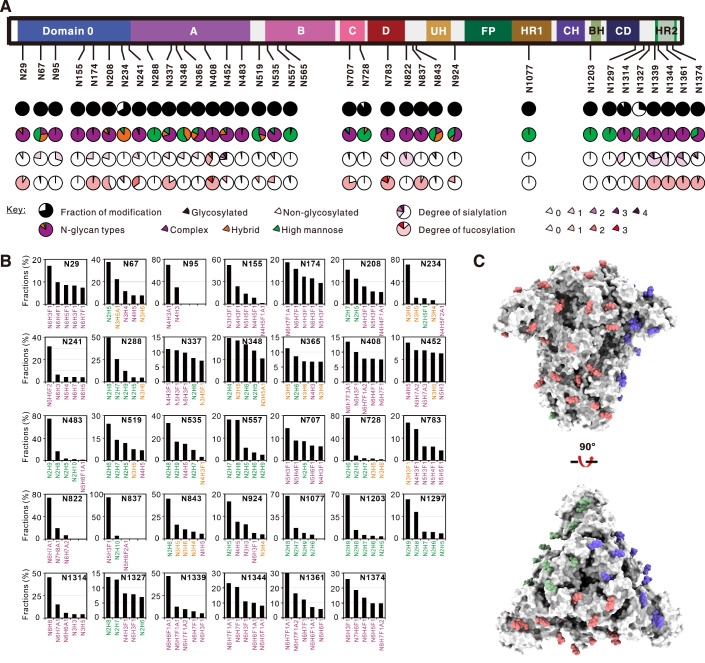
Site-specific N-glycosylation analysis of FIPV-1146 S. (**A**) Distribution of N-linked glycosylation sites on the one-dimensional structure of protein with respective composition of N-glycans types and degrees of sialylation and fucosylation. (**B**) Fractional abundances of five most abundant N-glycan structures at each N-site. Key: purple, orange and green indicate complex, hybrid and high mannose structures, respectively. Detailed profiling of N565 glycosylation were not shown as only deamidated peptide was detected. (**C**) Structural mapping of the glycosylation of FIPV-1146 S using the PDB entry 9UE3 as the structural template. The proteinaceous parts are shown in white surfaces and the N-glycans are colored by chain.

N-linked glycosylation contributes to protein folding, stability, and viral tropism (Jia et al, 2022; Liu et al, 2023a). FIPV-1146 S harbor 35 putative N-glycosylation sites (N-sites). By utilizing mass spectrometry (MS)-based site-specific glycosylation profiling combined with cryo-EM, we quantitatively determined the glycosylation profile and assigned the predominant glycoforms at each N-site (Appendix Table S2). All 35 putative N-sites on FIPV-1146 S were quantitatively profiled by MS, among which only N565 were detected as deamidated but not glycosylated peptides (Fig. 2A,B). Most of N-sites are fully glycosylated while N234 (on domain 0) and N1327 were 67% and 28% glycosylated. Regarding glycan types, N288, N348, N519, N557, N728, N1077, N1203 and N1297 predominantly contain high-mannose N-glycans, with mostly HexNAc(2)Man(5-9). Other N-sites contained multiple structures with mostly hybrid HexNAc(3)Hex(6) on N234 and complex N-glycans on the remaining. Most N-glycan structures detected were non- or mono-sialylated and fucosylated. Among these N-sites, 23 exhibited prominent protruding EM density to confirm the N-glycosylation (Fig. 2C), namely N234, N241, N288, N337, N348, N365, N408, N452, N483, N519, N535, N565, N557, N707, N728, N783, N822, N837, N843, N924, N1077, N1203 and N1297, which were verified by both MS and cryo-EM (Appendix Table S2).

In our study, we also detected multiple O-sites including 12 sites having “O-Follow-N” pattern (in proximity to N-sites) and 7 other sites, which were confirmed by at least 3 different treatments (Appendix Table S3). However, the percentage of modification on these O-sites were estimated to be less than 10%.

### Overall structure of FIPV-1146 S in complex with cAPN

**Figure 3:**
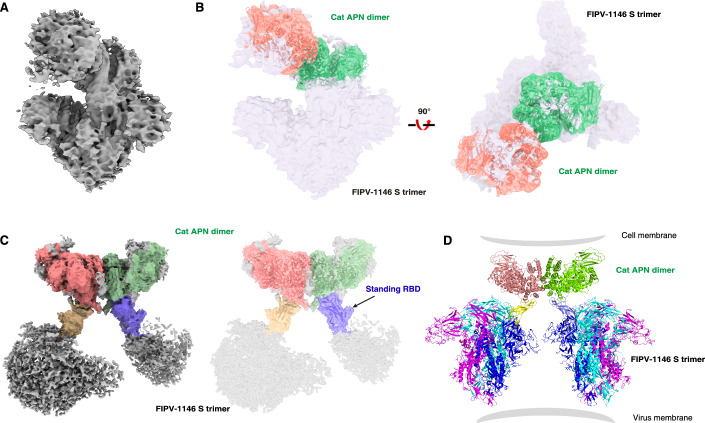
Low resolution cryo-EM structures of FIPV-1146 S trimer bound to cAPN. (**A**) The EM density of the overall structure of an FIPV-1146 S trimer bound to a cAPN dimer. (**B**) The cartoon representation of cAPN dimer is colored by chain overlaid with the light-gray silhouette of the cryo-EM map. (**C**) The EM density of the overall structure of two FIPV-1146 S trimer bound to a cAPN dimer. The cartoon representation of the structures of both FIPV-1146 RBD and cAPN dimer are colored by chain overlaid with the light-gray silhouette of the cryo-EM map. (**D**) A proposed model showing the binding of two FIPV-1146 S trimers to one cAPN dimer. The atomic model was produced by generating a structure with AlphaFold2 (template: 8WDE), fitting and refining it in Coot against the cryo-EM maps (**A**, **C**), and finally visualizing it in PyMOL. Source data are available online for this figure.

Similar to FCoV-23 and CCoV-HuPn-2018, the RBD of free FIPV-1146 S adopts a “lying” conformation, which necessitates the conformational change to expose key binding loops for APN interaction (Tortorici et al, 2025; Tortorici et al, 2022). To capture this interaction, we incubated the S protein with cAPN and prepared cryo-EM samples, which unexpectedly yielded a 5.09 Å reconstruction of the FIPV-1146 S–cAPN complex (Fig. 3A,B; Appendix Fig. S6). While this map confirmed that one cAPN dimer binds one S trimer (Fig. 3A,B), the limited resolution precluded unambiguous assignment of the binding domain, though RBD was strongly implicated. To verify this, we adopted a strategy similar to that used in the recently published HCoV-229E S-human APN (hAPN) complex, involving purification of the S–cAPN complex prior to grid preparation (Appendix Fig. S7A) (Tsai et al, 2025). 3D classification yielded a 3.53 Å structure revealing one cAPN dimer bound to two RBD of FIPV-1146 S (Fig. 3C; Appendix Fig. S7F). However, the full S region remained unresolved, likely due to conformational heterogeneity and instability. Based on these findings, we propose a model in which the RBD transitions to a “standing” conformation to enable cAPN binding, as the “lying” conformation would cause steric clashes (Fig. 3D). Additionally, during 3D classification, we identified a distinct FIPV-1146 S conformation featuring a mixed D0 conformation (Appendix Fig. S7E), suggesting that FIPV 1146 may also adopt the alternative D0 conformations observed in FCoV-23.

### The complex structure of cAPN bound to the FIPV-1146 RBD

**Figure 4:**
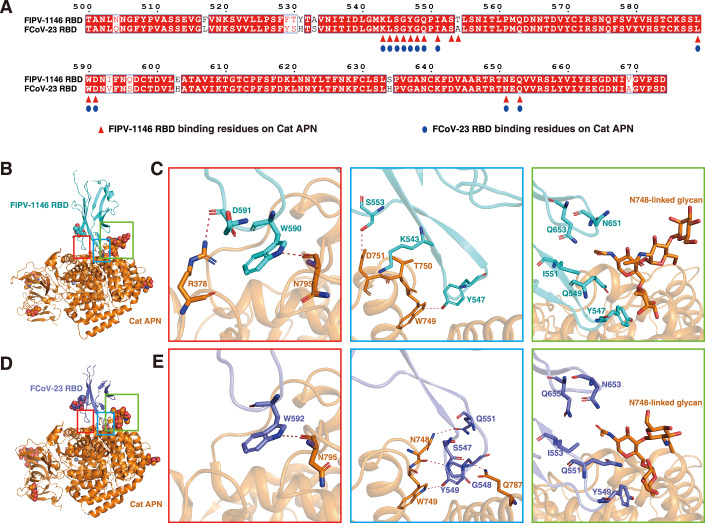
The complex structures of FIPV-1146 RBD and FCoV-23 RBD bound to cAPN. (**A**) Sequence alignment of the RBD sequences from the FIPV-1146 and FCoV-23. Red triangles/ blue circles indicate the FIPV-1146/FCoV-23 RBD binding residues on cAPN. Identical residues are highlighted in red background. (**B**) The overall complex structure of cAPN bound to the FIPV-1146 RBD. The binding between the FIPV-1146 RBD and cAPN is mainly composed of three patches of interactions, and patch 1, patch 2 and patch 3 are indicated in red, blue and green boxes, respectively. (**C**) Detailed interaction of cAPN with the FIPV-1146 RBD in the three patches. (**D**) The overall complex structure of cAPN bound to the FCoV-23 RBD. (**E**) Detailed interaction of cAPN with the FCoV-23 RBD in the three patches. H-bonds were identified using PDBePISA (https://www.ebi.ac.uk/msd-srv/prot_int/pistart.html). Residues involved in the H-bonds are shown as red dotted lines with a cutoff of 3.5 Å.

Previous study has established cAPN as a functional receptor for four alpha-CoVs (FIPV-1146, TGEV, CCoV, and HCoV-229E), though the structural basis of the FIPV-1146 RBD binding to cAPN remains unresolved (Tusell et al, 2007). Here, we report the cryo-EM structure of the FIPV-1146 RBD-cAPN complex at 2.85 Å resolution (Appendix Figs. S8 and S9 and Appendix Table S1), revealing that the FIPV-1146 RBD, analogous to the FCoV-23 RBD, folds into a β-barrel flanked by two ligand-binding loops (Fig. 4). cAPN comprises four subdomains, with Domains II and IV mediating direct contact with the FIPV-1146 RBD. Consistent with dog APN (dAPN) bound to TGEV (Tian et al, 2025), cAPN also adopts an open conformation in the FIPV-1146 RBD-cAPN complex, characterized by a pronounced separation between Domains I and IV, likely facilitating viral engagement (Appendix Fig. S10).

Given that both FIPV-1146 and FCoV-23 are FIPV-II virus, we performed a comparative structural analysis of the complex formed between the spike RBD and cAPN in each virus (Fig. 4). Key residues contributing to hydrogen bonds (H-bonds) and van der Waals (vdw) interactions between cAPN and the RBDs of FIPV-1146 and FCoV-23 were also identified and labeled (Appendix Table S4). The RBDs of FIPV-1146 and FCoV-23 presented similar binding interface that directly interacted with cAPN, forming a total number of 218 and 219 dense vdw contacts, respectively, along with five hydrogen bonds in each case (Fig. 4A; Appendix Table S4). A total of 22 cAPN residues interact with the FIPV-1146 RBD (Appendix Fig. S12A), and the residues (R378, W749, T750, D751 and N795) of cAPN formed a hydrogen bond network with amino acids (K543, Y547, S553, W590 and D591) of the FIPV-1146 RBD (Fig. 4B,C). In contrast, FCoV-23 RBD engages 20 cAPN residues (Appendix Table S4), and a hydrogen-bond network is formed between cAPN residues (N748, W749, Q787 and N795) and the FCoV-23 RBD residues (S547, G548, Y549, Q551 and W592) (Fig. 4D,E).

Based on our previous finding that TGEV and PDCoV RBDs interact with dAPN in “open” and “closed” conformations, respectively (Tian et al, 2025), structural comparison reveals that cAPN similarly adopts an open conformation when bound to FIPV-1146 RBD, mirroring the TGEV RBD-dAPN interaction (Appendix Fig. S10). To determine whether the open conformation is critical for FIPV-1146 infection, we engineered a cAPN mutant (S424C-S929C) designed to form an intramolecular disulfide bond. This modification locks cAPN in a closed conformation, while simultaneously abolishing its enzymatic activity (Reguera et al, 2012; Santiago et al, 2017). Viral infection assays demonstrated that the cAPN-S424C-S929C mutant significantly impaired FIPV-1146/TGEV infection efficiency compared to the wild-type cAPN (Appendix Fig. S11).

### Cross-species recognition of FIPV-1146 to APN orthologs from 17 species

To investigate the potential for cross-species transmission of FIPV-1146, we selected 17 representative species across nine mammalian orders, including Carnivora (*Felis catus* [Cat], *Canis lupus familiaris* [Dog], *Vulpes vulpes* [Red fox], and *Ailuropoda melanoleuca* [Giant panda]), Pholidota (*Manis javanica* [Malayan pangolin]), Perissodactyla (*Equus caballus* [Horse]), Artiodactyla (*Bos taurus* [Bovine]), *Sus scrofa* [Pig], *Capra hircus* [(Goat], *Ovis aries* [Sheep], and *Camelus dromedarius* [Arabian camel]), Primates (*Homo sapiens* [Human]), Rodentia (*Mus musculus* [Mouse], and *Rattus norvegicus* [Rat]), Lagomorpha (*Oryctolagus cuniculus* [Rabbit]), Galliformes (*Gallus gallus* [Chicken]), Passeriformes (*Passer montanus* [Eurasian tree sparrow]). We conducted a sequence alignment analysis of the key residues in cAPN involved in the interaction with FIPV-1146 RBD from these 17 species (Appendix Fig. S12). The 22 key residues in cAPN responsible for binding to the FIPV-1146 RBD were highlighted and compared with 16 APN orthologs based on the complex structures in this study (Appendix Fig. S12A,B). Based on the APN amino acid sequences, we constructed a phylogenetic tree showing the genetic relationships among the 17 species. The number of residue substitutions in APN orthologs range from 4 to 12 compared to cAPN (Appendix Fig. S12B). From the residue comparison of the APNs, we found that the F739, W749, N795, and R802 sites were completely conserved among these 17 species. Residues at positions 751, 779, 783 and 798 differed the most frequently among the 17 APN orthologs (Appendix Fig. S12C).

**Figure 5:**
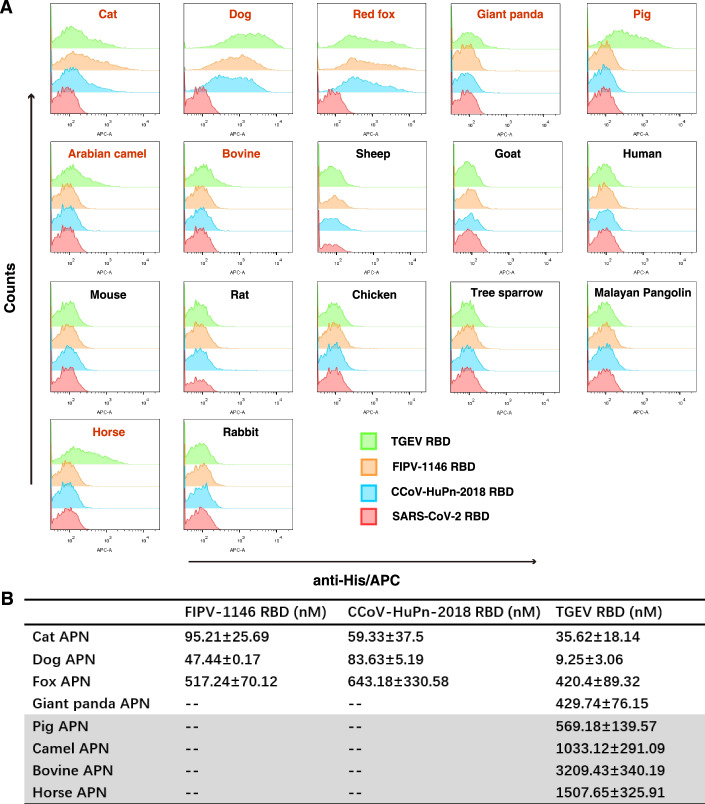
Characterization of binding of 17 APNs with the RBDs of FIPV-1146, CCoV-HuPn-2018 and TGEV. (**A**) His-tagged FIPV-1146, CCoV-HuPn-2018, TGEV and SARS-CoV-2 RBD proteins were incubated with BHK 21 cells expressing eGFP-fused APNs. Anti-His/APC antibody was used to detect cells expressing His-tagged protein. Cells stained with the TGEV RBD, FIPV-1146 RBD, CCoV-HuPn-2018 RBD and SARS-CoV-2 RBD proteins are shown in green, orange, marine, and red, respectively. The SARS-CoV-2 RBD was used as negative control. (**B**) The binding affinities of the three RBDs to the eight APNs were determined by SPR. Mean ± SD represents the mean and standard deviation of three independent experiments. Source data are available online for this figure.

To evaluate the cross-species receptor-binding potential of FIPV-1146, we compared the binding capabilities of the RBDs from three alpha-CoV-1 members (FIPV-1146, CCoV-HuPn-2018 and TGEV) to the APNs from 17 species, using flow cytometry (Fig. 5A). SARS-CoV-2 RBD that uses angiotensin-converting enzyme 2 (ACE2) as an invasion receptor was used as negative control. Flow cytometry results showed that the RBDs of all three viruses (FIPV-1146, CCoV-HuPn-2018 and TGEV) were capable of binding to cat, dog and red fox APNs. In contrast, no obvious interaction was detected for any of the three RBDs to APNs from Pholidota (Malayan pangolin), Artiodactyla (Goat and Sheep), Primates (Human), Rodentia (Mouse and Rat), Lagomorpha (Rabbit), Galliformes (Chicken), or Passeriformes (Eurasian tree sparrow). Notably, giant panda, pig, Arabian camel, bovine and horse APNs displayed different binding patterns. Specifically, whereas none of these five APNs interacted detectably with the RBDs of FIPV-1146 and CCoV-HuPn-2018, they all bound effectively to the TGEV RBD (Fig. 5A).

The binding affinities of the three viral RBDs (FIPV-1146, CCoV-HuPn-2018 and TGEV) for the eight APN orthologs were further assessed using surface plasmon resonance (SPR) (Fig. 5B; Appendix Fig. S13). All three RBDs could strongly bind to APNs from cats and dogs, with equilibrium dissociation constants (*K*_D_) of approximately 9-99 nM. In contrast to the strong binding to dog and cat APNs, red fox APN (rfAPN) exhibited a substantially weaker affinity (> fivefold lower) for the three viral RBDs. Furthermore, while APNs from giant panda, pig, Arabian camel, bovine and horse bound to the TGEV RBD with micromolar-level affinity, they showed no detectable binding to the RBDs of FIPV-1146 or CCoV-HuPn-2018.

### Susceptibility of FIPV-1146 mediated by APN orthologs

**Figure 6:**
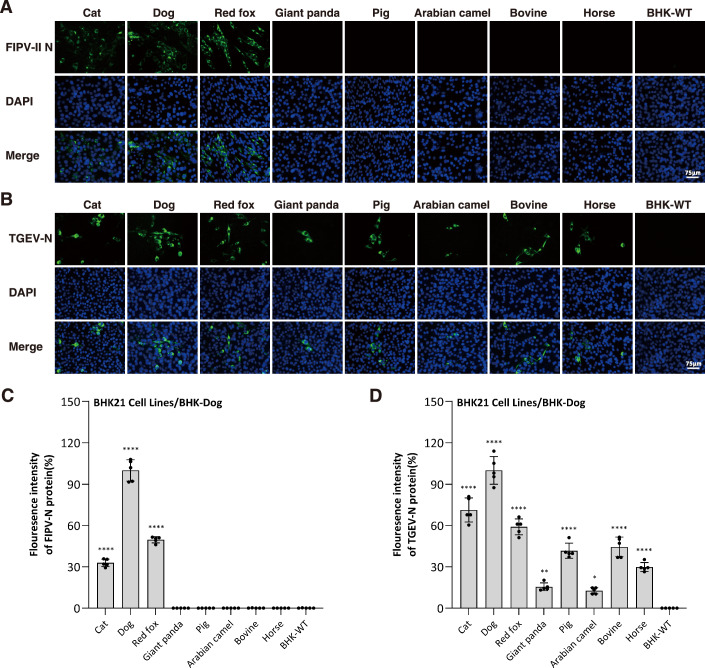
Susceptibility of FIPV-1146 and TGEV mediated by APN orthologs. (**A**) FIPV-1146 infection in BHK-21 cells overexpressing the APNs from eight species. Green fluorescence indicates BHK-21-APN cells infected with FIPV-1146. (**B**) TGEV infection in BHK-21 cells overexpressing the APNs from eight species. Green fluorescence indicates BHK-21-APN cells infected with TGEV. Wild-type BHK-21 cell is used as negative control. The scale bar indicates 75 μm. (**C**) The fluorescence intensity in (**A**) was determined by software ImageJ. All *P* values were obtained by comparing the indicated APN cell lines from different species with BHK-21-WT cells. Cat: *P* < 1 × 10^−15^, Dog: *P* < 1 × 10^−15^, Red fox: *P* < 1 × 10^−15^. (**D**) The fluorescence intensity in (**B**) was determined by software ImageJ. All *P* values were obtained by comparing the indicated APN cell lines from different species with BHK-21-WT cells. Cat: *P* < 1 × 10^−15^, Dog: *P* < 1 × 10^−15^, Red fox: *P* < 1 × 10^−15^, Giant panda: *P* = 1.41 × 10^−3^, Pig: *P* = 2.57 × 10^−12^, Arabian camel: *P* = 1.15 × 10^−2^, Bovine: *P* = 4.08 × 10^−13^, Horse: *P* = 1.38 × 10^−8^. Data are expressed as mean ± SD, *n* = 5. Error bars denote standard deviations for the samples. An unpaired two-tailed *t* test was used to determine the statistical significance. *P < 0.05; **P < 0.01; ***P < 0.001; ****P < 0.0001. Source data are available online for this figure.

The protein-binding assays revealed that the TGEV RBD exhibited a broader receptor-binding spectrum than the RBDs of FIPV-1146 and CCoV-HuPn-2018. To determine whether this correlates with actual infection potential, we performed direct infection assays using live viruses on BHK-21 cells stably expressing APNs from eight species: cat, dog, red fox, giant panda, bovine, horse, pig, and Arabian camel (Fig. 6). Due to the unavailability of live CCoV-HuPn-2018, only FIPV-1146 and TGEV were tested. Consistent with the SPR results, FIPV-1146 infection was supported only by cat, dog, and red fox APNs (Fig. 6A,C), whereas the eight APNs bound to the TGEV RBD were capable of mediating TGEV infection (Fig. 6B,D). No infection was observed in BHK-21 cells lacking APN expression. Notably, dAPN mediated the highest infection efficiency for both viruses (Fig. 6C,D).

### Identification of key residues governing cAPN binding to the FIPV-1146 RBD

To elucidate the molecular determinants of cAPN–FIPV-1146 RBD binding, we analyzed the APN substitutions at the interaction interface. It is well-established that APN serves as the functional receptor for the alpha-CoV-1, and N-linked glycosylation of APN (notably at N748) has been demonstrated to be a critical determinant for viral entry. For instance, the interaction between TGEV and pig APN (pAPN) is dependent on the N748-linked glycosylation (Reguera et al, 2012; Tian et al, 2025). Similarly, the recently identified CCoV-2018-HuPn and FCoV-23 also rely on glycosylated APN for host cell recognition (Tortorici et al, 2025; Tortorici et al, 2022). While pAPN retains the N748-linked glycan, it fails to bind the FIPV-1146 RBD, suggesting that divergent residues at the binding interface are also determinants. Comparative analysis identified four critical substitutions in cAPN compared to pAPN (P378R, E751D, Q776E and N779R) (Appendix Fig. S12B). We generated reciprocal cAPN mutants (R378P, D751E, E776Q and R779N) to mimic pAPN, alongside the T750R mutant (which abolishes N748-linked glycan) to assess its role. SPR results revealed that T750R abolished high-affinity FIPV-1146 RBD binding, consistent with its absence in non-susceptible species (human, goat, rabbit, mouse and rat), thus implying that N748-linked glycan is essential for both binding and host specificity (Appendix Fig. S14). While the E776Q mutant retains wild-type affinity, R378P, D751E and R779N exhibit approximately half the binding, accounting for the weaker binding of pAPN to the FIPV-1146 RBD than cAPN (Appendix Fig. S14).

### Identification of key residues responsible for the host range of FIPV-1146

**Figure 7:**
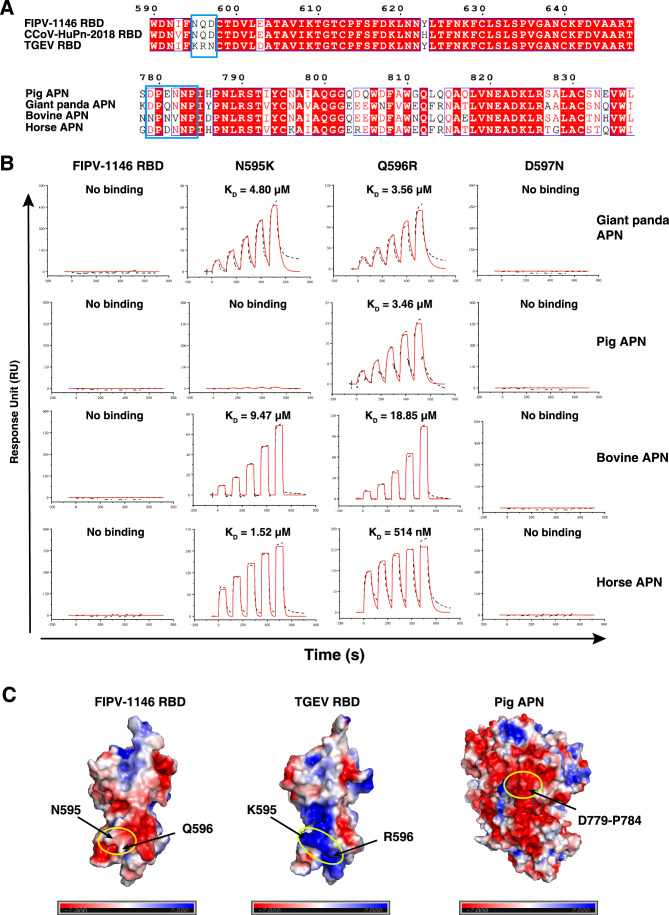
Identification of critical residues in FIPV-1146 RBD for host specificity. (**A**) Sequence alignment of RBDs (FIPV-1146, CCoV-HuPn-2018, and TGEV) with residues divergent in TGEV boxed in blue, and sequence alignment of APNs (pig, giant panda, bovine and horse) with residues interacting with RBD positions 595 and 596 boxed in blue. (**B**) The hFc-tagged APNs were captured by protein A chip, and sequentially tested the binding with serially diluted FIPV-1146 RBD mutants. The binding affinities of the APNs to the FIPV-1146 RBD mutants are shown. (**C**) Electrostatic surface views of FIPV-1146 RBD, TGEV RBD and pig APN. Residues N596-Q596 of FIPV-1146 RBD, and K595-R596 of TGEV RBD are circled with yellow ellipse. Residues from pig APN binding with the residue 595 and 596 of TGEV RBD is circled with yellow ellipse. Source data are available online for this figure.

Despite the high RBD homology among FIPV-1146, CCoV-HuPn-2018 and TGEV, the TGEV RBD has a broader receptor-binding spectrum than FIPV-1146 and CCoV-HuPn-2018 RBDs, which suggests that the divergent residues among the RBDs play a key role in affecting the host range. Notably, distinct loop residues (K595-R596-N597) were identified in the TGEV RBD adjacent to the RBD-APN binding region (Fig. 7A). To test its role, we engineered three FIPV-1146 RBD mutants (N595K, Q596R and D597N) to mimic the TGEV RBD, hypothesizing that this modification may affect APN engagement.

We then tested the binding between these mutants and the APN orthologs from pig, giant panda, bovine and horse, selected for their differential binding to FIPV-1146 versus TGEV. SPR assays revealed that while the wild-type FIPV-1146 RBD and the D597N mutant showed no detectable binding, the N595K and Q596R mutants acquired binding capacity to the APNs from giant panda (4.80 μM and 3.56 μM), pig (no binding and 3.46 μM), bovine (9.47 μM and 18.85 μM) and horse (1.52 μM and 514 nM) (Fig. 7B).

We next sought a structural rationale for the gain-of-function observed in the N595K and Q596R mutants. Structural analysis revealed that these residues face a positively charged patch on cAPN (corresponding to D_779_PENNP_784_ in pAPN) (Fig. 7A). The N595K and Q596R substitutions introduce a localized positive charge at this interface (Fig. 7C), which enhances the binding to pig, giant panda, bovine and horse APNs through electrostatic complementarity. Together, these results indicate that these two mutations are key determinants enabling FIPV-1146 to potentially overcome species barriers.

## Discussion

FIPV-II poses a significant threat to felids, with a high mortality rate and lack of effective vaccines, underscoring the urgent need to understand the molecular mechanisms of infection. As a member of the alpha-CoV family, FIPV-II shares structural and functional features with other members of alpha-CoV-1, yet exhibits distinct host specificity (Liu et al, 2023a; Tian et al, 2025). This study provides critical insights into the structural basis of FIPV-II interactions with its APN receptor and evaluates its potential for cross-species transmission.

Beta-CoV S proteins, including SARS-CoV, SARS-CoV-2 and MERS-CoV, employ dynamic RBDs that transition between “lying” and “standing” conformations to facilitate host cell entry (Wang et al, 2024; Wrapp et al, 2020; Yuan et al, 2017). This conformational flexibility, enabled by cross-subunit quaternary packing, allows RBD exposure for receptor engagement without steric constraints. However, such dynamics are not uniformly conserved across all members of beta-CoV. For instance, in HKU1 (a beta-CoV), RBD opening occurs only after binding to a primary sialoglycan receptor (Pronker et al, 2023), and the open conformation has not been observed for OC43 (Bangaru et al, 2022; Tortorici et al, 2019; Wang et al, 2022). Similarly, all reported gamma-CoV S structures have been captured exclusively in the closed state (Hulswit et al, 2026; Shang et al, 2018). Conversely, spontaneous RBD opening has been observed in the alpha-CoV Porcine epidemic diarrhea virus (PEDV) (Huang et al, 2022), indicating that RBD behavior does not strictly follow genus-level trends.

In line with this nuanced view, some alpha-CoVs (FCoV-23, CCoV-HuPn-2018 and type-I FIPV) and the delta-CoV PDCoV exhibit spikes that maintain RBDs persistently in the “lying” state, presenting an unresolved mechanistic paradox regarding their receptor recognition strategy (Tortorici et al, 2022; Wang et al, 2024; Yang et al, 2020). Our structural studies demonstrate that the free FIPV-1146 S trimer adopts a conserved propeller-like architecture and maintains a tightly packed conformation with RBDs locked in a “lying” state. The “lying” RBD sterically blocks APN binding, suggesting receptor engagement requires conformational changes in the S protein to expose the “standing” RBD. Moreover, we observed that a single cAPN dimer could simultaneously bind to two FIPV-1146 S trimers, which parallels the recently reported interaction pattern between HCoV-229E S and hAPN, even though the HCoV-229E S protein RBD adopts a lying state before binding to APN (Song et al, 2021; Tsai et al, 2025). Concurrently, we readily identified disassociated FIPV-1146 S1 trimer particles, which exhibit a ring-like structure with all three RBDs in a “standing” conformation, aligning with a similar conformation reported for MERS-CoV (Yuan et al, 2017). This observation suggests that the standing conformation of the three RBDs in the S1 trimer promotes its dissociation from the S2 moiety, making it rare to detect stable S trimers with all three RBDs in this conformation. Notably, we cannot rule out the possibility that the disassociated S1 trimer may simply be an experimental artifact.

The cryo-EM structure of the FIPV-1146 RBD bound to cAPN reveals a β-barrel RBD engaging cAPN through a network of H-bonds and vdw interactions, with residues R378, T750, D751, and R779 playing pivotal roles. The N748-linked glycan on cAPN has emerged as a critical determinant of binding, as its loss (T750R mutant) abolishes its binding to FIPV-1146 RBD. Despite the high sequence homology among the FIPV-1146, CCoV-HuPn-2018 and TGEV RBDs, their receptor-binding spectra varied significantly. TGEV exhibits a broader tropism due to the divergent interfacial residues. TGEV RBD binds to artiodactyl APNs (including pigs and bovines) with micromolar affinity, whereas FIPV-1146 does not engage these receptors. This disparity demonstrates that subtle structural differences, including changes in the charge properties of key residues (N595 and Q596 in FIPV-1146 RBD), dictate host specificity. Specifically, the N595K and Q596R substitutions broaden the potential host range by gaining binding capacity to APNs from pig, bovine, horse, and giant panda. Although current host range of FIPV-1146 remains restricted to felids and canids, the shared use of APN with other alpha-CoVs raises concerns regarding recombination-driven host jumps. Surveillance of FIPV-II variants with expanded tropism, particularly in multispecies environments, is warranted to mitigate zoonotic risks.

In summary, this study delineated the structural and functional basis of FIPV-II host specificity, highlighting the roles of the S trimer, cAPN glycosylation, and RBD-APN interactions in viral entry. These insights not only advance our understanding of FIPV-II pathogenesis, but also contribute to broader efforts to combat coronavirus zoonoses.

## Methods

**Table:** Reagents and tools table

Reagent/resource	Reference or source	Identifier or catalog number
**Experimental models**		
*Escherichia coli* (*E. coli*) strain DH5a	TIANGEN	Cat# CB101-02
HEK-293F cells	ATCC	ATCC CRL-3537
BHK-21	ATCC	ATCC CCL-10
LLC-PK1	ATCC	ATCC CL-101
CRFK	ATCC	ATCC CCL-94
TGEV SX413	Tian et al, 2025	GenBank: PQ603017
FIPV 79-1146	Provided by Dr. Wentao Li	GenBank: OQ311323.1
**Recombinant DNA**		
pEGFP-C1	MiaoLingPlasmid	Cat# P71405
pEGFP-C1-APN-Cat, accession number: P79171	This study	N/A
pEGFP-C1-APN-Dog, accession number: P79143	This study	N/A
pEGFP-C1-APN- Red fox, accession number: A0A3Q7SK15	This study	N/A
pEGFP-C1-APN- Giant panda, accession number: A0A7N5JPH8	This study	N/A
pEGFP-C1-APN-Malayan pangolin, accession number: UPI0008130BB4	This study	N/A
pEGFP-C1-APN- Pig, accession number: P15145	This study	N/A
pEGFP-C1-APN- Bovine, accession number: P79098	This study	N/A
pEGFP-C1-APN- Goat, accession number: A0A452E2A2	This study	N/A
pEGFP-C1-APN- Sheep, accession number: W5PQH2	This study	N/A
pEGFP-C1-APN-Horse, accession number: XP_008508865.1	This study	N/A
pEGFP-C1-APN- Arabian camel, accession number: XP_031296423.1	This study	N/A
pEGFP-C1-APN-Human, accession number: P15144	This study	N/A
pEGFP-C1-APN-Mouse, accession number: P97449	This study	N/A
pEGFP-C1-APN-Rat, accession number: P15684	This study	N/A
pEGFP-C1-APN-Rabbit, accession number: P15541	This study	N/A
pEGFP-C1-APN-Chicken, accession number: O57579	This study	N/A
pEGFP-C1-APN- Eurasian tree sparrow, accession number: XP_039579479.1	This study	N/A
pCAGGS	MiaoLingPlasmid	Cat# P0165
pCAGGS-FIPV-1146-S-Strep-His, spike residues 1–1452, accession number: OQ311323.1	This study	N/A
pCAGGS-FIPV-1146-RBD-His, spike residues 500–674, accession number: OQ311323.1	This study	N/A
pCAGGS-FIPV- CCoV-HuPn-2018-RBD-His, spike residues 496–670, accession number: QVL91811.1	This study	N/A
pCAGGS- TGEV-RBD-His, spike residues 496–670, accession number: OQ311323.1	This study	N/A
pCAGGS-APN-Cat-hFc, accession number: P79171	This study	N/A
pCAGGS-APN-Dog-hFc, accession number: P79143	This study	N/A
pCAGGS-APN-Red fox-hFc, accession number: A0A3Q7SK15	This study	N/A
pCAGGS-APN-Giant panda-hFc, accession number: A0A7N5JPH8	This study	N/A
pCAGGS-APN-Pig-hFc, accession number: P15145	This study	N/A
pCAGGS-APN-Arabian camel -hFc, accession number: XP_031296423.1	This study	N/A
pCAGGS-APN-Bovine-hFc, accession number: P79098	This study	N/A
pCAGGS-APN-Horse -hFc, accession number: XP_008508865.1	This study	N/A
pcDNA4.0-APN-Cat-FLAG, accession number: P79171	This study	N/A
pcDNA4.0-APN-Dog-FLAG, accession number: P79143	This study	N/A
pcDNA4.0-APN-Red fox-FLAG, accession number: A0A3Q7SK15	This study	N/A
pcDNA4.0-APN- Giant panda-FLAG, accession number: A0A7N5JPH8	This study	N/A
pcDNA4.0-APN- Pig-FLAG, accession number: P15145	This study	N/A
pcDNA4.0-APN- Bovine-FLAG, accession number: P79098	This study	N/A
pcDNA4.0-APN- Arabian camel-FLAG, accession number: XP_031296423.1	This study	N/A
pcDNA4.0-APN-Horse-FLAG, accession number: XP_008508865.1	This study	N/A
**Antibodies**		
Anti-TGEV N mAb, for IFA, dilution ratio 1:300	Shandong Lvdu Bio-Sciences	Cat#LANDU-153
FITC-labeled goat anti-mouse IgG, for IFA, dilution ratio 1:1000	Abcam	Cat#ab6785
anti-His/APC antibodies, for flow cytometry, dilution ratio 1:500	Miltenyi Biotec	Cat# 130-119-820; RRID: AB_2751870
**Chemicals, enzymes and other reagents**		
PEI	Yeasen	Cat#40816ES03
DAPI stain Solution	Coolaber	Cat#SL7101-1mL
Triton X-100	Colarbio	CAS: 9002-93-1
4% Paraformaldehyde	Biosharp	Cat#BL539A
Zeocin Solution	Coolaber	Cat#SL4140
Glycine	Coolaber	Cat#CG5831
Urea	Sigma-Aldrich	Cat#U5128
Iodoacetamide	Sigma-Aldrich	Cat#I6125
DL-Dithiothreitol	Sigma-Aldrich	Cat#D0632
Trypsin Gold, Mass Spectrometry Grade	Promega	Cat#V5280
Lys-C, Sequencing Grade	Roche	Cat#11420429001
Chymotrypsin, Sequencing Grade	Promega	Cat#V1061
Elastase	Promega	Cat#V1891
Glu-C (Sequencing Grade)	Promega	Cat#V1651
α-Lytic protease	New England Biolabs	Cat#P0761S
Peptide-N-glycosidase F (PNGase F)	This study	N/A
^18^O-water (97 atom %18 O)	Sigma-Aldrich	Cat#329878
O-glycoprotease (IMPa)	New England Biolabs	Cat#P8113S
Trypsin Gold, Mass Spectrometry Grade	Promega	Cat#V5280
Lys-C, Sequencing Grade	Roche	Cat#11420429001
Chymotrypsin, Sequencing Grade	Promega	Cat#V1061
Acetonitrile Optima^TM^ UHPLC/MS Grade	ThermoFisher	Cat#A9561
Water, LC-MS grade	ThermoFisher	Cat#047146.K7
Ammonium acetate	Sigma-Aldrich	Cat#431311
Formic acid Optima^TM^ LC/MS Grade	ThermoFisher	Cat#A117-05AMP
Acetic acid	Aladdin	Cat#A116166
Trifluoroacetic acid	Sigma-Aldrich	Cat#T6508
SMM 293-TII Expression Medium	Sinobiological	Cat#M293TII
DMEM	Gibco	Cat#C11995500BT
Fetal Bovine Serum	Excellbio	Cat#FSP500
Nonfat dried milk	Coolaber	Cat#CN7861
QuickCut EcoR I	TaKaRa	Cat#Code No. 1611
QuickCut Xho I	TaKaRa	Cat#Code No. 1635
**Software**		
PyMOL v.2.0	https://pymol.org/	MolecularGraphicsSystem
FlowJo V10	https://www.flowjo.com/flowjo10/download	
BIAcore8KEvaluationsoftware	GE Healthcare	N/A
Byonic™ v 5.2.5	https://www.proteinmetrics.com/products/byonic	
EPU	ThermoFisher Scientific	N/A
cryoSPARC v.4.2.0	https://cryosparc.com/download	
Topaz	https://www.topazlabs.com/	
DeepEMhancer	Sanchez-Garcia et al, 2021	
UCSF Chimera v.1.15	https://www.cgl.ucsf.edu/chimera/download.htmld	
FlowJo V10	https://www.flowjo.com/solutions/flowjo/downloads	
Motioncor2	Zheng et al, 2017	N/A
COOT v.0.9.3	https://www2.mrc-lmb.cam.ac.uk/personal/peemsley/coot/	
Phenix v.1.20.1	http://www.phenix-online.org/	
MolProbity	http://molprobity.biochem.duke.edu/index.php	
**Other**		
Protein A sensor chip	Cytiva	SKU: 29127555
PepMap™ Neo C18, 300μm*5 mm, 5μm, 100 Å	ThermoFisher	Cat#174500
Acclaim™ PepMap™ 100 C18, 75μm*25 cm, 2μm, 100 Å	ThermoFisher	Cat#164941
Sep-Pak C18 cartridge (200 mg sorbent, 3 cc)	Waters	Cat#WAT094225
HisTrap excel	Cytiva	SKU: 17371206
Superdex 200 Increase 10/300 GL	Cytiva	Cat#28990944
HiTrap Protein A HP	Cytiva	Cat#17040301
300 kV Titan Krios	ThermoFisher	
Vitrobot Mark IV System	ThermoFisher	

### Cells

HEK293F cells (Gibco, Cat#11625-019) were cultured in SMM 293-TII medium (Sinobiological) at 37 °C and 5% CO_2_, shaking with 140 rpm. BHK-21 cells (ATCC CCL-10) were cultured in Dulbecco’s Modified Eagle medium (DMEM) containing 10% fetal bovine serum (FBS) at 37 °C and 5% CO_2_. TGEV SX413 strain (GenBank accession No. PQ603017) were isolated from the piglets suffering severe diarrhea and passaged in LLC-PK1 cells with DMEM supplemented with 10 μg/ml trypsin. FIPV 79-1146 (GenBank accession No. OQ311323.1) was passaged in CRFK cells with DMEM at 37 °C and 5% CO_2_.

### Gene cloning

Gene sequence encoding FIPV-1146 S protein (residues 1–1452, GenBank accession no. OQ311323.1) used in this study was synthesized by GENEWIZ, codon optimized for expression in mammalian cells, cloned into pCAGGS vectors between *Eco*RI and *Xho*I, in frame with a Kozak’s sequence to direct translation, with the signal peptide derived from the interleukin-2, and a Strep-II tag and a C-terminal octa-histidine tag. To stabilize the S protein in prefusion conformation, residues E (1144) and L (1145) were mutated to P, as previously described (Niu et al, 2024).

Full-length coding sequences of APN orthologs from 17 animals were synthesized and cloned into the pEGFP-C1 vector. Separately, the ectodomains of these APNs, fused to the human IgG Fc domain (hFc), were synthesized and cloned into pCAGGS vectors, as previously described (Tian et al, 2025). The coding sequences of FIPV-1146 RBD (residues 500–674, GenBank accession no. OQ311323.1), CCoV-HuPn-2018 RBD (residues 496–670, GenBank accession no. QVL91811.1) and TGEV RBD (residues 496–670, GenBank accession no. AJ271965) and cAPN ectodomain were cloned into pCAGGS vectors with a C-terminal six histidine tag. An interleukin-2 signal peptide sequence was added to the N-terminus of each construct to facilitate protein secretion.

### Protein expression and purification

The viral RBDs of FIPV-1146, CCoV-HuPn-2018 and TGEV with his tag were expressed and purified from the culture supernatants of HEK293F cells using a His-Trap Excel column (GE Healthcare) and further purified by Superdex^TM^ 200 Increase 10/300 GL column (GE Healthcare). Purified proteins were stored in protein buffer (20 mM Tris-HCl, pH 8.0 and 150 mM NaCl). To prepare the hFc-tagged APN proteins, the pCAGGS plasmids containing the coding sequences of APNs were transiently transfected into HEK293F cells. Five days later, supernatant containing the indicated protein were collected and purified using a Protein A column (GE Healthcare), and then used for SPR assays.

### Enzymes used in glycoproteomic analysis

Trypsin gold (mass spectrometry grade), chymotrypsin (sequencing grade), elastase, Glu-C (sequencing grade) were purchased from Promega; Lys-C (sequencing grade) was purchased from Roche; α-lytic protease and *O*-glycoprotease (IMPa) were purchased from New England Biolabs. ^18^O-water (97 atom % ^18^O) was purchased from Sigma-Aldrich. Peptide-N-glycosidase (PNGase F) was prepared in-house.

### Preparation of glycopeptides for mass spectrometry analysis

In total, 200 μg of purified FIPV-1146 S protein was buffer exchanged in 100 mM ammonium acetate solution (pH 6.0) using a 10 kDa MWCO mass filter (Sigma-Aldrich). Next, the protein was denatured for 1 h in 100 mM ammonium bicarbonate (pH 8.0) containing 6 M urea at room temperature. Protein was then reduced in 1,4-dithiothreitol (Sigma-Aldrich) to a final concentration of 5 mM at 56 °C for 1 h. The S protein was then alkylated by adding iodoacetamide (Sigma-Aldrich) to a final concentration of 10 mM and incubated for 1 h in the dark. The alkylated S protein was buffer-exchanged into 100 mM ammonium acetate (pH 8.0) and digested separately using trypsin/LysC, chymotrypsin (with 10 mM CaCl_2_), Glu-C, elastase, and α-lytic protease at a ratio of 1:30 (w/w) at 37 °C for 18 h.

The next day, the peptides were desalted using a Sep-Pak C18 cartridge (200 mg sorbent, 3 cc, Waters). Briefly, the cartridge was washed with 3 mL of 100% acetonitrile, conditioned with 1 mL of 50% acetonitrile (ACN) with 0.5% acetic acid, and equilibrated with 3 mL of 0.1% trifluoroacetic acid (TFA) in water. The sample was dissolved in 200 μL of 0.4% TFA water, then loaded onto the cartridge, desalted with 3 mL of 0.1% TFA and 300 μL of 0.5% acetic acid, and eluted with 2 mL of 50% ACN with 0.5% acetic acid and 2 mL of 95% ACN with 0.5% acetic acid. The eluant was collected, lyophilized, and re-suspended in water. To denature the proteases, the peptide solutions were then heated at 100 °C for 30 s, then cooled down at room temperature for 30 s, and the process was repeated in 10 min.

Each peptide sample treated with each protease was divided into three aliquots: one directly used for glycopeptide analysis, one treated with *O*-glycoprotease (IMPa), and one treated with PNGase F in ^18^O-water. The first aliquot was then lyophilized and re-suspended in 20 μL of 0.1% formic acid in water, then analyzed by LC-MS/MS. The second aliquot of peptides was lyophilized, then re-dissolved in 20 μL of 100 mM ammonium bicarbonate (pH 8.0), and IMPa was added to the peptide mixture at a minimum enzyme/glycoprotein ratio of 10 NEB units per 10 μg. Then, the solution was incubated at 37 °C for 5 h, then lyophilized and re-suspended in 0.1% formic acid in water. The third aliquot of peptides was lyophilized and then re-dissolved in 20 μL of 100 mM ammonium bicarbonate (pH 8.0) in ^18^O-water. An aliquot of PNGase F was lyophilized and reconstituted in H_2_^18^O and added into the protein solution with a ratio of 1:50 (w/w) at 37 °C in 5 h in sealed tube to avoid back-exchange of atmospheric water.

### LC-MS/MS analysis

The peptides were first separated with a Thermo Scientific Vanquish^TM^ Neo UHPLC system using trap-elute mode and then emitted into a Thermo Scientific Orbitrap Eclipse mass spectrometer (Thermo Fisher, San Jose).

Solvent A was 0.1% formic acid in water, while solvent B was 0.1% formic acid in 80% acetonitrile. After loading for 3 min with a flow rate of 10 μL/min in the trap column (Thermo Scientific™ PepMap™ Neo C18, 300 μm*5 mm, 5 μm, 100 Å), all of the peptides were further eluted in the analytical column (Thermo Scientific™ Acclaim™ PepMap™ 100 C18, 75 μm*25 cm, 2 μm, 100 Å) with a flow rate of 250 nL/min using a gradient of 3% B for 6 min, 3% to 25% B for 80 min, 25% to 40% B for 16 min, and 40% to 95% B for 4 min and 95% for 10 min. This was followed by a 10 min washing step and a 10 min column equilibration step. Columns were kept at room temperature. Experiments were performed in positive ion mode with peptide application mode. Spray voltage was 1800 V, ion transfer tube temperature was 250 °C.

To achieve glycopeptide identification, the stepped collision energy (SCE) higher-energy dissociation (HCD), and HCD product ions-triggered electron-transfer/higher-energy collision dissociation (HCDpdEThcD) fragmentation methods were employed for the data acquisition. In the SCE method, the full scan mass spectra were recorded with a scan range from 350 to 2000 *m/z* with a 120,000 resolution (at 200 *m/z*), maximum injection time of 50 ms, AGC target of 10^5^, microscan of 1, and RF lens of 50%. A top 3 setting was used for the data-dependent (dd)-MS2 scan with a 15 s dynamic exclusion and mass tolerance of 10 ppm. In quadrupole isolation mode, the isolation window was set at 2.0 *m/z*. Activation type was HCD with stepped collision energy mode. The HCD collisional energy was applied with 10%, 15%, 20%, 25%, and 30%. The MS2 scan range was set at 150 to 2000 *m/z* with a resolution of 30,000, maximum injection time of 150 ms, AGC target of 5 × 10^4^, and microscan of 1.

In HCDpdEThcD mode, the full scan mass spectra were recorded over a scan range from 350 to 2000 *m/z* with a 60,000 resolution (at 200 *m/z*). The AGC target and maximum injection time were set to 4 × 10^5^ and 50 ms. A top 3 setting was used for the dd-MS2 scan with a 20 s dynamic exclusion and mass tolerance of 10 ppm, including charge states from 2 to 8. The precursor priority was set at the highest charge state. Activation type was HCD with fixed collision energy mode. The HCD-MS2 scan range was set at 100 to 2000 *m/z* with a resolution of 30,000, maximum injection time of 54 ms, AGC target of 5 × 10^4^, and microscan of 1. The product-dependent EThcD was triggered on the same precursor ion of HCD fragmentation if at least two of the specific masses (126.0550, 138.0549, 144.0655, 163.0801, 168.0654, 186.0760, 204.0865, 243.0260, 274.0921, 292.1027, 325.1120, 366.1395) were hit in the HCD spectra with top N product ions of 20 and mass tolerance of 15 ppm. Calibrated charge-dependent ETD parameters and 25% NCE HCD supplemental activation was used to obtain the EThcD spectra with a mass range of 200–4000 *m/z*, resolution set to 60,000 (at 200 *m/z*), AGC target set to 10^5^, and maximum injection time set to 400 ms.

### Glycopeptide fragmentation data analysis

The glycopeptide fragmentation data were extracted from the raw file using Byonic^TM^ (Version 5.2.5) software with the mass tolerance for the precursors and fragment ions set at ±10 ppm and ±0.05 Da, respectively. The following cleavage sites were used for the respective proteases; trypsin=R/K (C-terminal), LysC=K (C-terminal), chymotrypsin=F/Y/W (C-terminal), α-lytic protease=T/A/S/V (C-terminal), Glu-C = E/D (C-terminal), elastase = A/V/S/G/L/I (C-terminal), IMPa=S/T (N-terminal). The number of missed cleavages was set at 2. The fixed modification was carbamidomethyl (C, +57.021464), the variable modifications included oxidation (M, +15.994915), and additional deamidation (N, +0.984016) for H_2_^16^O and deamidation (N, +2.988261) for H_2_^18^O, was added for the *O*-glycosite search. In addition, the Protein Metrics 70 O-linked glycan library and 309 *N*-linked glycan library were specified as *O*-glycan modifications for the intact *O*-glycopeptides and the *N*-glycan modifications for the intact *N*-glycopeptides. A 1% false discovery rate (FDR) was applied. For the *N*-glycosylation identification, in addition to the NXS/T motif, the variable modification at any *N* was set in the modification panel, and the 309 *N*-linked glycan library was used.

Four criteria were used for the characterization of *O*-glycopeptides: (1) the MS/MS spectra contain glycan or oxonium ion (i.e., feature b ions); (2) the MS/MS spectra contain feature y ions; (3) the isotope distribution of the precursor is reasonable; and (4) the retention time of glycopeptides and unglycopeptides is comparable. The peptide score was set at 150 for filtering *O*- and *N*-glycopeptides, respectively. The relative amounts of each *O*-glycosite were calculated by comparing the extracted chromatographic areas of glycosylated peptides to the total peptides containing the modified glycosites. Based on the retention times and *m/z* values, intensities of all glycopeptides were manually checked for areas and corrected for charge states and all charge states for a single glycopeptide were summed. Furthermore, all of the glycopeptide-spectrum matches must be manually examined in order to select the valid *O*-glycopeptides for the glycosites analysis. Diagnostic ions were used as the requisite criterion for the *O*-glycosites determination.

HexNAc(2)Hex(5-9)Fuc(0 − 1) was classified as high mannose *N*-glycans. HexNAc(3)Hex(4 − 6)Fuc(0 − 1)X was classified as hybrid N-glycans. Other *N*-glycan structures were classified as complex-type *N*-glycans. Any glycan containing at least one sialic acid was counted as sialylated.

### Flow cytometry

For the flow cytometry assays, plasmids containing APN orthologs fused to eGFP were transfected into BHK-21 cells. The cells were collected after 24 h and washed twice with PBS (with 0.5% FBS) and then separately incubated with 10 μg/mL of the test proteins (the RBDs of FIPV-1146, CCoV-HuPn-2018, TGEV and SARS-CoV-2) with histidine tags at 37 °C for 30 min. After washing, cells were stained for 30 min at 37 °C with anti-His/APC antibodies (1:500, Miltenyi Biotec, AB_2751870). Cells were then washed three times, and flow cytometry data were acquired on a BD FACSCanto. Binding efficiencies are expressed as the percent of cells positive for RBD-His among the GFP-positive cells (APN-expressing cells). The figures were generated using FlowJo V10 software.

### SPR assay

The eight hFc-tagged APNs or the APN mutants were respectively captured on flow cell 2 of the protein A sensor chip (GE Healthcare) at more than 1000 response units. Flow cell 1 was used as the negative control. Then, serially diluted FIPV-1146, CCoV-HuPn-2018 and TGEV RBDs flowed over the chip in PBST buffer. Response units (RU) were measured with a BIAcore 8 K (GE Healthcare) in single-cycle mode. The antibodies were regenerated with 10 mM glycine-HCl (pH 1.7). The equilibrium dissociation constants (*K*_D_) of each pair of interaction were calculated using BIAcore 8 K Evaluation Software (GE Healthcare) by fitting to a 1:1 Langmuir binding model. Data is presented as mean ± SD of three independent results.

### Construction of BHK-21-APN stable cell lines

The wild-type or mutant APN genes were individually cloned into a pcDNA4.0 plasmid with a C-terminal Flag tag and transfected into BHK-21 cells at 90% confluency in six-well plates. After 48 h of culture, the cells were passaged and the medium was replaced with complete DMEM containing 200 μg/mL Zeocin. Following one week of selection, single colony cells were collected.

### Indirect immunofluorescence (IFA)

For IFA assay, cells infected with FIPV-1146/TGEV in 24-well plates were washed with PBS and fixed with 4% paraformaldehyde for 30 min. The cells were then permeabilized with 0.1% Triton X-100 for 10 min and blocked with 5% skim milk at 37 °C for 1 h. After washing three times with PBS, the cells were incubated with primary antibodies: for FIPV-1146-infected cells, a 1:1000 dilution of anti-FIPV-II N monoclonal antibody was applied at 37 °C for 1 h, while for TGEV-infected cells, a 1:300 dilution of anti-TGEV N monoclonal antibody was used under the same conditions. Following five washes with PBS, the cells were incubated with a secondary antibody (a 1:1000 dilution of FITC-conjugated goat anti-mouse IgG, Abcam, Cat# ab6785) at 37 °C for 1 h. Finally, the cells were stained with 4’,6-diamidino-2-phenylindole (DAPI) for 10 min and washed three times. Fluorescence images were captured using a fluorescence microscope (Leica), and fluorescence intensity was quantified using ImageJ software. Each group included three replicates, and statistical significance was determined by an unpaired two-sample *t* test.

### Cryo-EM sample preparation and data acquisition

The cryo-samples of FIPV-1146 S, FIPV-1146 RBD-cAPN and FIPV-1146 S-cAPN complex were vitrified using a Vitrobot Mark IV (Thermo Fisher Scientific) plunge-freezing device. Specifically, the FIPV-1146 S sample (4.0 μL, 4.9 mg/ml) was applied to a Cu Quantifoil 1.2/1.3 holey carbon grid that had been glow-discharged for 40 s. The grids were then blotted for 3 s with a blot force of −5. The FIPV-1146 S-cAPN sample (4.0 μL, 1.6 mg/ml) was also applied to a Cu Quantifoil 1.2/1.3 holey carbon grid, and blotted for 4 s with a blot force of −4. The FIPV-1146 RBD-cAPN sample (4.0 μL, 0.5 mg/ml) was applied to a graphene oxide coated grid (R1.2/1.3 300 mesh) and blotted for 2 s with a blot force of −2. The two FIPV-1146 S-cAPN sample was applied to a Cu Quantifoil 1.2/1.3 holey carbon grid and blotted for 4 s with a blot force of −4. All samples were maintained at a temperature of 4 °C and a humidity level of >99%, and subsequently plunge-frozen into liquid ethane.

The prepared grids were transferred to a 300 kV Titan Krios transmission electron microscope equipped with Gatan K3 detector and GIF Quantum energy filter. The FIPV-1146 RBD-cAPN movies were acquired at ×105,000 magnification with a calibrated pixel size of 0.69 Å, using a defocus range of −1.0 to −2.0 μm in super-resolution counting mode, with a total electron dose of 60 e^-^/Å². The FIPV-1146 S trimer movies were collected at ×105,000 magnification with a calibrated pixel size of 0.81 Å over a defocus range of −1.0 to −2.0 μm in super-resolution counting mode with a total dose of 50 e^-^/Å^2^. The FIPV-1146 S-cAPN movies were collected at ×81,000 magnification with a calibrated pixel size of 0.86 Å over a defocus range of −1.0 to −2.0 μm in super-resolution counting mode with a total dose of 50 e^-^/Å^2^. The two FIPV-1146 S-cAPN movies were collected at ×105,000 magnification with a calibrated pixel size of 0.81 Å over a defocus range of −1.0 to −2.0 μm in super-resolution counting mode with a total dose of 50 e^-^/Å^2^ using EPU (ThermoFisher Scientific) automated acquisition software.

### Image processing

The detailed data processing workflow is summarized in Appendix Figs. S1, S6, S7 and S8. All raw dose-fractionated image stacks were binned 2×, aligned, dose-weighted, and summed using MotionCor2 (Zheng et al, 2017). Contrast transfer function (CTF) estimation, particle picking and extraction, 2D classification, ab initio model generation, and 3D refinements were performed in cryoSPARC v.4.2.0 (Punjani et al, 2017).

For the FIPV-1146 RBD-cAPN complex, a total of 6302 micrographs were collected for this dataset. Particles were initially picked using the blob-pick procedure of cryoSPARC from 1000 micrographs and then subjected to 2D classification. After three rounds of 2D classification, well-defined particles from various orientations were selected for Topaz training to generate the Topaz model. The Topaz procedure (Bepler et al, 2019) was then applied to pick particles from the entire micrograph dataset, The 731,876 initial particles were picked and extracted from 6302 micrographs. After the extensive 2D classification, approximately 599,700 good particles were selected to heterogeneous refinement. Among the six resulted classes, one dominant class containing 54.26% of total particles was identified, which displayed clear features of secondary structural elements, especially in the area of the binding interface of the FIPV-1146 RBD and cAPN. These particles were subjected to non-uniform refinement. We also conducted one round of local refinement focused on the RBD-APN binding interface to improve the map quality and finally yielded a 2.85 Å cryo-EM map for the prototype RBD-APN subcomplex structure. The final map was sharpened by DeepEMhancer (Sanchez-Garcia et al, 2021).

For the FIPV-1146 S trimer dataset, the image processing was similar. A total of 412,108 initial particles were picked and extracted from 8858 micrographs. After extensive 2D classification, ~221,937 good particles were selected for heterogeneous refinement. Among six classes, one dominant class contained 18.3% of the total particles and displayed FIPV-1146 S1 trimer features. one dominant class contained 37.7% of the total particles and displayed clear features of secondary structural elements. The class was processed with homogeneous refinement, yielding final density map at 2.78 Å resolution.

To investigate the structural changes of the FIPV-1146 S protein upon binding to cAPN, cryo-EM data were collected for the FIPV-1146 S-cAPN complex. A total of 293,525 initial particles were picked and extracted from 8357 micrographs. Following extensive 2D classification, ~206,665 selected particles underwent heterogeneous refinement, yielding three classes. One dominant class, containing 60.3% of particles and displaying clear complex features, was selected for further 3D classification. This step produced three subclasses, with the dominant one (56.91% of particles) showing one FIPV-1146 S-cAPN complex features. This particle set was subjected to non-uniform refinement, resulting in a final density map at 5.09 Å resolution. Additional cryo-EM data were collected to capture different conformational states of the FIPV-1146 S-cAPN complex. From 12,483 micrographs, 515,745 initial particles were extracted. After 2D classification, approximately 275,427 particles were selected for heterogeneous refinement. Among the three resulting classes, one class revealed a structure in which a dimeric APN binds two S proteins; this class was refined to a resolution of 3.53 Å using homogeneous refinement. Another class displayed the apo conformation of the S protein. To obtain further conformational details, 3D classification was performed on selected particles. One subclass (class b, comprising 40% of particles) exhibited an S trimer in a mixed D0 conformation. This class was refined via homogeneous refinement, yielding a map at 2.86 Å resolution.

### Model building and structure refinement

For the initial model building of the FIPV-1146 RBD-cAPN complex, a structure with PDB code 8Z27 was used. For the initial model building of the FIPV-1146 S trimer, a structure with PDB code 7USA was used, as the starting model and fitted into the corresponding cryo-EM maps using UCSF Chimera v.1.15 (Pettersen et al, 2004). Mutations and manual adjustments were carried out using COOT v.0.9.3 (Emsley et al, 2010). Glycans were added at N-linked glycosylation sites in COOT. Each residue was manually checked, considering its chemical properties during model building. Several rounds of real-space refinement in Phenix v.1.20.1 (Adams et al, 2010) and manual building in COOT were performed until the final reliable models were obtained. Molprobity (Chen et al, 2010) was used to validate geometry and check structure quality. Statistics associated with data collection, 3D reconstruction and model building were summarized in Appendix Table S1. Figures were generated using Chimera (Pettersen et al, 2004) and PyMol v.2.0 (http://www.pymol.org).

## Supplementary information

Appendix

Peer Review File

Source data Fig. 3

Source data Fig. 5

Source data Fig. 6

Source data Fig. 7

## Data Availability

The raw LC-MS/MS data and associated Byonic project files have been deposited in the ProteomeXchange Consortium via the iProX partner repository under the dataset identifier PXD077720 (iProX - integrated Proteome resources). The EM density maps and corresponding atomic coordinates have been deposited in the Electron Microscopy Data Bank (EMDB) and Protein Data Bank (PDB), respectively. The accession numbers for the cryo-EM structures reported in this paper are as follows: FIPV-1146 S: EMD-64076; 9UE3. FIPV-1146 RBD-cAPN: EMD-64088; 9UEP. One FIPV-1146 S bound to cAPN dimer: EMD-64643. Two FIPV-1146 S bound to cAPN dimer: EMD-67259. Dissociated FIPV-1146 S1 trimer: EMD-67260. FIPV-1146 S with mixed D0 conformation: EMD-67940. FIPV-1146 S1 trimer-cAPN: EMD-67258. The source data of this paper are collected in the following database record: biostudies:S-SCDT-10_1038-S44318-026-00849-2.
