# Supplementary material for: Structure and receptor recognition of type-II feline infectious peritonitis virus spike glycoprotein
Source: EMBO J. 2026 Jul 8;45(15):5537–55. doi: 10.1038/s44318-026-00849-2 (PMC13434051; doi:10.1038/s44318-026-00849-2)
Supplement: Supplementary file 1 [file 44318_2026_849_MOESM1_ESM.pdf]

Appendix for

Structure and receptor recognition of type-II feline infectious peritonitis virus spike glycoprotein

Table of content

Appendix Figure S1. EM data processing of FIPV-1146 S trimer. 3

Appendix Figure S2. Cryo-EM densities of representative glycans on FIPV-1146 S and their modeled atomic coordinates. 4

Appendix Figure S3. Sequence alignment of the FIPV-1146 and FCoV-23 S used in this study. 5

Appendix Figure S4. Multiple alignments of FIPV-1146 S and the relevant structures. 6

Appendix Figure S5. Low resolution cryo-EM structure of the disassociated FIPV-1146 S1 trimer. 7

Appendix Figure S6. EM data processing of the complex comprising one FIPV-1146 S trimer bound to cAPN dimer. 9

Appendix Figure S7. EM data processing of the complex comprising two FIPV-1146 S trimer bound to cAPN dimer. 11

Appendix Figure S8. EM data processing of FIPV-1146 RBD-cAPN complex. 12

Appendix Figure S9. Representative electron density maps at the binding interface, related to Figure 4 13

Appendix Figure S10. Overall structure of APNs in complex with FIPV-1146, TGEV and PDCoV RBDs. 14

Appendix Figure S11. Mutational analysis of S464C+S929C in cAPN involved in the susceptibility of FIPV-1146 and TGEV. 15

Appendix Figure S12. Sequence alignment of 17 animal APNs, based on 22 APN residues involved in FIPV-1146 RBD binding. 16

Appendix Figure S13. Binding curves of eight APNs to the RBDs from TGEV, FIPV-1146 and CCoV-HuPn-2018, related to Figure 5B. 17

Appendix Figure S14. Binding affinity assay between the cAPN mutants and FIPV-1146 RBD by SPR. 18

Appendix Table S1. Cryo-EM data collection, refinement and validation statistics 19

Appendix Table S2. A summary of N-glycosylation sites of FIPV-1146 S observed experimentally through mass spectrometry and cryo-EM. 20

Appendix Table S3. A summary of O-glycosylation sites of FIPV-1146 S observed experimentally through mass spectrometry. 22

Appendix Table S4. The interaction of cat APN with the RBDs of FIPV-1146 and FCoV-23. 23

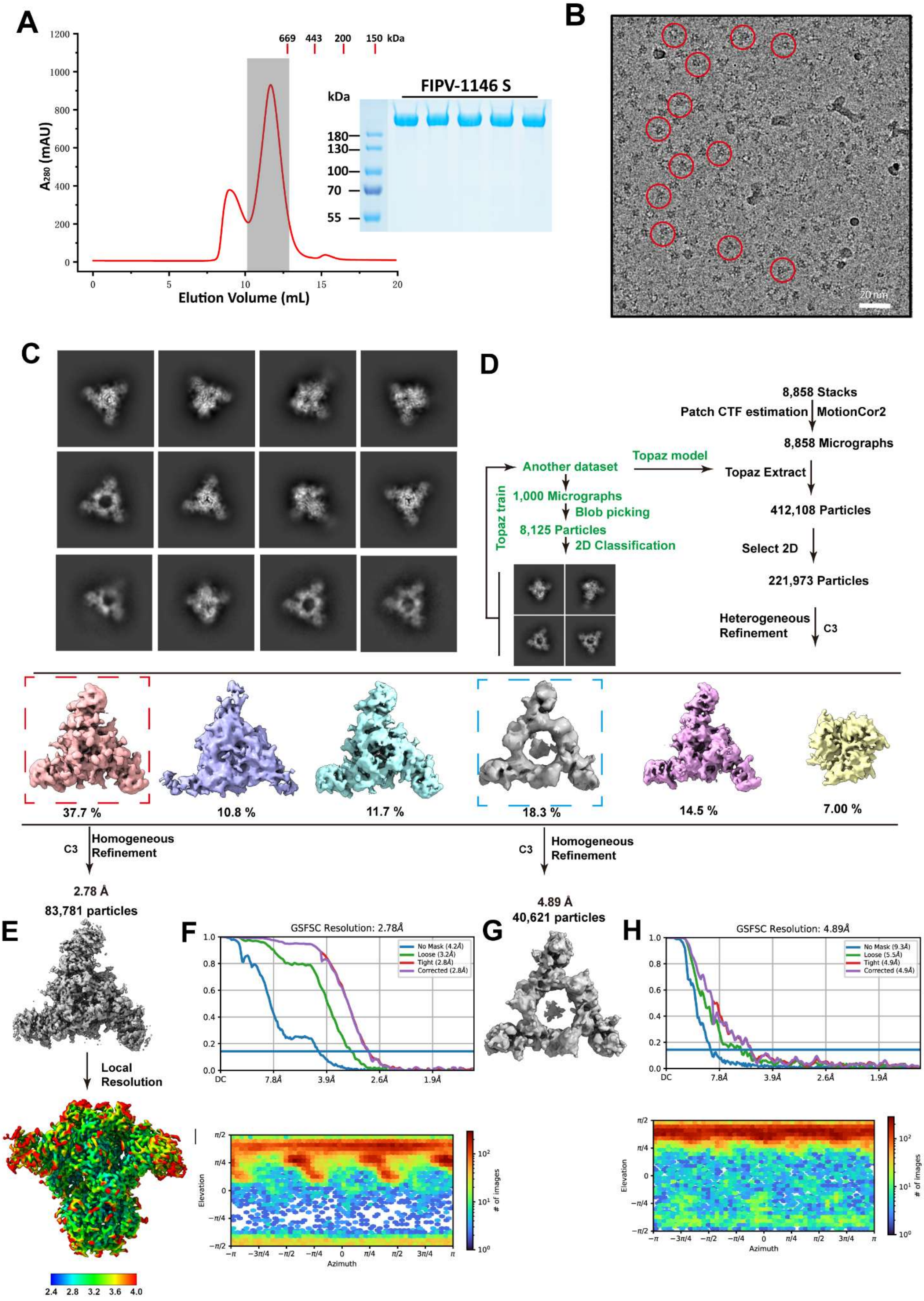

A
B
C
D
E
F
G
H
669 443 200 150 kDa
FIPV-1146 S
kDa
180
130
100
70
55
A280 (mAU)
Elution Volume (mL)
20 nm
8,858 Stacks
Patch CTF estimation
MotionCor2
8,858 Micrographs
Topaz model
Another dataset
Topaz Extract
1,000 Micrographs
Blob picking
Topaz train
8,125 Particles
2D Classification
412,108 Particles
Select 2D
221,973 Particles
Heterogeneous Refinement
C3
37.7 %
10.8 %
11.7 %
18.3 %
14.5 %
7.00 %
Homogeneous Refinement
2.78 Å
83,781 particles
4.89 Å
40,621 particles
Local Resolution
GSFSC Resolution: 2.78Å
No Mask (4.2Å)
Loose (3.2Å)
Tight (2.8Å)
Corrected (2.8Å)
GSFSC Resolution: 4.89Å
No Mask (9.3Å)
Loose (5.5Å)
Tight (4.9Å)
Corrected (4.9Å)
Elevation
Azimuth
# of images

Appendix Figure S1. EM data processing of FIPV-1146 S trimer.

(A) Size-exclusion chromatograms of FIPV-1146 S. The collected elution volume range was annotated by grey color. The individual fractions were further analyzed by SDS-PAGE (right panel). **(B)** Representative cryo-EM micrograph. **(C)** 2D class average images of FIPV-1146 S trimer. **(D)** Flow chart of data processing and reconstruction. **(E)** Map of the reconstruction of the FIPV-1146 S ectodomain trimer colored according to local resolution determined using cryoSPARC. **(F)** Fourier shell correlation (FSC) curve for the FIPV-1146 S trimer reconstruction, and the angular distribution plot of all the particles that contributed to the map. **(G)** Map of the reconstruction of the disassociated FIPV-1146 S1 trimer. **(H)** Fourier shell correlation (FSC) curve for the disassociated FIPV-1146 S1 trimer reconstruction, and the angular distribution plot of all the particles that contributed to the map.

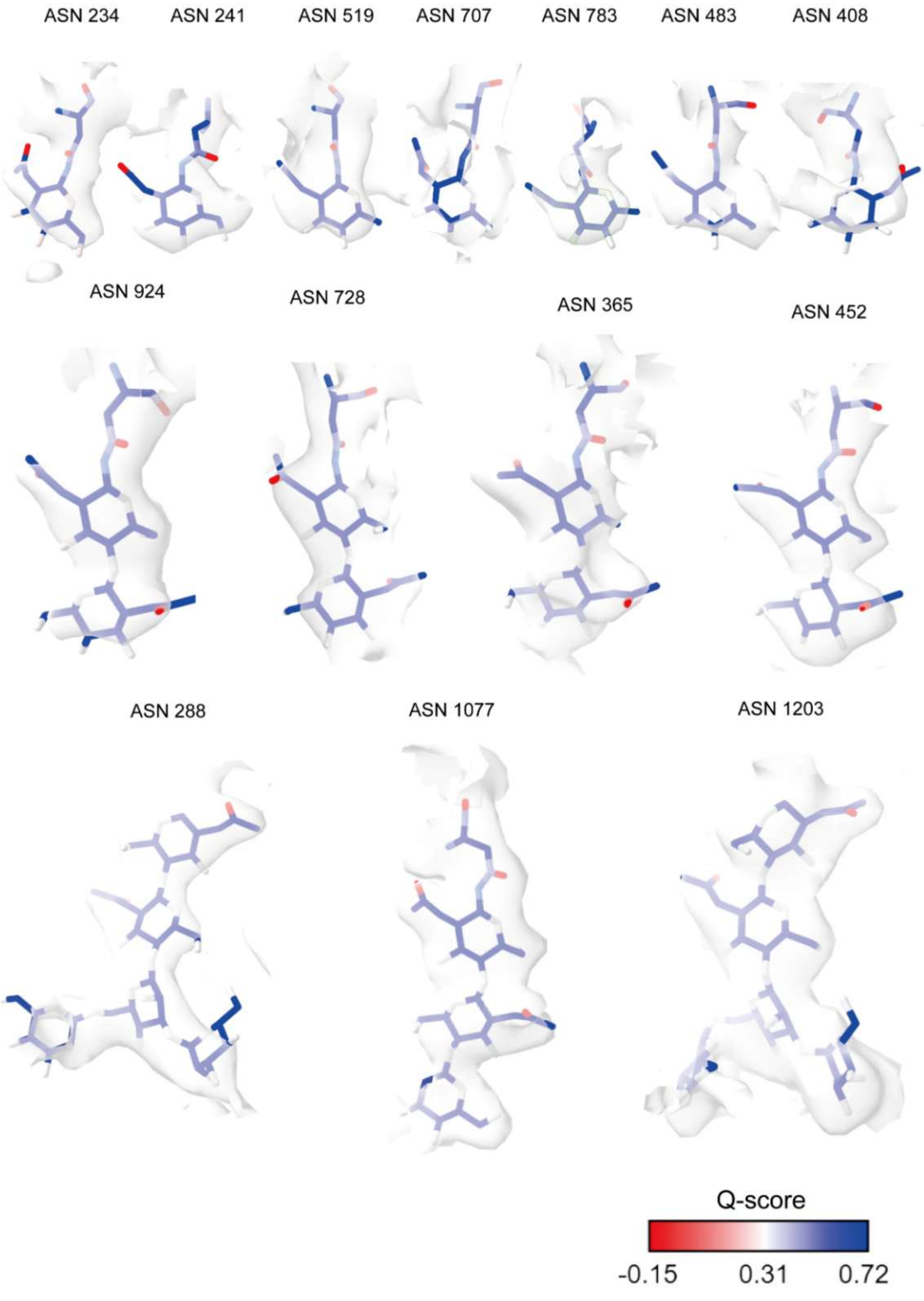

Appendix Figure S2. Cryo-EM densities of representative glycans on FIPV-1146 S and their modeled atomic coordinates.

These 14 glycans were identified by both Cryo-EM and MS, and their Q-scores were calculated as previously described (Pintilie, Zhang et al., 2020).

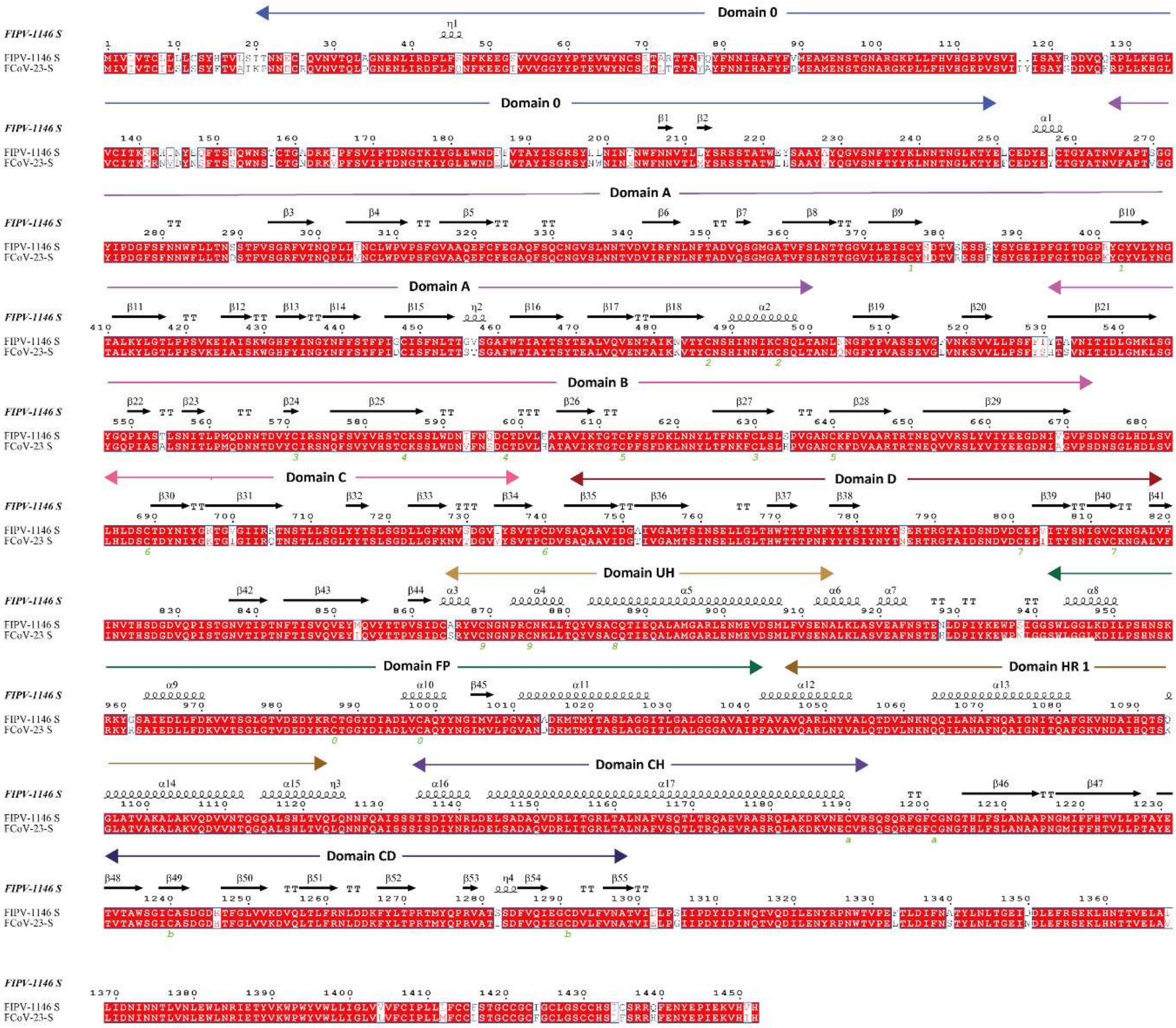

Appendix Figure S3. Sequence alignment of the FIPV-1146 and FCoV-23 S used in this study.

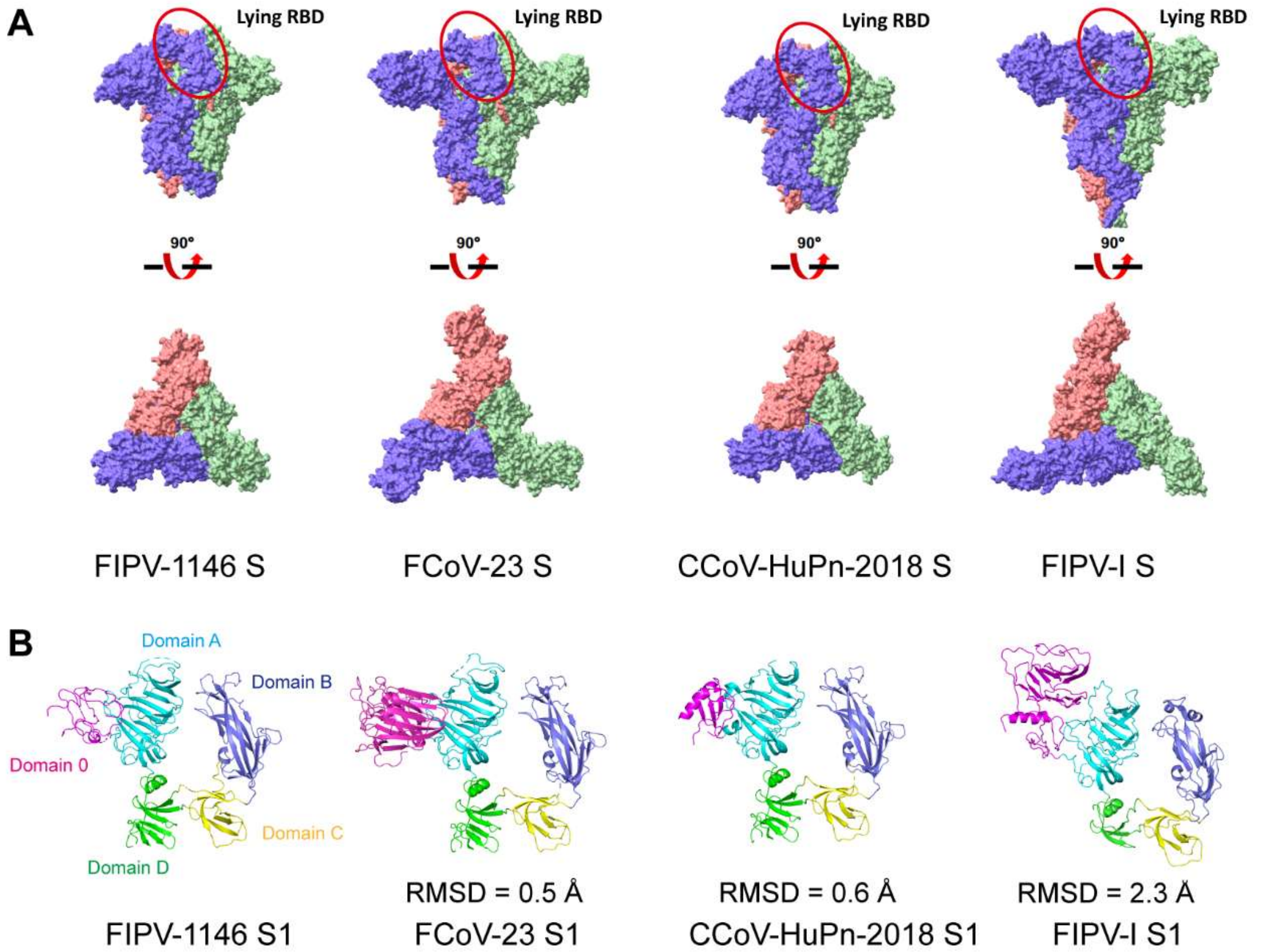

C

	S	S1	S2	D0	DA	DB
**FCoV-23**	93.32%	90.95%	96.26%	83.84%	95.32%	93.66%
**CCoV-HuPn-2018**	82.02%	73.72%	94.43%	31.31%	86.88%	93.75%
**FIPV-I**	45.93%	34.42%	62.06%	23.85%	38.71%	26.19%

Appendix Figure S4. Multiple alignments of FIPV-1146 S and the relevant structures.

(A) Surface representations of the structures of FIPV-1146 S (PDB entry 9UE3), FCoV-23 S (PDB entry 9DB3), CCoV-HuPn-2018 S (PDB entry 7USA) and FIPV-I S (PDB entry 6JX7) are colored by chain. **(B)** Alignment between the FIPV-1146 S1 and the relevant S1 structures. The cartoon representations are colored by individual domain. **(C)** Amino acid identity of S, S1, S2, Domain (D) 0, DA, and DB between FIPV-1146 and the reference strains.

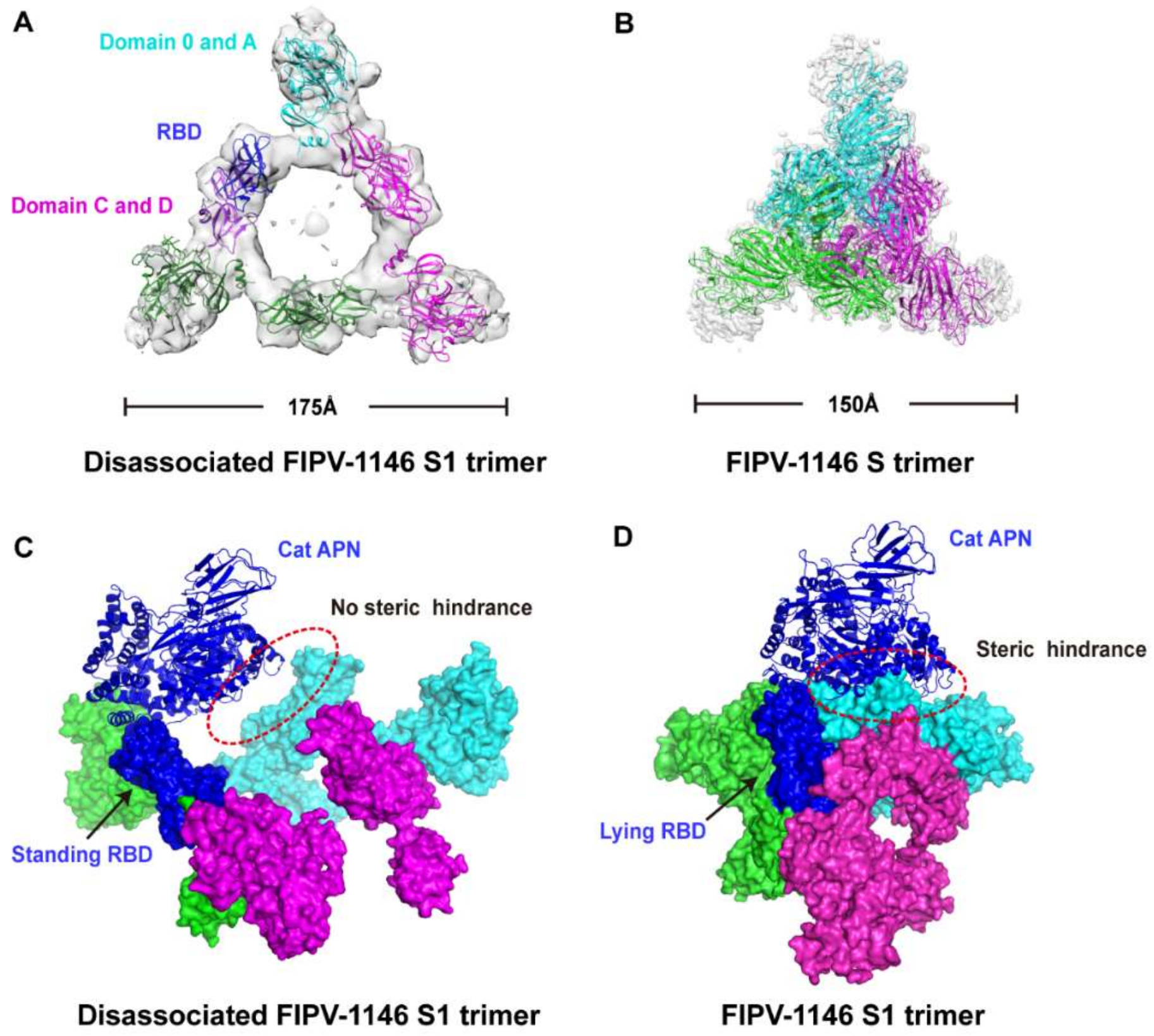

Appendix Figure S5. Low resolution cryo-EM structure of the disassociated FIPV-1146 S1 trimer.

(A) Cryo-EM electron density of the disassociated FIPV-1146 S1 trimer. Individual domains (domain 0, A, B, C, and D) were independently fitted into the density map, excluding inter-domain loop regions. **(B)** Cryo-EM electron density of FIPV-1146 S trimer. The cartoon representation was colored by chain overlaid with the light-gray silhouette of the cryo-EM map. **(C)** Superimposition of FIPV-1146 RBD bound to cAPN onto the proposed disassociated S1 trimer. **(D)** Superimposition of the FIPV-1146 RBD bound to cAPN onto the S1 subunit within the FIPV-1146 S trimer. The structure of FIPV-1146 RBD bound to cAPN colored as blue.

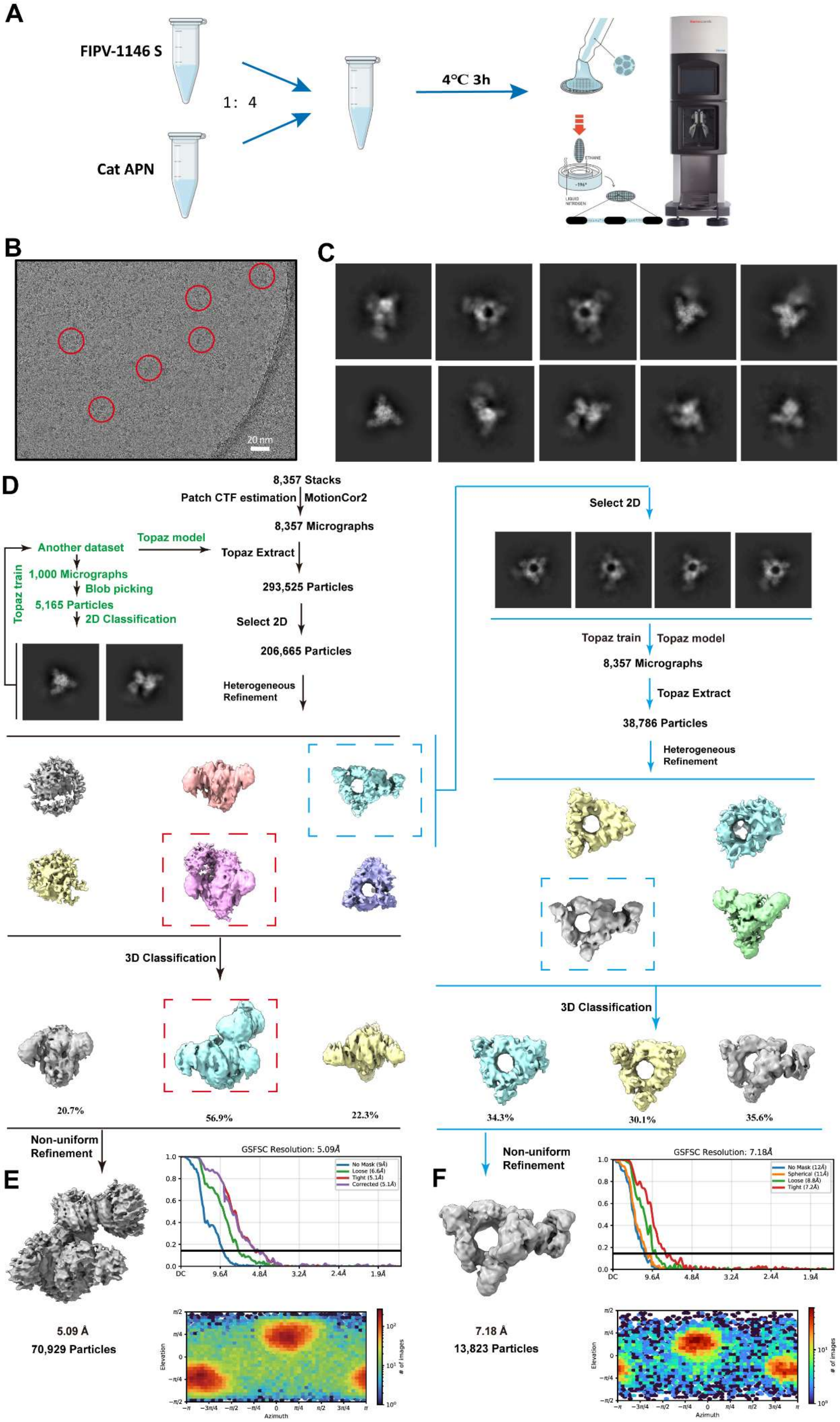
A
FIPV-1146 S
Cat APN
1: 4
4°C 3h
B
20 nm
C
D
8,357 Stacks
Patch CTF estimation
MotionCor2
8,357 Micrographs
Another dataset
Topaz model
Topaz Extract
1,000 Micrographs
Blob picking
5,165 Particles
2D Classification
Topaz train
293,525 Particles
Select 2D
206,665 Particles
Heterogeneous Refinement
3D Classification
20.7%
56.9%
22.3%
Non-uniform Refinement
Select 2D
Topaz train
Topaz model
8,357 Micrographs
Topaz Extract
38,786 Particles
Heterogeneous Refinement
3D Classification
34.3%
30.1%
35.6%
Non-uniform Refinement
E
5.09 Å
70,929 Particles
GSFSC Resolution: 5.09Å
No Mask (9Å)
Loose (6.6Å)
Tight (5.1Å)
Corrected (5.1Å)
F
7.18 Å
13,823 Particles
GSFSC Resolution: 7.18Å
No Mask (12Å)
Spherical (11Å)
Loose (8.8Å)
Tight (7.2Å)
Elevation
Azimuth
# of images

Appendix Figure S6. EM data processing of the complex comprising one FIPV-1146 S trimer bound to cAPN dimer.

(A) The mixture of purified FIPV-1146 S and cAPN was used for cryo-EM grid preparation. **(B)** Representative cryo-EM micrograph. **(C)** 2D class average images of FIPV-1146 S trimer and cAPN complex. **(D)** Flow chart of data processing and reconstruction. **(E)** Map of the reconstructed complex comprising one FIPV-1146 S trimer bound to cAPN dimer. The FSC curve for the reconstructed complex is shown, along with the angular distribution plot of all particles used in the reconstruction. **(F)** Map of the reconstructed complex comprising one disassociated FIPV-1146 S1 trimer bound to one cAPN dimer. The FSC curve for the reconstructed complex is shown, along with the angular distribution plot of all particles used in the reconstruction.

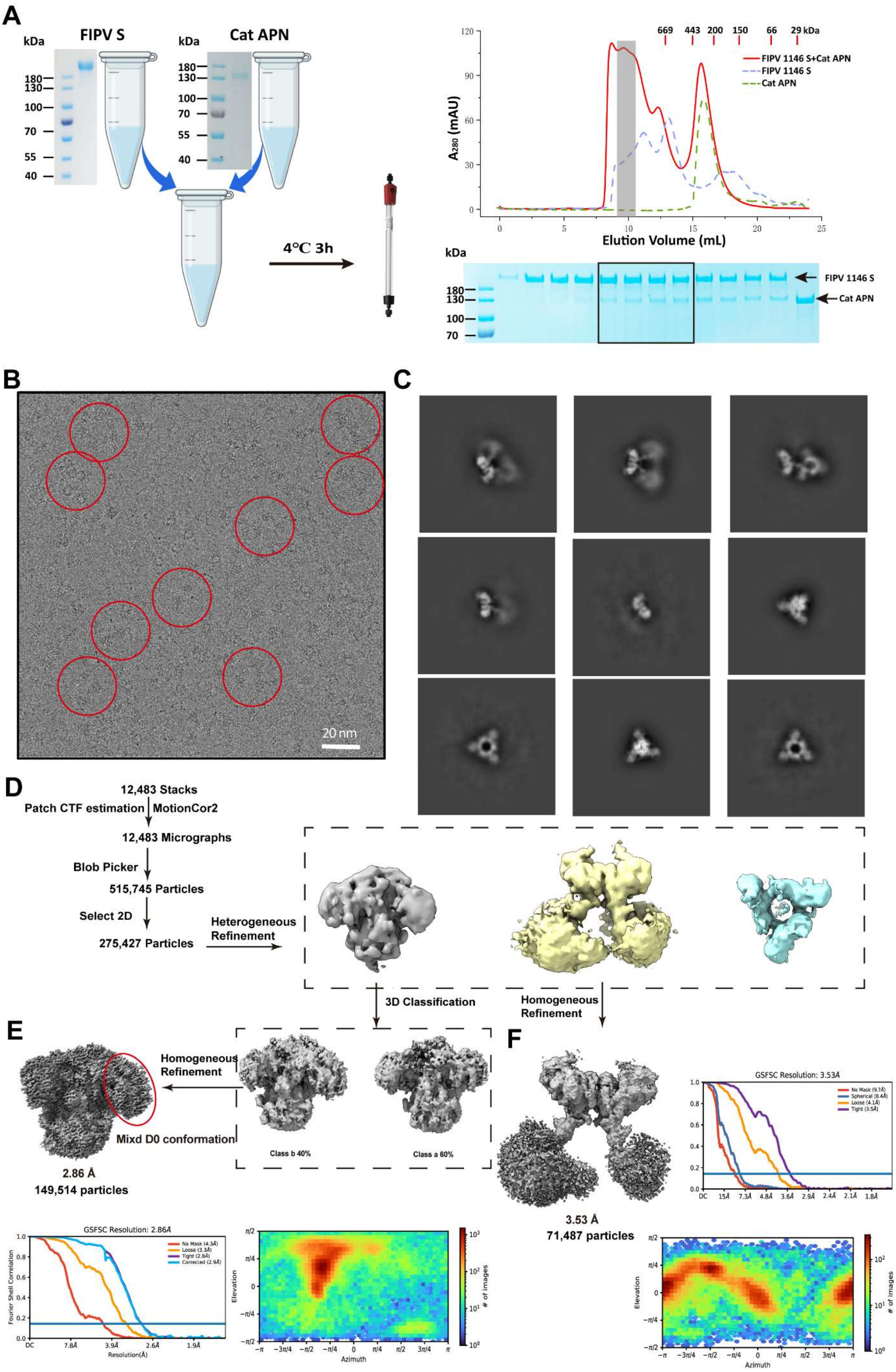
A
FIPV S
Cat APN
kDa
4°C 3h
Elution Volume (mL)
A280 (mAU)
FIPV 1146 S+Cat APN
FIPV 1146 S
Cat APN
669 443 200 150 66 29 kDa
FIPV 1146 S
Cat APN
B
20 nm
C
D
12,483 Stacks
Patch CTF estimation
MotionCor2
12,483 Micrographs
Blob Picker
515,745 Particles
Select 2D
275,427 Particles
Heterogeneous Refinement
3D Classification
Homogeneous Refinement
E
Homogeneous Refinement
Mixd D0 conformation
2.86 Å
149,514 particles
Class b 40%
Class a 60%
F
3.53 Å
71,487 particles
GSFSC Resolution: 3.53Å
GSFSC Resolution: 2.86Å
Fourier Shell Correlation
Resolution(Å)
Elevation
Azimuth
# of images

Appendix Figure S7. EM data processing of the complex comprising two FIPV-1146 S trimer bound to cAPN dimer.

(A) The mixture of purified FIPV-1146 S and cAPN was injected into Superose 6 increase 10/300 GL column to identify the putative complex fractions (indicated by the light grey background in the plot), which were later collected for cryo-EM grid preparation. For comparison, the SEC profiles of both FIPV-1146 S (blue) and cAPN ectodomain (green) were aligned onto the SEC profile of the mixture (red) to demonstrate the substantial shift in the elution volume. **(B)** Representative cryo-EM micrograph. **(C)** 2D class average images of the complex comprising two FIPV-1146 S trimer bound to one cAPN dimer. **(D)** Flow chart of data processing and reconstruction. **(E)** Map of the reconstructed FIPV-1146 S trimer with mixed D0 conformations. The FSC curve is shown, along with the angular distribution plot of all particles used in the reconstruction. **(F)** Map of the reconstructed complex comprising two FIPV-1146 S trimer bound to one cAPN dimer. The FSC curve of the reconstructed complex is shown, along with the angular distribution plot of all particles used in the reconstruction.

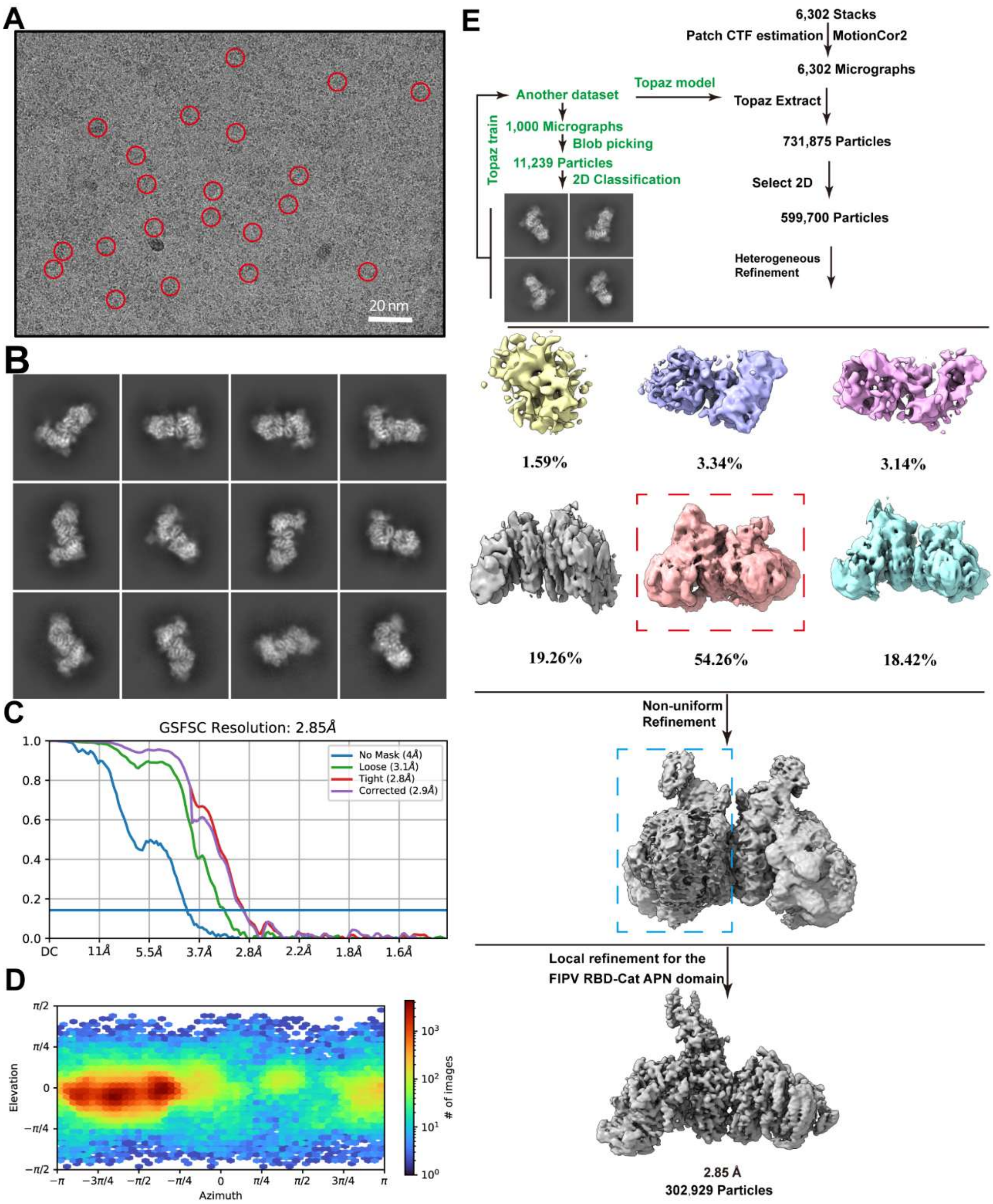

Appendix Figure S8. EM data processing of FIPV-1146 RBD-cAPN complex.

(A) Representative cryo-EM micrograph. **(B)** 2D class average images of FIPV-1146 RBD-cAPN complex. **(C)** The FSC curve of final EM map and model vs. map. **(D)** Angular distribution of the particles for 3D reconstruction. **(E)** Flow chart of data processing and reconstruction.

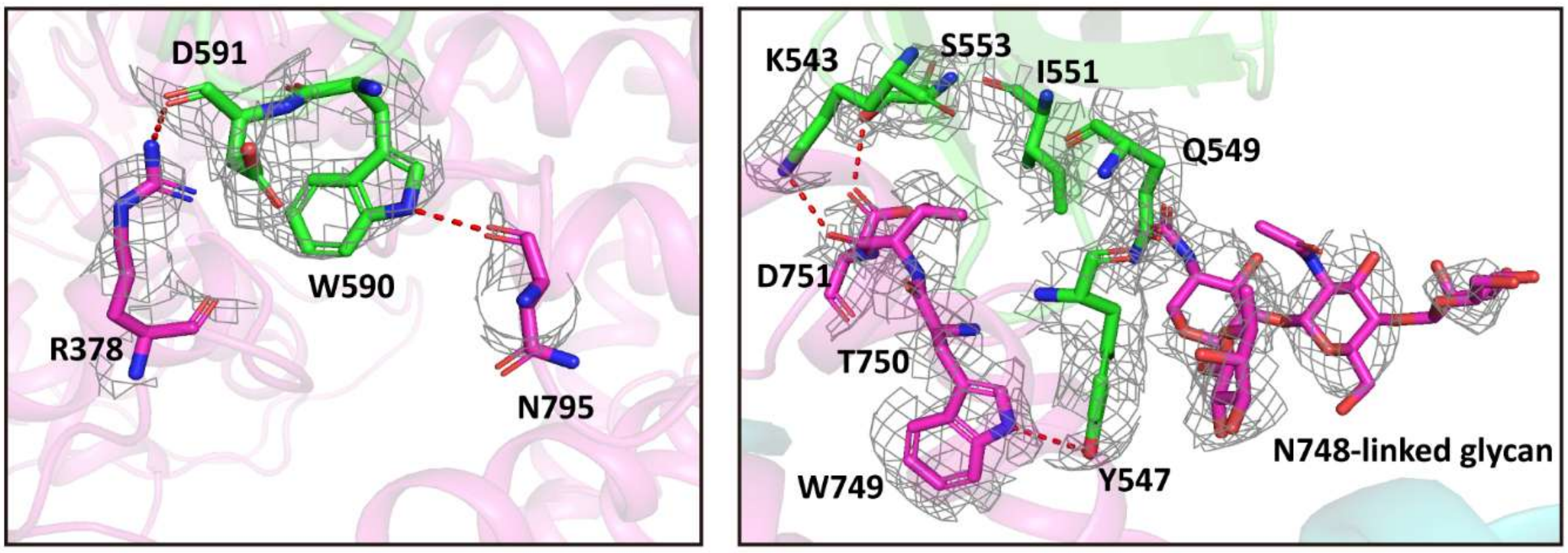

Appendix Figure S9. Representative electron density maps at the binding interface, related to Figure 4

The electron densities of residues at the interaction interface between FIPV-1146 RBD and cAPN. The density maps are drawn in gray mesh contoured at 0.5 σ. The FIPV-1146 RBD is colored green. cAPN is marked in magenta.

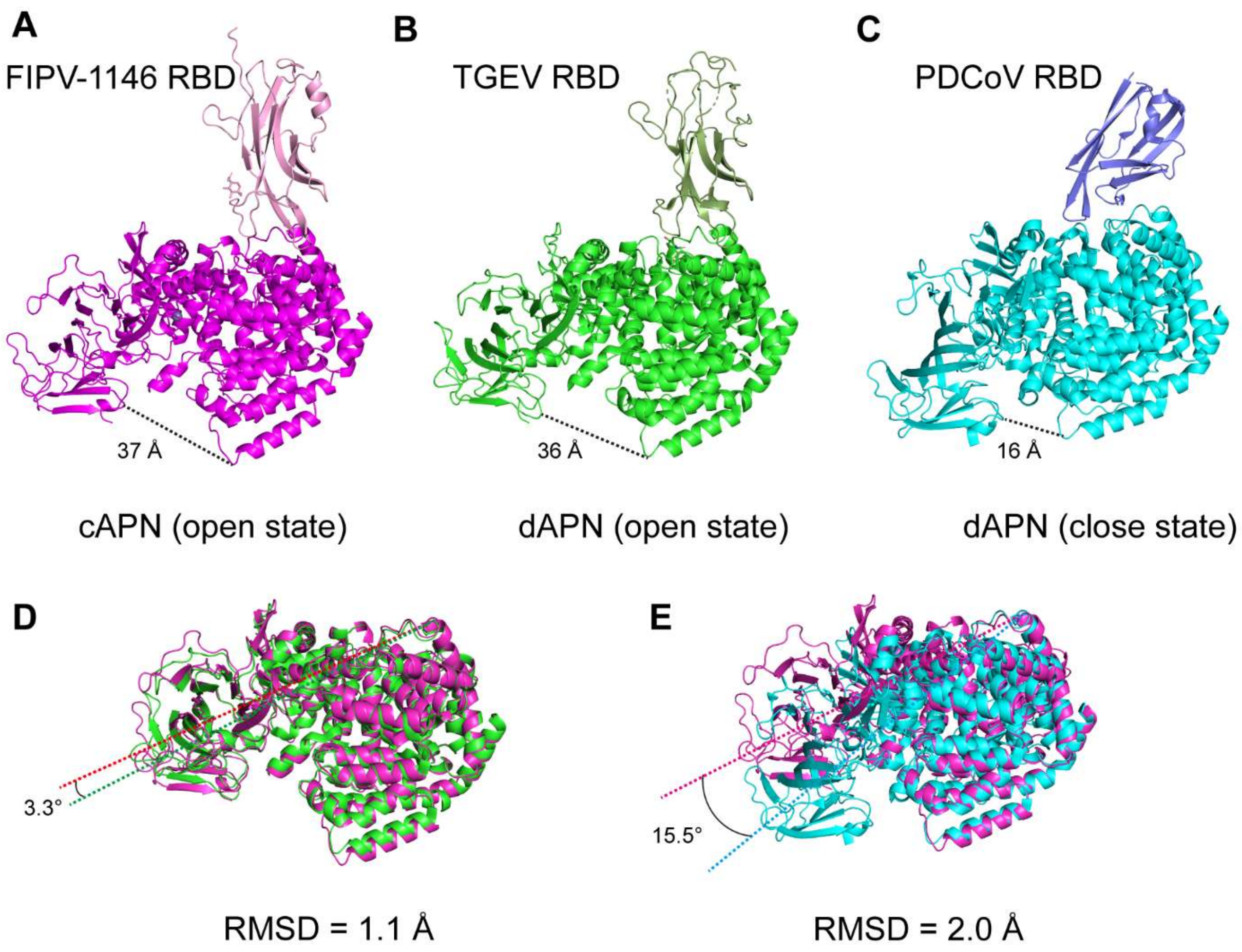

Appendix Figure S10. Overall structure of APNs in complex with FIPV-1146, TGEV and PDCoV RBDs.

(A) The complex structure of FIPV-1146 RBD bound to cAPN. **(B)** The complex structure of TGEV RBD bound to dAPN (PDB entry 8Z27). **(C)** The complex structure of PDCoV RBD bound to dAPN (PDB entry 8YZI). **(D)** The cAPN monomer of the FIPV-1146 RBD-cAPN complex is superimposed with the dAPN monomer of TGEV RBD-dAPN complex. **(E)** The cAPN monomer of the FIPV-1146 RBD-cAPN complex is superimposed with the dAPN monomer of PDCoV RBD-dAPN complex to illustrate the difference between the closed and open form as defined in the legend.

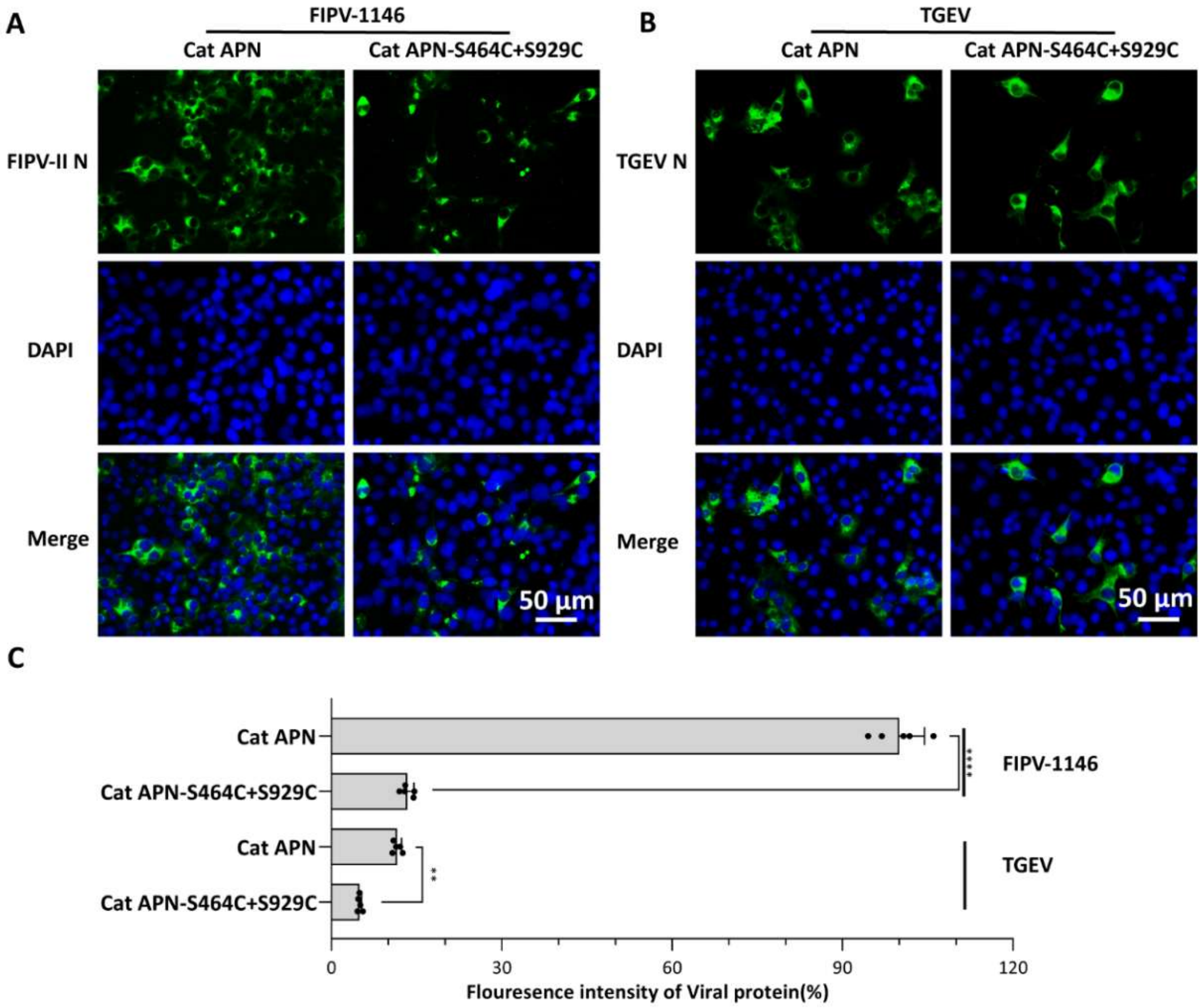

Appendix Figure S11. Mutational analysis of S464C+S929C in cAPN involved in the susceptibility of FIPV-1146 and TGEV.

(A) FIPV-1146 infection in BHK-21 cells overexpressing the cAPN or its mutant S464C+S929C. Green fluorescence indicates BHK-21-APN cells infected with FIPV-1146. The scale bar indicates 50 μm. **(B)** TGEV infection in BHK-21 cells overexpressing the cAPN or its mutant S464C+S929C. Green fluorescence indicates BHK-21-APN cells infected with TGEV. The scale bar indicates 50 μm. **(C)** The fluorescence intensity in (A) and (B) was determined by software Image J. For the FIPV-1146-challenged group, Cat APN vs Cat APN-S464C+S929C, $P = 2.30\times10^{-14}$, For the TGEV-challenged group, Cat APN vs Cat APN-S464C+S929C, $P = 2.06\times10^{-03}$. Data are expressed as mean ± SD, n = 5. Error bars denote standard deviations for the samples.

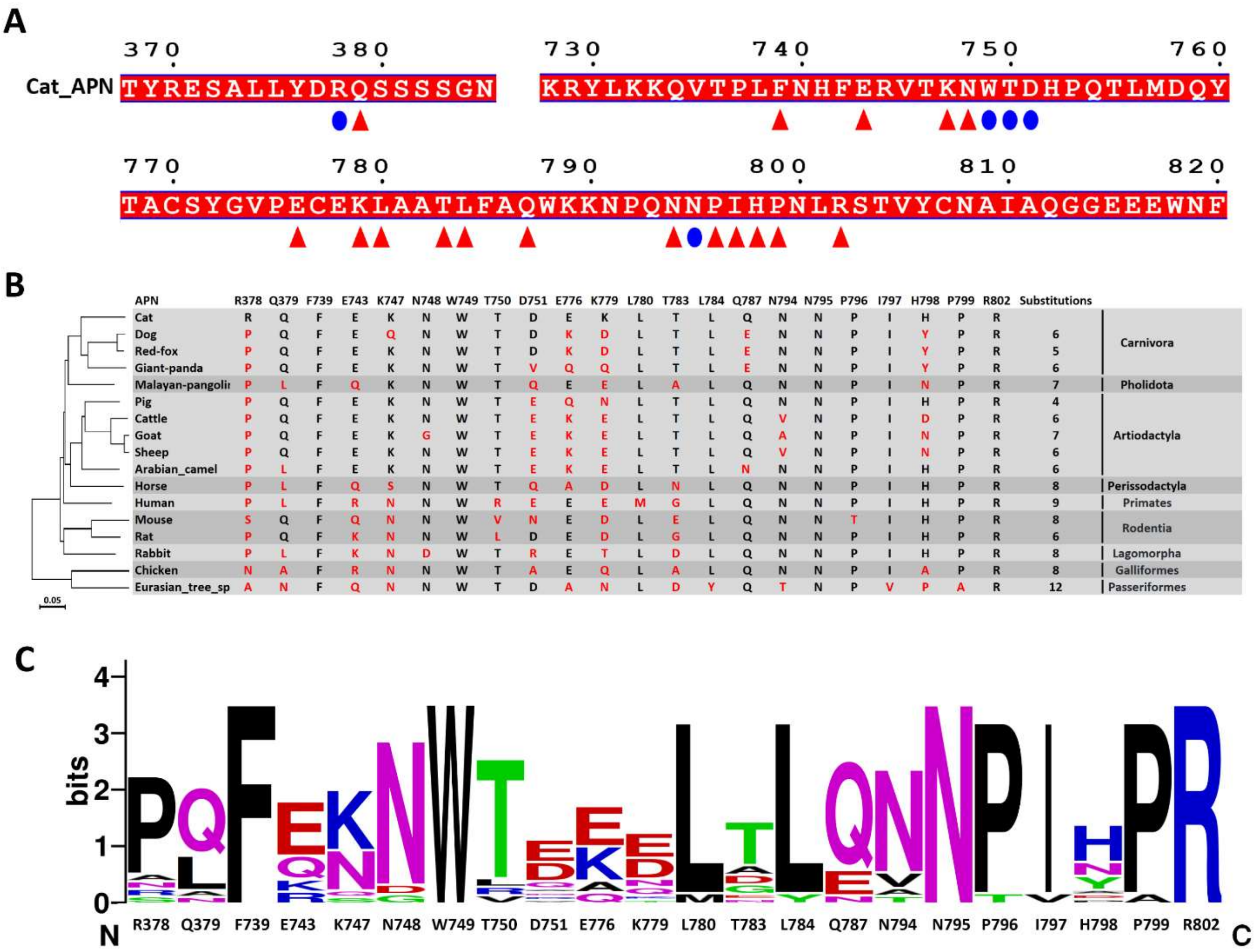

Appendix Figure S12. Sequence alignment of 17 animal APNs, based on 22 APN residues involved in FIPV-1146 RBD binding.

(A) The cAPN sequence is depicted, with residues involved in H-bond with the FIPV-1146 RBD marked by blue circles, and other binding residues marked by red triangles. **(B)** 22 residues of cAPN which are crucial in interacting with the FIPV-1146 RBD are listed, respectively. Red letters indicate the substitutions in the APN of 16 animal species. The 17 species belonging to nine orders are shown in the right column. Phylogenetic tree based on APN amino acid sequences was generated using MEGA X. **(C)** Sequence conservation analysis of 22 residue positions across 17 APNs, visualized by WebLogo.

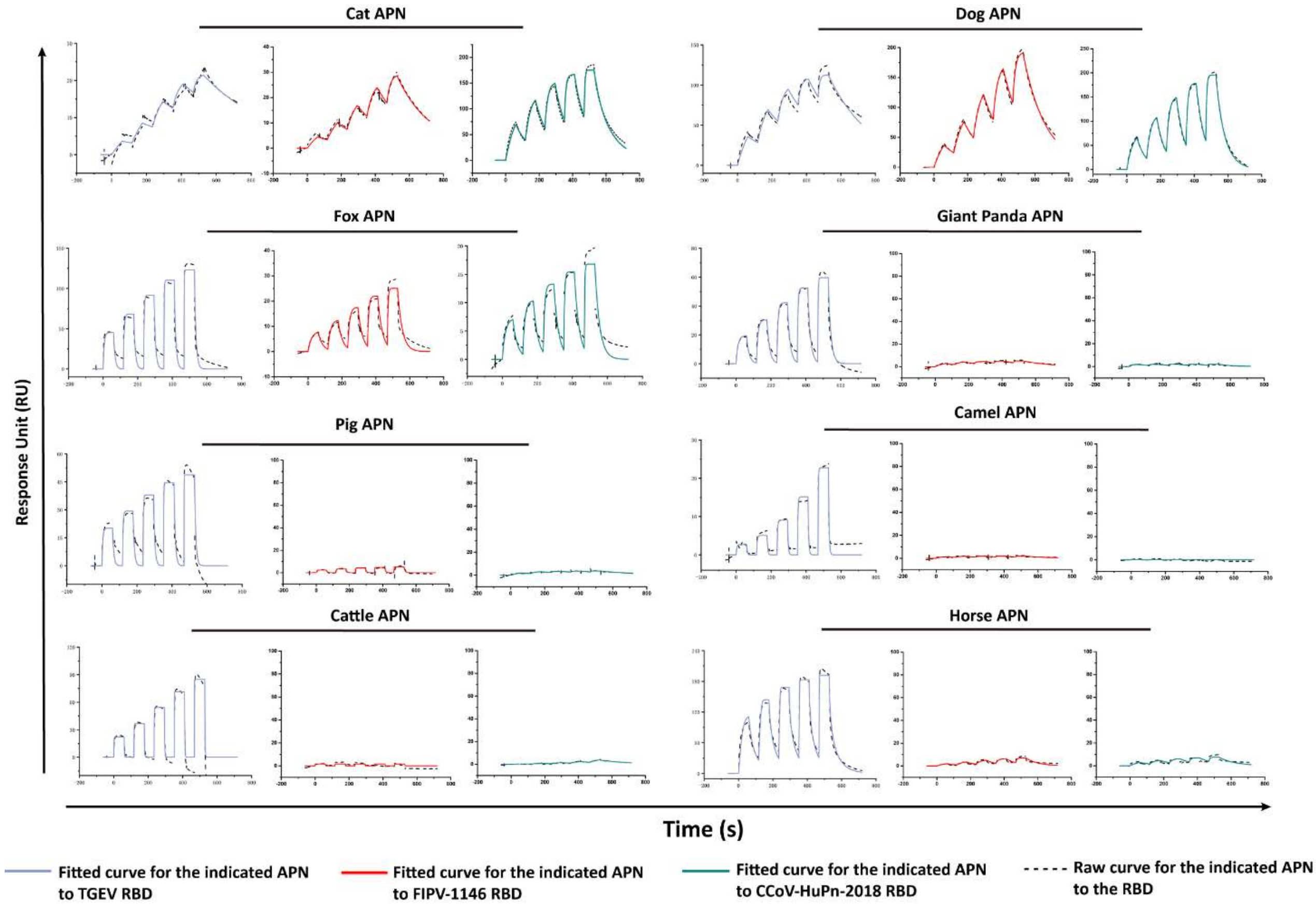

Appendix Figure S13. Binding curves of eight APNs to the RBDs from TGEV, FIPV-1146 and CCoV-HuPn-2018, related to Figure 5B.

The hFc-tagged APNs were captured by protein A chip at more than 1000 response units, and sequentially tested the binding with serially diluted the RBDs of TGEV, FIPV-1146 and CCoV-HuPn-2018. The raw and fitted curves are shown as dotted and solid lines, respectively. One representative data is shown of three independent experiments.

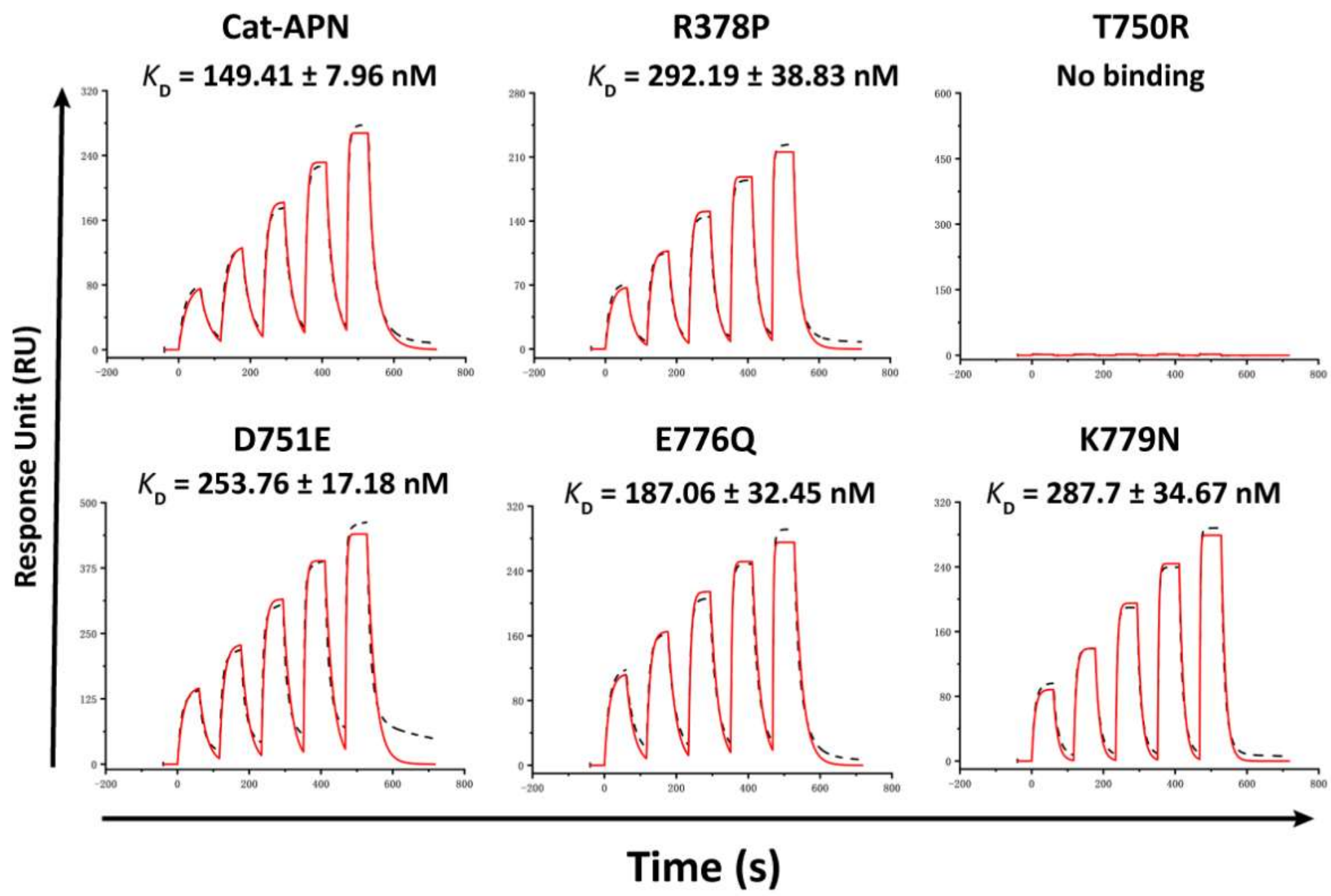

Appendix Figure S14. Binding affinity assay between the cAPN mutants and FIPV-1146 RBD by SPR.

The hFc-tagged cAPN mutants were captured by protein A chip at more than 1000 response units, and sequentially tested the binding with serially diluted FIPV-1146 RBD. The binding affinities of the APN mutants to the FIPV-1146 RBD are shown. Mean ± SD represents the mean and standard deviation of three independent experiments. The raw and fitted curves are shown as dotted and solid lines, respectively. One representative data is shown of three independent experiments.

Appendix Table S1. Cryo-EM data collection, refinement and validation statistics

	FIPV-1146 RBD-cAPN	FIPV-1146 S	One FIPV-1146 S-cAPN	FIPV-1146 S with mixed D0 conformation	FIPV-1146 S1 trimer-cAPN	Two FIPV-1146 S-cAPN	FIPV-1146 S1 trimer
EMBD	EMD-64088	EMD-64076	EMD-64643	EMD-67940	EMD-67258	EMD-67259	EMD-67260
PDB	9UEP	9UE3					
**Data collection and processing**							
Magnification	105k	105k	81k	105K	81K	105K	105K
Voltage (kV)	300	300	300	300	300	300	300
Electron exposure (e–/Å^2)	60	50	50	50	50	50	50
Defocus range (μm)	-1.0 ~ -2.0	-1.0 ~ -2.0	-1.0 ~ -2.0	-1.0 ~ -2.0	-1.0 ~ -2.0	-1.0 ~ -2.0	-1.0 ~ -2.0
Pixel size (Å)	0.69	0.81	0.86	0.81	0.81	0.81	0.81
Symmetry imposed	C1	C3	C1	C1	C1	C1	C1
Initial particle images (no.)	731,875	412,108	206,665	275,427	38,786	275,427	412,108
Final particle images (no.)	302,929	83,781	70,929	149,514	13,823	71,487	40,621
Map resolution (Å)	2.85	2.78	5.09	2.86	7.18	3.53	4.89
FSC threshold	0.143	0.143	0.143	0.143	0.143	0.143	0.143
**Refinement**							
Initial model used (PDB code)	8Z27	7USA					
Model resolution range (Å)	Up to 2.85	Up to 2.78					
Map sharpening *B* factor (Å^2)	DeepEMhancer (no B factor value)	DeepEMhancer (no B factor value)					
Model composition							
Non-hydrogen atoms	8515	8943					
Protein residues	1049	1094					
Ligands	13	38					
B factors (Å^2)							
Protein	174.84	50.00					
Ligand	50.00	50.00					
R.m.s. deviations							
Bond lengths (Å)	0.004	0.006					
Bond angles (°)	0.684	0.987					
Validation							
MolProbity score	2.04	1.78					
Clashscore	8.90	4.22					
Poor rotamers (%)	0.00	0.70					
Ramachandran plot							
Favored (%)	96.17%	94.06%					
Allowed (%)	3.83%	5.66%					
Disallowed (%)	0.00%	0.28%					

Appendix Table S2. A summary of N-glycosylation sites of FIPV-1146 S observed experimentally through mass spectrometry and cryo-EM.

N-sites	Detected by cryo-EM [a]	MS		
		Detected? [a]	Percentage of modified site (%) [b]	Number of *N*-glycan structures detected [c]
29	×	○	100.0	49
67	×	○	99.8	2
95	×	○	100.0	4
155	×	○	100.0	60
174	×	○	99.7	25
208	×	○	98.4	52
234	○	○	67.0	65
241	○	○	100.0	55
288	○	○	100.0	14
337	○	○	100.0	36
348	○	○	98.8	24
365	○	○	100.0	49
408	○	○	99.0	42
452	○	○	99.9	85
483	○	○	98.8	22
519	○	○	100.0	45
535	○	○	100.0	47
557	○	○	100.0	32
565	○	○	N/A [d]	N/A [d]
707	○	○	100.0	29
728	○	○	90.0	18
783	○	○	99.6	54
822	○	○	100.0	5
837	○	○	100.0	16
843	○	○	100.0	50
924	○	○	100.0	34
1077	○	○	100.0	12
1203	○	○	100.0	10
1297	○	○	100.0	59
1314	×	○	91.0	24
1327	×	○	28.0	28
1339	×	○	99.4	32
1344	×	○	100.0	41
1361	×	○	100.0	38
1374	×	○	100.0	19

a. Key: ○ - detected, × - not detected. b. The percentages of modified sites (i.e., glycosylated) were calculated from the detected glycopeptides (deamidated N with +2.988261) and nonglycosylated peptides containing respective *N*-sites in PNGase F-treated experiments. c. The

number of unique glycan masses were calculated based on the detected glycopeptides in all measurements with five different protases in the absence or presence of IMPa or PNGase F at two instrumental conditions (HCDpdEThCD and sceHCD). d. N/A = not applicable. Only deamidated peptide associated with N565 was detected.

Appendix Table S3. A summary of O-glycosylation sites of FIPV-1146 S observed experimentally through mass spectrometry.

O-sites	Percentage of modified site (%) [a]
**"O-Follow-N"**	
S156/T157	< 0.1
S233/T236	2.7 ± 1.9
T243/T248	3.6 ± 2.5
T350/S355	0.2
T410	1.7 ± 0.4
T454/T455	1.9 ± 1.6
T458/S489	< 0.1
S780/T785	2.7 ± 2.22
T1079	1.5 ± 0.9
T1299	0.98
T1362/T1363	1.7 ± 2.0
T1376	3.3 ± 2.6
**Other O-sites**	
T62	NA [b]
T263/T269/T270	< 0.1
S378/T380/S382/S384/S368/S388	< 0.1 – 0.7
S574/S578	4.3
T857/T858	0.5 – 11.2
S953/S956	< 0.1 – 1.39
T1092/S1903	0.77

a. The percentages of modified sites (i.e., glycosylated) were calculated using two approaches: (i) from the intensities of *N*-glycopeptides and *N*-and-*O*-glycopeptides at the same N-site, or (ii) from the intensities of *O*-glycopeptides and respective non-*O*-glycopeptides. b. N/A = not applicable due to respective non-*O*-glycopeptide not detected.

Appendix Table S4. The interaction of cat APN with the RBDs of FIPV-1146 and FCoV-23.

Cat APN	FIPV-1146 RBD	FCoV-23 RBD
**R378**	W590 (6), D591 (9,**1**)	W592 (2)
**Q379**	W590 (6), D591 (1)	W592 (9), D593 (2)
**F739**	Y547 (2)	Y549 (2)
**E743**	Y547 (6)	Y549 (10)
**K747**	I551 (4), T554 (2)	I553 (1)
**N748**	S545 (4), G546 (1), Q549 (3), I551 (4)	S547 (7), G548 (3,**1**), Y549 (6), Q551 (5,**1**)
**W749**	S545 (2), G546 (5), Y547 (8,**1**)	G548 (5), Y549 (8,**1**)
**T750**	K543 (7,**1**), S545 (3), I551 (5)	K545 (2), L546 (1), S547 (3), I553 (4)
**D751**	K543 (3), S553 (4,**1**)	
**E776**	Y547 (5)	Y549 (5)
**K779**	Y544 (5)	
**L780**	G546 (5), Y547 (8)	G548 (5), Y549 (7)
**T783**	G546 (5), Y547 (5), G548 (2)	G548 (6), Y549 (4), G550 (2)
**L784**	G546 (4)	G548 (5)
**Q787**	L544 (2)	L546 (5), S547 (3,**1**), G550 (2)
**N794**	L589 (2)	L591 (1)
**N795**	L589 (1), W590 (6,**1**)	L591 (1), W592 (4,**1**)
**P796**	L589 (2), W590 (8)	L591 (1), W592 (6)
**I797**	W590 (6)	W592 (7)
**H798**	W590 (13)	W592 (20)
**P799**	W590 (10)	W592 (10)
**R802**	W590 (3)	W592 (3)
**A1012**	Y547 (3), Q549 (5), I551 (4), N651 (1), Q653 (3)	Y549 (3), Q551 (18), I553 (3), N653 (1), Q655 (2)
**G1013**	Y547 (18), Q549 (3)	Q551 (5), Y549 (19)
**N1014**	Q549 (3)	
**Total**	218, **5**	219, **5**

Numbers in the parentheses beside FIPV-1146 and FCoV-23 RBD residues represent the number of vdw contacts. Numbers in parentheses beside either ligand residues represent the number of vdw contacts. The numbers with underline suggest numbers of potential H-bonds between the pairs of residues. vdw contact was analyzed at a cutoff of 4.5 Å and H-bonds at a cutoff of 3.5 Å.

References

Pintilie G, Zhang K, Su Z, Li S, Schmid MF, Chiu W (2020) Measurement of atom resolvability in cryo-EM maps with Q-scores. *Nat Methods* 17: 328-334
