# Supplementary material for: Structure and receptor recognition of type-II feline infectious peritonitis virus spike glycoprotein
Source: EMBO J. 2026 Jul 8;45(15):5537–55. doi: 10.1038/s44318-026-00849-2 (PMC13434051; doi:10.1038/s44318-026-00849-2)
Supplement: Supplementary file 2 [file 44318_2026_849_MOESM2_ESM.pdf]

THE EMBO JOURNAL

Structure and receptor recognition of type-II feline infectious peritonitis virus spike glycoprotein

Sheng Niu, Yuyang Tian, Linh Nguyen, Chongzhi Bai, Xiaohan Hou, Dan Wang, Ziqian Niu, Zhimin Liu, Jianle Ren, Wentao Li, Qihui Wang, Wen-xia Tian, Junqing Sun, and George Gao

Corresponding authors: Sheng Niu (niusheng@sxau.edu.cn) , Wen-xia Tian (tianwx@sxau.edu.cn), Junqing Sun (sunjunqing@zju.edu.cn)

Review Timeline:

Submission Date:	11th Jul 25
Editorial Decision:	19th Aug 25
Appeal Received:	11th Jan 26
Editorial Decision:	20th Feb 26
Revision Received:	4th May 26
Editorial Decision:	9th Jun 26
Revision Received:	12th Jun 26
Accepted:	24th Jun 26

Editor: Ieva Gailite

Transaction Report:

(Note: With the exception of the correction of typographical or spelling errors that could be a source of ambiguity, letters and reports are not edited. Depending on transfer agreements, referee reports obtained elsewhere may or may not be included in this compilation. Referee reports are anonymous unless the Referee chooses to sign their reports.)

1st Editorial Decision 19th Aug 2025

Dear Dr. Sun,

Thank you for submitting your manuscript for consideration by The EMBO Journal. We have now received comments from two reviewers, which are included below for your information. Based on these comments, we unfortunately had to conclude that the study is not a sufficiently strong candidate for publication in The EMBO Journal.

As you can see, while the reviewers find the study of potential interest and reviewer #2 is more positive in their assessment, both reviewers indicate that the currently provided structural data insufficiently support the proposed opening of the FIPV-II spike protein upon an interaction with its receptor. Since these concerns affect the main conclusions of the study and major experimental revision with an uncertain outcome would be needed to address them, I am afraid that we cannot offer to invite a revised manuscript.

Nevertheless, if you find that you can improve the structural data sufficiently to address the points raised by the reviewers, I would be willing to reconsider the revised manuscript. In this case, it means that I would send it back to the same reviewers, if possible, but would allow them to make new comments on the data, which might then have to be further addressed if the reviewers are more positive in this round of assessment.

Thank you in any case for the opportunity to consider this manuscript. I am sorry that I could not communicate more positive news, but I nevertheless hope that you will find our reviewers' comments helpful for further improvement of the manuscript.

With kind regards,

Ieva Gailite

Ieva Gailite, PhD
Senior Scientific Editor
The EMBO Journal
Meyerhofstrasse 1
D-69117 Heidelberg
Tel: +4962218891309
i.gailite@embojournal.org

____

Referee #1:

EMBO type II FIPV report

Niu, et al. described the use of cryo-electron microscopy (cryo-EM) to determine the structures of the serotype II FIPV spike (S) protein in both apo form and in complex with its receptor. In addition, they provided a detailed analysis of the interaction between feline APN and the receptor-binding domain (RBD) of the FIPV-II S protein. The study also explored the molecular basis for cross-species infection by examining 17 APN orthologs, and it unambiguously demonstrated the importance of the N748-linked glycan and surrounding charged residues in receptor recognition. However, several concerns need to be addressed to strengthen the functional interpretation, data presentation, and reproducibility of the study. Specifically, the cryo-EM data quality requires further improvement to generate sufficient confidence to the model building and functional annotation. The current presentations appear too preliminary in terms of the structural analyses and presentation for the publication in EMBO Journal. Specific comments are listed below.

Major comments:

1. As it currently stands, the cryo-EM maps do not have sufficient resolution and quality to support the descriptions of detailed molecular interactions (Figures 1-3 and more critically Figure 6). The authors need to provide clear structure-to-map superposition of the key contacts to demonstrate the map quality that supports the model building. Additionally, the authors are recommended to provide the Q-scores of the selected molecular contacts for a more unbiased quality evaluation.
2. P8 The authors mentioned that "The 22 key residues in cAPN responsible for binding to the FIPV-II RBD were highlighted and compared with 16 APN orthologs based on the complex structures in this study." But there is no clear cross-referencing in the figure about these key residues. Presumably, the molecular details of the RBD:APN interactions are presented in Figure 6, but

the number of residues illustrated in Figure 6 does not match the description on P8.
3. From the angular distributions of the observed particle images (Supplementary Fig. S4D and S5D), it is apparent that some complexes exhibit preferred orientation issues. The authors need to optimize their cryo-EM sample conditions to improve the preferred orientation issues.
4. The authors reported the cryo-EM map and rigid-body docking modeling of the dissociated S1 trimer (Figure 2), but it is unclear whether this structure is simply an experimental artifact or of functional relevance. The biological implication of the dissociated S1 trimer is not mentioned in the Discussion section, which should be appropriately addressed.
5. It is stated that the cryo-EM map of the dissociated S1 trimer was resolved to 4.8 Å (Fig. 2) but not FSC curve was shown in Supplementary Fig. 4. Importantly, the map presented in Fig. 2A does not meet the expectation of such a resolution - beta-sheet separation and well-defined helix should be expected. The authors need to provide sufficient evidence to ensure that the descriptions of the structural quality match the actual experimental results. A minor point about the statement on P6 "However, disassociated FIPV-II S1 trimer particles ... at 4.8 Å (Fig. S2)." The cross-reference to Fig. S2 should be corrected to Fig. 2A.
6. Through systematic biochemialal and biophysical characterizations of Type II FIPV S protein interactions with the 17 APN orthologs, the authors established several APN's direct binding to the Type II FIPV S protein (Figures 4, 5 and 7). However, the cryo-EM map of the S trimer in complex the APN dimer (Figure 3 and Supplementary Fig. S4) require major improvements to assure sufficient confidence of the mode of binding between RBD and APN.
* The statement that "Additionally, during 2D classification, we identified the category of two S trimers bound to one cAPN dimer (Figs. 3C and D)." (end of P7) does not have strong support in Fig. 3. The selected 2D class images are too ambiguous to support such as statement.
* In addition to the preferred orientation issue, the low map resolution (5Å) is another serious issue for the modeling of the ternary complex.
* There is no sufficient details in the workflow on Supplementary Fig. 6 to ensure the reproducibility of the cryo-EM data processing.
* Importantly, the authors need to provide sufficient details to describe how the atomic model was generated to avoid steric clashes between different functional domains required for the opening of the RBD as presented in Fig. 3.
7. P14. "we observed that a single cAPN dimer could simultaneously bind to two FIPV-II S trimers" The 2D class averages in Fig. 3C are ambiguous. Complementary methods such as mass photometry (MP) or SEC-MALS could help confirm the stoichiometry of this interaction.
8. Accordingly, the authors need to address the issues regarding RBD:APN interactions with clear supporting evidence:
* P10. "............cAPN also adopts an open conformation in the FIPV-II RBD-cAPN complex.............." The authors should provide additional details on the open and closed conformations of APN. Did the cryo-EM data show heterogeneity between these states? Figure S5 suggests otherwise. It should also be clarified whether reference volumes were introduced during the heterogeneous refinement step (Fig. 5E).
* P11. "cAPN formed a hydrogen bond network with amino acids (K524, Y528, S534, W571, and D572) of the FIPV-II RBD (Fig. 6B)." The manuscript should clarify the methodology/software used to define hydrogen bonds. The current figure does not explicitly show these interactions.
* P11. "While pig APN (pAPN) retains the N748-linked glycan, it fails to bind the FIPV-II RBD, suggesting that divergent residues at the binding interface are also determinants." The discussion of pAPN recognition should be framed with prior context on glycan importance in host-virus interactions. Relevant literature should be cited before presenting results.
* P8. "The 22 key residues in cAPN responsible for binding to the FIPV-II RBD were highlighted and compared with 16 APN orthologs based on the complex structures in this study." A sequence alignment or structural view should be provided to justify why these 22 residues were selected.
* Likewise, on P8. "Residues at positions 378 and 779 differed the most frequently among the 17 APN orthologs." Consider generating a WebLogo sequence alignment for better visualization.
9. P6. "Of the 32 potential N-glycosylation sites identified ... 22 glycans ... resolved ... (Fig. 1B)." The authors need to provide a figure showing the superposition of the atomic model (with the N-glycans) onto the EM map to illustrate the direct visualization of the glycosylation. Where possible, the authors should carry out mass spectrometry-based glycoproteomics to ascertain the N-glycosylation of the putative N-glycosylation sites and to quantitative profile the N-glycosylation for modeling.
10. P6. "This monomorphic state closely mirrors the swung-out conformation observed in CCoV-HuPn-2018 (RMSD = 0.648) (Supplementary Fig. 1)." The unit of the RMSD (presumably Å) is missing. More importantly, structural alignments of large molecular assemblies such as spike proteins are often masked by the structural similarity of the main bodies of the S1/S2 subunit when rigid-body domain opening transition of the N-terminal D0 and/or RBD is compromised in the minimization processes. A better illustration of the structural different between different spike proteins should be the alignment of the S1/S2 subunit without the RBD and/or D0 followed by geometrical analysis of the rotation and/or displacement of the center of masses of the functional domains. For better visualization, the individual functional domains should also be colored differently as exemplified in this study https://www.cell.com/molecular-cell/pdf/S1097-2765(22)00266-0.pdf
11. P8. "To evaluate the cross-species receptor-binding potential of FIPV-II, we compared the binding capabilities of three alpha-CoV 1 members (FIPV-II, CCoV-HuPn-2018, and TGEV) RBDs to the APNs from 17 species, using flow cytometry (Fig. 4B)." The rationale for including SARS-CoV S-RBD as a control is missing and should be explained.
12. P9. "Flow cytometry results showed that all three viral RBDs from FIPV-II, CCoV-HuPn-2018, and TGEV could bind to APNs from three species including cats, red foxes, and dogs." Figure 4B suggests stronger or comparable binding of FIPV-II to fox APN relative to cat APN, yet the SPR-derived K_D values in Fig. 4C show the opposite. This discrepancy should be reconciled. Moreover, raw SPR sensorgrams and detailed figure legends (conditions, replicates, definitions of symbols) should be provided.
13. P6. "Although type I and type II FIPV S proteins share only 44.1% amino acid sequence identity ... (20)." The authors should

define the sequences (Uniprot IDs) of the type I and type II FIPV S proteins used for the alignment.
14. P7. "This prompted us to individually fit distinct domains ... (Fig. 2B)." Fig. 2B currently shows the three protomers in different colors without distinctions for the individual domains. The authors should make the necessary changes to the presentation of Fig. 2B to match the description herein.
15. Last line of the first paragraph of P7, "constraints imposed by the compact trimeric architecture (Fig. 2F)." There is no Fig. 2F. Please correct it. In the following paragraph, "Therefore, we incubated S protein with cAPN ... (Fig. S6)."Figure S6 should be corrected as S3 for consistency.

Minor comments:

1. P8, line 11 "To investigate the potential for cross-species transmission ... 17 representative species................" There are actually 18 sequences presented in Fig. 4A although the FACS results on Fig. 4B indeed showed 17. Why one of the 18 aligned sequences was omitted in the FACS analysis should be explained.
2. P4. "Based on the significant antigenic differences among FIPV ... (Jaimes et al., 2020)." The original classification report (Pedersen et al., 1984; https://link.springer.com/article/10.1007/BF01310494) should be cited instead of a secondary source.
3. When referring to the RMSD, the unit should be specified as Å or nm.
4. Provide GenBank accession numbers for animal APN orthologs.
5. Correct "pCDNA4.0" to "pcDNA4.0."
6. Fig. 7 legend,"red triangles" should be "red circles"
7. The PDB entries used in Fig. S1 should be included

Referee #2:

The COVID-19 pandemic has highlighted the importance of understanding how coronaviruses enter host cells and their potential for cross-species transmission. While research has largely focused on betacoronaviruses, members of other genera remain comparatively understudied. This manuscript examines the spike protein of type II feline infectious peritonitis virus (FIPV-II), an important veterinary pathogen in the genus Alphacoronavirus.

The authors combine structural and functional approaches to characterize the trimeric spike, its interaction with feline aminopeptidase N (APN), the breadth of its binding to APN orthologs from 17 species, and the ability of a subset of these orthologs to mediate infection.

To my knowledge, this work provides the first structural description of the FIPV-II spike, its receptor engagement, and the zoonotic constraints associated with this virus. These findings will be of particular interest to researchers studying coronaviruses and viral host range.

Overall, this study has the potential to make a valuable contribution to the literature; however, several issues should be addressed before publication.

Main Points to Address

1. Inclusion of FCoV-23 for comparative analysis

A notable omission from the manuscript is discussion of FCoV-23, a recently emerged recombinant alphacoronavirus reported by the Veesler and Tait-Burkhard groups[1,2]. Without an in-depth comparison of spike structures and RBD-APN interactions, it is difficult to assess the novelty of the structural work presented here.

2. Assessment of spike domain 0 and RBD conformational dynamics

The authors claim differences in domain 0 compared with previously published spike structures. However, it is unclear whether they have rigorously assessed the conformational dynamics of domain 0 and the RBD. Symmetry expansion and focused classification of these regions should be performed to identify potential alternative conformations, as described in other studies[3,4].

3. Lack of representative EM density

The figures do not include representative EM density maps, preventing readers from evaluating the quality of the structural data. Representative density for different regions of the spike should be included in the supplementary information. If invited to submit a revised manuscript, the authors should also provide the maps and models for reviewer assessment.

4. Issues with discussion

The authors present an overly black-and-white view of spike RBD dynamics, contrasting betacoronaviruses and gammacoronaviruses with members of other genera. Several studies contradict this simplified trend but are not cited. For example, for HKU1-A, spike opening has been shown to occur only after binding to a primary sialoglycan receptor[4], and an open structure of OC43 has not been observed[5-7]. All published gammacoronavirus spike protein structures to date have been captured in the closed conformation[8,9]. Conversely, spontaneous RBD opening has been observed for PEDV[10]. The discussion should be revised to incorporate these findings and present a more balanced interpretation of the literature.

Minor Comments

1) Check carefully for missing citations throughout the text. For examples, I see that DeepEMhancer was used but not appropriately referenced[11].
2) Figure call outs are stylistically inconsistent or incorrect and should be cross-checked.
3) Typos and grammatical errors occur throughout; careful proofreading is required.
4) RMSD values should not be reported to three decimal places, rounding to the first decimal place is sufficient.
5) I advise the authors to re-order the results and supplementary figures to follow a logical sequence for improved readability.

References

1. Attipa, C. et al. Feline infectious peritonitis epizootic caused by a recombinant coronavirus. Nature (2025) doi:10.1038/s41586-025-09340-0.
2. Tortorici, M. A. et al. Loss of FCoV-23 spike domain 0 enhances fusogenicity and entry kinetics. Nature (2025) doi:10.1038/s41586-025-09155-z.
3. Tortorici, M. A. et al. Structure, receptor recognition, and antigenicity of the human coronavirus CCoV-HuPn-2018 spike glycoprotein. Cell 185, (2022).
4. Pronker, M. F. et al. Sialoglycan binding triggers spike opening in a human coronavirus. Nature (2023) doi:10.1038/s41586-023-06599-z.
5. Wang, C. et al. Antigenic structure of the human coronavirus OC43 spike reveals exposed and occluded neutralizing epitopes. Nat Commun 13, (2022).
6. Alejandra Tortorici, M. et al. Structural basis for human coronavirus attachment to sialic acid receptors. Nat Struct Mol Biol 26, (2019).
7. Bangaru, S. et al. Structural mapping of antibody landscapes to human betacoronavirus spike proteins. Sci Adv 8, (2022).
8. Shang, J. et al. Cryo-EM structure of infectious bronchitis coronavirus spike protein reveals structural and functional evolution of coronavirus spike proteins. PLoS Pathog 14, (2018).
9. Hulswit, R. J. G. et al. Cetacean coronavirus spikes highlight S glycoprotein structural plasticity. bioRxiv 2025.05.20.655071 (2025) doi:10.1101/2025.05.20.655071.
10. Huang, C. Y. et al. In situ structure and dynamics of an alphacoronavirus spike protein by cryo-ET and cryo-EM. Nat Commun 13, (2022).
11. Sanchez-Garcia, R. et al. DeepEMhancer: a deep learning solution for cryo-EM volume post-processing. Commun Biol 4, (2021).

** As a service to authors, EMBO Press provides authors with the possibility to transfer a manuscript that one journal cannot offer to publish to another EMBO publication or the open access journal Life Science Alliance launched in partnership between EMBO Press, Rockefeller University Press and Cold Spring Harbor Laboratory Press. The full manuscript and if applicable, reviewers' reports, are automatically sent to the receiving journal to allow for fast handling and a prompt decision on your manuscript. For more details of this service, and to transfer your manuscript please click on Link Not Available. **

Please do not share this URL as it will give anyone who clicks it access to your account.

Referee #1:

EMBO type II FIPV report

Niu, et al. described the use of cryo-electron microscopy (cryo-EM) to determine the structures of the serotype II FIPV spike (S) protein in both apo form and in complex with its receptor. In addition, they provided a detailed analysis of the interaction between feline APN and the receptor-binding domain (RBD) of the FIPV-II S protein. The study also explored the molecular basis for cross-species infection by examining 17 APN orthologs, and it unambiguously demonstrated the importance of the N748-linked glycan and surrounding charged residues in receptor recognition. However, several concerns need to be addressed to strengthen the functional interpretation, data presentation, and reproducibility of the study. Specifically, the cryo-EM data quality requires further improvement to generate sufficient confidence to the model building and functional annotation. The current presentations appear too preliminary in terms of the structural analyses and presentation for the publication in EMBO Journal. Specific comments are listed below.

Response: Thank you for providing a thoughtful summary of our work. Your comments have been invaluable in helping us improve our manuscript, and we are truly grateful for your feedback.

Major comments:

1. As it currently stands, the cryo-EM maps do not have sufficient resolution and quality to support the descriptions of detailed molecular interactions (Figures 1-3 and more critically Figure 6). The authors need to provide clear structure-to-map superposition of the key contacts to demonstrate the map quality that supports the model building. Additionally, the authors are recommended to provide the Q-scores of the selected molecular contacts for a more unbiased quality evaluation.

Response: We apologize for the insufficient supporting structural information in the

original manuscript. According to the reviewer’s suggestion, we have now included **Figure EV9** to display a clear structure-to-map superposition of the key contact regions in the FIPV-1146 RBD–cAPN complex, demonstrating the quality of the cryo-EM maps that support our model interpretation. Furthermore, we have provided structure-to-map superpositions for the key glycosylation sites in the FIPV-1146 S protein, along with the corresponding Q-scores of the selected glycan densities, as presented in the newly added **Figure EV2**. We believe these additions allow for a more objective evaluation of the map quality underlying the described molecular interactions.

2. P8 The authors mentioned that "The 22 key residues in cAPN responsible for binding to the FIPV-II RBD were highlighted and compared with 16 APN orthologs based on the complex structures in this study." But there is no clear cross-referencing in the figure about these key residues. Presumably, the molecular details of the RBD:APN interactions are presented in Figure 6, but the number of residues illustrated in Figure 6 does not match the description on P8.

Response: We apologize for the oversight regarding the incomplete figure and inconsistent description. We have now added **Figure EV12**, which specifically illustrates the 22 key residues in cAPN involved in the interaction with the FIPV-1146 RBD. Additionally, both **Figure 4 and the corresponding text on P9** have been updated accordingly to ensure consistency between the figures and the description.

3. From the angular distributions of the observed particle images (Supplementary Fig. S4D and S5D), it is apparent that some complexes exhibit preferred orientation issues. The authors need to optimize their cryo-EM sample conditions to improve the preferred orientation issues.

Response: As noted by the reviewer, the two structures do exhibit slightly preferred orientation issues. Following the reviewer's suggestion, we attempted different

cryo-EM sample conditions for these two samples, including changing grids and adding glycerol; however, these attempts did not significantly improve the orientation distribution. Additionally, we tried incubating FIPV-1146 S with cAPN and purifying the complex via size-exclusion chromatography before preparing cryo-EM samples, but this still did not effectively alleviate the preferred orientation issues. Nevertheless, we were able to obtain density maps of reasonable quality and have updated the relevant cryo-EM supplementary figures (e.g., Figure EV1) to present our results more comprehensively.

4. The authors reported the cryo-EM map and rigid-body docking modeling of the dissociated S1 trimer (Figure 2), but it is unclear whether this structure is simply an experimental artifact or of functional relevance. The biological implication of the dissociated S1 trimer is not mentioned in the Discussion section, which should be appropriately addressed.

Response: Thank you for your constructive comments. We agree that it remains uncertain whether the S1 trimer observed in this study represents an experimental artifact or a biologically relevant structure. Therefore, we have moved the relevant results for the S1 trimer to the supplementary information for reference. Meanwhile, the biological implication of the dissociated S1 trimer has also been mentioned in the Discussion section on **P16**, as noted there: "**Concurrently, we readily identified disassociated FIPV-1146 S1 trimer particles, which exhibit a ring-like structure with all three RBDs in a standing conformation, aligning with a similar conformation reported for MERS-CoV (Yuan et al., 2017). It implicates that the S1 trimer with three standing RBD domains is easily disassociated from the S2 moiety, and thus the stable S trimer particles with three standing RBD domains was rarely observed. Notably, we cannot rule out the possibility that the disassociated S1 trimer may simply be an experimental artifact.**"

5. It is stated that the cryo-EM map of the dissociated S1 trimer was resolved to 4.8 Å

(Fig. 2) but not FSC curve was shown in Supplementary Fig. 4. Importantly, the map presented in Fig. 2A does not meet the expectation of such a resolution - beta-sheet separation and well-defined helix should be expected. The authors need to provide sufficient evidence to ensure that the descriptions of the structural quality match the actual experimental results. A minor point about the statement on P6 "However, disassociated FIPV-II S1 trimer particles ... at 4.8 Å (Fig. S2)." The cross-reference to Fig. S2 should be corrected to Fig. 2A.

Response: We sincerely apologize for not providing complete supporting data in the original submission. We have updated **Figure EV1** to include both the FSC curve and the angular distribution plot for the dissociated S1 trimer. Regarding the perceived map quality in Fig. EV1G, we acknowledge that the visual features may appear less defined than typically expected at the reported global resolution of 4.89 Å. This is likely attributable to the potential flexibility within the S1 trimer, factors which can limit local resolvability even when the global resolution estimate, as rigorously validated by the FSC curve (now provided). We have ensured that all descriptions of structural quality align with the experimental evidence. Furthermore, the cross-reference to Fig. S2 on page 6 has been corrected as indicated.

6. Through systematic biochemical and biophysical characterizations of Type II FIPV S protein interactions with the 17 APN orthologs, the authors established several APN's direct binding to the Type II FIPV S protein (Figures 4, 5 and 7). However, the cryo-EM map of the S trimer in complex the APN dimer (Figure 3 and Supplementary Fig. S4) require major improvements to assure sufficient confidence of the mode of binding between RBD and APN.

* The statement that "Additionally, during 2D classification, we identified the category of two S trimers bound to one cAPN dimer (Figs. 3C and D)." (end of P7) does not have strong support in Fig. 3. The selected 2D class images are too ambiguous to support such as statement.

Response: Thank you for your constructive comments. We agree that the originally

selected 2D class images were ambiguous and did not provide strong support for the claim regarding "two S trimers bound to one cAPN dimer." Therefore, we have removed the selected 2D class images from the revised manuscript. To directly address the binding mode between the S protein and cAPN, we adopted a strategy similar to that used in the recently published HCoV-229E S-APN complex (Tsai, Chien et al., 2025). This involved pre-purifying the S–cAPN complex via size-exclusion chromatography prior to grid preparation (**Fig EV7A**). Subsequent 3D classification of these data yielded a reconstruction at 3.53 Å resolution, which clearly reveals one cAPN dimer bound to two receptor-binding domains (RBDs) of the FIPV-1146 S trimer (**Figs. 3C and EV7F**). This new structural evidence provides a definitive basis for discussing the S-APN interaction.

* In addition to the preferred orientation issue, the low map resolution (5Å) is another serious issue for the modeling of the ternary complex.

Response: As the reviewer rightly notes, the low resolution of the cryo-EM map for the ternary complex is indeed a limitation. Although our initial S-APN structure captured their interaction, it did not clearly resolve the precise binding domain. To address this, we re-expressed the proteins and optimized sample preparation. From these new efforts, we obtained an intriguing result: the cAPN dimer appears to bind simultaneously to two S protein trimers, and the density suggests this interaction occurs specifically with the RBD domain (**Fig. 3C**). However, we were unfortunately unable to achieve a high-resolution reconstruction of the full complex. Nonetheless, by integrating this new data with our prior biochemical and biophysical evidence, we propose a model wherein the APN dimer bridges two S trimers via their RBDs. This mode of interaction is analogous to that observed in a recently published study (Tsai et al., 2025).

* There is no sufficient detail in the workflow on Supplementary Fig. 6 to ensure the reproducibility of the cryo-EM data processing.

Response: Thank you for your suggestion. We have revised and provided a more detailed description of the cryo-EM data processing workflow in **Figure EV6**, which now includes sufficient information to ensure the reproducibility of the analysis.

* Importantly, the authors need to provide sufficient details to describe how the atomic model was generated to avoid steric clashes between different functional domains required for the opening of the RBD as presented in Fig. 3.

Response: Thank you for raising this important methodological point. The initial model presented in Figure 3 was indeed speculative and intended to illustrate a hypothesized binding mode. We have now updated it through a defined workflow: **The atomic model was produced by generating a structure with AlphaFold2 (template: 8WDE), fitting and refining it in Coot against the cryo-EM maps (EMD-64643 and EMD-67259), and finally visualizing it in PyMOL**. The detailed methodology has been included in the corresponding figure legend (**Fig. 3D**).

7. P14. "we observed that a single cAPN dimer could simultaneously bind to two FIPV-II S trimers" The 2D class averages in Fig. 3C are ambiguous. Complementary methods such as mass photometry (MP) or SEC-MALS could help confirm the stoichiometry of this interaction.

Response: Thank you for this constructive suggestion. We acknowledge that the 2D class averages presented in the initial manuscript were ambiguous. To address this directly, we pursued higher-resolution structural analysis. As detailed in our revised **Figure 3C and Figure EV7**, 3D classification and reconstruction of the pre-formed S–cAPN complex now provide a definitive map at 3.53 Å resolution. This structure clearly demonstrates that a single cAPN dimer engages two RBDs from distinct S trimers, thereby offering direct structural evidence for the 2:1 (S trimer : cAPN dimer) interaction model.

8. Accordingly, the authors need to address the issues regarding RBD:APN interactions with clear supporting evidence:

* P10. "............cAPN also adopts an open conformation in the FIPV-II RBD-cAPN complex............." The authors should provide additional details on the open and closed conformations of APN. Did the cryo-EM data show heterogeneity between these states? Figure S5 suggests otherwise. It should also be clarified whether reference volumes were introduced during the heterogeneous refinement step (Fig. 5E).

Response: Thank you for your constructive comments. We have provided additional details regarding the conformational states of APN. Our previous studies, as well as another report from other group (Santiago, Mudgal et al., 2017, Tian, Sun et al., 2025), indicate that APN can adopt two distinct conformational states (open and closed) when interacting with different viral proteins. In the FIPV-1146 RBD-cAPN complex, we only observed the open conformation of cAPN. To illustrate this, we have included a comparative structural alignment of the open and closed APN conformations (**Fig. EV10**).

* P11. "cAPN formed a hydrogen bond network with amino acids (K524, Y528, S534, W571, and D572) of the FIPV-II RBD (Fig. 6B)." The manuscript should clarify the methodology/software used to define hydrogen bonds. The current figure does not explicitly show these interactions.

Response: We apologize for the inconsistency between the Figure 6 and the description. We have now corrected all amino acid numbering in both the **Figure 6** and the text on **P9** to ensure consistency. Furthermore, we have added the methodological details for hydrogen bond analysis in the **Figure 6 legend**. Specifically, **H-bonds were identified using PDBePISA (https://www.ebi.ac.uk/msd-srv/prot_int/pistart.html).** These updates ensure both reproducibility and scientific rigor in our structural analysis.

* P11. "While pig APN (pAPN) retains the N748-linked glycan, it fails to bind the FIPV-II RBD, suggesting that divergent residues at the binding interface are also determinants." The discussion of pAPN recognition should be framed with prior context on glycan importance in host-virus interactions. Relevant literature should be cited before presenting results.

Response: Thank you for the valuable suggestion. A section introducing relevant prior literature has been added to the Results before presenting our experimental findings. Specifically, we have included the following description on **P12-13**:

"**It is well-established that APN serves as the functional receptor for the alpha-CoV-1, and N-linked glycosylation of APN (notably at N748) has been demonstrated to be a critical determinant for viral entry. For instance, the interaction between TGEV and pig APN (pAPN) is dependent on the N748-linked glycosylation (Reguera et al., 2012, Tian et al., 2025). Similarly, the recently identified CCoV-2018-HuPn and FCoV-23 also rely on glycosylated APN for host cell recognition (Tortorici et al., 2025, Tortorici et al., 2022).**"

This addition provides the necessary background, allowing for a more logically structured presentation of our subsequent results regarding pAPN recognition by the FIPV-1146 RBD.

* P8. "The 22 key residues in cAPN responsible for binding to the FIPV-II RBD were highlighted and compared with 16 APN orthologs based on the complex structures in this study." A sequence alignment or structural view should be provided to justify why these 22 residues were selected.

Response: Thank you for your constructive comments. We have added the new **Figure EV12A**, which provides a sequence view of cAPN that specifically highlights the 22 key residues identified to interact with the FIPV-1146 RBD, thereby justifying their selection based on our structural data.

* Likewise, on P8. "Residues at positions 378 and 779 differed the most frequently among the 17 APN orthologs." Consider generating a WebLogo sequence alignment for better visualization.

Response: As recommended, we have generated a WebLogo sequence alignment to better visualize the conservation patterns, which is now included as **Figure EV12C**. This clearly illustrates that **the residues at positions 751, 779, 783 and 798 differed the most frequently among the 17 APN orthologs** (**P11**).

9. P6. "Of the 32 potential N-glycosylation sites identified ... 22 glycans ... resolved ... (Fig. 1B)." The authors need to provide a figure showing the superposition of the atomic model (with the N-glycans) onto the EM map to illustrate the direct visualization of the glycosylation. Where possible, the authors should carry out mass spectrometry-based glycoproteomics to ascertain the N-glycosylation of the putative N-glycosylation sites and to quantitative profile the N-glycosylation for modeling.

Response: Thank you for your constructive suggestions. First, we have provided structure-to-map superpositions for the key glycosylation sites in the FIPV-1146 S protein, as presented in the newly added **Figure EV2**. This provides direct visualization of the glycosylation in the EM map. Furthermore, as recommended, we have performed mass spectrometry-based glycoproteomics analysis to confirm the N-glycosylation at the putative sites. The detailed methodology and results are added in the revised manuscript (**Figure 2; P7, P19-21**).

10. P6. "This monomorphic state closely mirrors the swung-out conformation observed in CCoV-HuPn-2018 (RMSD = 0.648) (Supplementary Fig. 1)." The unit of the RMSD (presumably Å) is missing. More importantly, structural alignments of large molecular assemblies such as spike proteins are often masked by the structural similarity of the main bodies of the S1/S2 subunit when rigid-body domain opening

transition of the N-terminal D0 and/or RBD is compromised in the minimization processes. A better illustration of the structural different between different spike proteins should be the alignment of the S1/S2 subunit without the RBD and/or D0 followed by geometrical analysis of the rotation and/or displacement of the center of masses of the functional domains. For better visualization, the individual functional domains should also be colored differently as exemplified in this study https://www.cell.com/molecular-cell/pdf/S1097-2765(22)00266-0.pdf

Response: Thank you for your constructive suggestions. We have addressed both points raised. Firstly, the unit of the RMSD (Å) has now been added to the relevant description. More importantly, to better illustrate the structural differences, we have performed separate alignments focusing on the S1 subunits. This approach helps mitigate potential masking effects from the conserved S2 region during whole-spike alignment. As suggested, the individual functional domains within these structures are now colored differently for clearer visualization. These updated analyses are presented in the revised **Figure EV4**.

11. P8. "To evaluate the cross-species receptor-binding potential of FIPV-II, we compared the binding capabilities of three alpha-CoV 1 members (FIPV-II, CCoV-HuPn-2018, and TGEV) RBDs to the APNs from 17 species, using flow cytometry (Fig. 4B)." The rationale for including SARS-CoV S-RBD as a control is missing and should be explained.

Response: The rationale for including the SARS-CoV-2 RBD in the experiment has been added to the Results section on **P10**. Specifically, since SARS-CoV-2 utilizes angiotensin-converting enzyme 2 (ACE2) as its primary receptor and does not bind to APN, it was employed as a negative control.

12. P9. "Flow cytometry results showed that all three viral RBDs from FIPV-II, CCoV-HuPn-2018, and TGEV could bind to APNs from three species including cats,

red foxes, and dogs." Figure 4B suggests stronger or comparable binding of FIPV-II to fox APN relative to cat APN, yet the SPR-derived K_D values in Fig. 4C show the opposite. This discrepancy should be reconciled. Moreover, raw SPR sensorgrams and detailed figure legends (conditions, replicates, definitions of symbols) should be provided.

Response: Thank you for your insightful comment regarding the discrepancy between the flow cytometry and SPR results. Indeed, flow cytometry can serve as a qualitative assay to evaluate the binding capacity between two molecules, but it is generally not sufficient for quantitative affinity measurements, which typically require techniques such as surface plasmon resonance (SPR). In our flow cytometry results, the binding signal of FIPV-1146 RBD to red fox APN appeared stronger or comparable to that of cat APN. However, SPR assays revealed that the affinity of FIPV-1146 RBD for fox APN is lower than for cat APN. This discrepancy may arise from differences in molecular conformation between membrane-bound APN expressed on the cell surface (as used in flow cytometry) and the soluble, secreted form of APN used in SPR experiments, which could influence RBD binding. We have now provided the raw SPR sensorgrams and detailed figure legends (including experimental conditions, number of replicates, and definitions of symbols) has been added in **Figure EV13**.

13. P6. "Although type I and type II FIPV S proteins share only 44.1% amino acid sequence identity ... (20)." The authors should define the sequences (Uniprot IDs) of the type I and type II FIPV S proteins used for the alignment.

Response: Done.

14. P7. "This prompted us to individually fit distinct domains ... (Fig. 2B)." Fig. 2B currently shows the three protomers in different colors without distinctions for the individual domains. The authors should make the necessary changes to the presentation of Fig. 2B to match the description herein.

Response: We apologize for the mismatch between the figure and the description in the text. We have updated **Figure EV5** to clearly distinguish the individually fitted domains, which now aligns with the description on **P7**.

15. Last line of the first paragraph of P7, "constraints imposed by the compact trimeric architecture (Fig. 2F)." There is no Fig. 2F. Please correct it. In the following paragraph, "Therefore, we incubated S protein with cAPN ... (Fig. S6)."Figure S6 should be corrected as S3 for consistency.

Response: We apologize for the figure citation errors. We have updated the relevant figures citation in the revised manuscript to match the corresponding figures.

Minor comments:

1. P8, line 11 "To investigate the potential for cross-species transmission ... 17 representative species................" There are actually 18 sequences presented in Fig. 4A although the FACS results on Fig. 4B indeed showed 17. Why one of the 18 aligned sequences was omitted in the FACS analysis should be explained.

Response: We apologize for this oversight. The alignment in the original Fig. 4A inadvertently included an additional sequence (monkey APN), which was not part of the functional assays presented in the original Fig. 4B. This extra sequence has now been removed from the figure to ensure consistency between the sequence alignment and the corresponding FACS data (**Fig. EV12**).

2. P4. "Based on the significant antigenic differences among FIPV ... (Jaimes et al., 2020)." The original classification report (Pedersen et al., 1984; https://link.springer.com/article/10.1007/BF01310494) should be cited instead of a secondary source.

Response: As recommended, we have now cited the original classification report by Hohdatsu et al. (1991) and Pedersen et al. (1984) in place of the secondary source

(Jaimes et al., 2020) of our revised manuscript (**P4**). This change ensures the most direct and appropriate referencing of the foundational FIPV serotype classification.

3. When referring to the RMSD, the unit should be specified as Å or nm.

Response: Done.

4. Provide GenBank accession numbers for animal APN orthologs.

Response: The relevant GenBank accession numbers for all APN sequences have been detailed in our recently paper (Tian et al., 2025), which has been appropriately cited in the manuscript (**P18**).

5. Correct "pCDNA4.0" to "pcDNA4.0."

Response: Done.

6. Fig. 7 legend,"red triangles" should be "red circles"

Response: Done.

7. The PDB entries used in Fig. S1 should be included

Response: Done.

Referee #2:

The COVID-19 pandemic has highlighted the importance of understanding how coronaviruses enter host cells and their potential for cross-species transmission. While research has largely focused on betacoronaviruses, members of other genera remain comparatively understudied. This manuscript examines the spike protein of type II

feline infectious peritonitis virus (FIPV-II), an important veterinary pathogen in the genus Alphacoronavirus.

The authors combine structural and functional approaches to characterize the trimeric spike, its interaction with feline aminopeptidase N (APN), the breadth of its binding to APN orthologs from 17 species, and the ability of a subset of these orthologs to mediate infection.

To my knowledge, this work provides the first structural description of the FIPV-II spike, its receptor engagement, and the zoonotic constraints associated with this virus. These findings will be of particular interest to researchers studying coronaviruses and viral host range.

Overall, this study has the potential to make a valuable contribution to the literature; however, several issues should be addressed before publication.

Response: Thank you for providing a thoughtful summary of our work. Your comments have been invaluable in helping us improve our manuscript, and we are truly grateful for your feedback.

Main Points to Address

1. Inclusion of FCoV-23 for comparative analysis

A notable omission from the manuscript is discussion of FCoV-23, a recently emerged recombinant alphacoronavirus reported by the Veesler and Tait-Burkhard groups [1,2]. Without an in-depth comparison of spike structures and RBD-APN interactions, it is difficult to assess the novelty of the structural work presented here.

Response: We thank the reviewer for this constructive suggestion and for highlighting the two recent high-impact studies on FCoV-23, which were published during the submission process of our work. We agree that a comparative analysis is crucial for contextualizing our structural findings. We have now incorporated an in-depth comparison of the spike protein structures and their RBD-APN interaction modes between FCoV-23 and the virus central to our study. This new analysis has been added as a dedicated subsection in the Results section (**P6 and P8-9**) and is

summarized in the new figures (**Figs. 4, EV3 and EV4**).

2. Assessment of spike domain 0 and RBD conformational dynamics

The authors claim differences in domain 0 compared with previously published spike structures. However, it is unclear whether they have rigorously assessed the conformational dynamics of domain 0 and the RBD. Symmetry expansion and focused classification of these regions should be performed to identify potential alternative conformations, as described in other studies [3,4].

Response: We thank you for your constructive suggestion. We applied these methods to our cryo-EM data. As noted, our detailed classification of the FIPV-1146 S trimer did not yield a stable, well-resolved class with a proximal D0 conformation analogous to those in CCoV-HuPn-2018 and FCoV-23. This difference may stem from variations in sample preparation or purification. Importantly, however, the 3D classification of the FIPV-1146 S–cAPN complex data revealed a distinct subset of particles. This class, presented in the new **Figure EV7E**, displays clear density indicative of mixed D0 conformations within a single trimer (EMD-67940). While the local resolution in this flexible region is insufficient to build an atomic model, the reconstructed map robustly demonstrates the coexistence of different D0 states. We have revised the manuscript to include this new analysis on **P8**.

3. Lack of representative EM density

The figures do not include representative EM density maps, preventing readers from evaluating the quality of the structural data. Representative density for different regions of the spike should be included in the supplementary information. If invited to submit a revised manuscript, the authors should also provide the maps and models for reviewer assessment.

Response: According to the reviewer's suggestion, we have now included **Figure EV9** to display a clear structure-to-map superposition of the key contact regions in the FIPV-1146–cAPN complex, demonstrating the quality of the cryo-EM maps that

support our model interpretation. Furthermore, we have provided structure-to-map superpositions for the key glycosylation sites in the FIPV-1146 S protein, as presented in the newly added **Figure EV2**. We believe these additions allow for a more objective evaluation of the map quality underlying the described molecular interactions.

As requested, we have deposited the final refined cryo-EM density maps and corresponding atomic coordinate files for all structures reported in this study in the public repository. All deposited maps and models have now been fully released and are publicly accessible for direct download. These accession numbers and a statement regarding data availability have been added to the **Data Availability** section of the manuscript.

4. Issues with discussion

The authors present an overly black-and-white view of spike RBD dynamics, contrasting betacoronaviruses and gammacoronaviruses with members of other genera. Several studies contradict this simplified trend but are not cited. For example, for HKU1-A, spike opening has been shown to occur only after binding to a primary sialoglycan receptor [4], and an open structure of OC43 has not been observed [5-7]. All published gammacoronavirus spike protein structures to date have been captured in the closed conformation [8,9]. Conversely, spontaneous RBD opening has been observed for PEDV [10]. The discussion should be revised to incorporate these findings and present a more balanced interpretation of the literature.

Response: We sincerely thank the reviewer for this critical and constructive feedback regarding our discussion of spike RBD dynamics across coronavirus genera. We appreciate the reviewer pointing out the key contradictory examples from the literature, which we had unfortunately overlooked. In response, we have thoroughly revised the **Discussion section** on **P15** to provide a more nuanced and balanced interpretation.

Minor Comments

1) Check carefully for missing citations throughout the text. For examples, I see that DeepEMhancer was used but not appropriately referenced [11].

Response: We thank the reviewer for your meticulous reading and for identifying this oversight. We have now performed a thorough, line-by-line check of the entire manuscript to identify and correct any instances of missing or incomplete citations.

2) Figure call outs are stylistically inconsistent or incorrect and should be cross-checked.

Response: We thank the reviewer for highlighting this important issue. We have now performed a thorough, line-by-line cross-check of all figure callouts and have standardized their formatting to ensure strict consistency with EMBO J's style guide. We believe this careful revision has resolved the concern raised.

3) Typos and grammatical errors occur throughout; careful proofreading is required.

Response: We have now performed a careful, thorough proofread of the entire manuscript to correct these errors.

4) RMSD values should not be reported to three decimal places, rounding to the first decimal place is sufficient.

Response: Done.

5) I advise the authors to re-order the results and supplementary figures to follow a logical sequence for improved readability.

Response: In the revised version, we have re-ordered both the main and supplementary figures to follow a clearer narrative progression. We believe this

reorganization significantly improves the reader's experience.

References

1. Attipa, C. et al. Feline infectious peritonitis epizootic caused by a recombinant coronavirus. Nature (2025) doi:10.1038/s41586-025-09340-0.

2. Tortorici, M. A. et al. Loss of FCoV-23 spike domain 0 enhances fusogenicity and entry kinetics. Nature (2025) doi:10.1038/s41586-025-09155-z.

3. Tortorici, M. A. et al. Structure, receptor recognition, and antigenicity of the human coronavirus CCoV-HuPn-2018 spike glycoprotein. Cell 185, (2022).

4. Pronker, M. F. et al. Sialoglycan binding triggers spike opening in a human coronavirus. Nature (2023) doi:10.1038/s41586-023-06599-z.

5. Wang, C. et al. Antigenic structure of the human coronavirus OC43 spike reveals exposed and occluded neutralizing epitopes. Nat Commun 13, (2022).

6. Alejandra Tortorici, M. et al. Structural basis for human coronavirus attachment to sialic acid receptors. Nat Struct Mol Biol 26, (2019).

7. Bangaru, S. et al. Structural mapping of antibody landscapes to human betacoronavirus spike proteins. Sci Adv 8, (2022).

8. Shang, J. et al. Cryo-EM structure of infectious bronchitis coronavirus spike protein reveals structural and functional evolution of coronavirus spike proteins. PLoS Pathog 14, (2018).

9. Hulswit, R. J. G. et al. Cetacean coronavirus spikes highlight S glycoprotein structural plasticity. bioRxiv 2025.05.20.655071 (2025) doi:10.1101/2025.05.20.655071.

10. Huang, C. Y. et al. In situ structure and dynamics of an alphacoronavirus spike protein by cryo-ET and cryo-EM. Nat Commun 13, (2022).

11. Sanchez-Garcia, R. et al. DeepEMhancer: a deep learning solution for cryo-EM volume post-processing. Commun Biol 4, (2021).

Response: According to your suggestions, we have appropriately cited the above-mentioned references in relevant sections of the manuscript.

Reference

Santiago C, Mudgal G, Reguera J, Recacha R, Albrecht S, Enjuanes L, Casasnovas JM (2017) Allosteric inhibition of aminopeptidase N functions related to tumor growth and virus infection. *Sci Rep* 7: 46045

Tian Y, Sun J, Hou X, Liu Z, Chen Z, Pan X, Wang Y, Ren J, Zhang D, Yang B, Si L, Bi Y, Liu K, Shang G, Tian WX, Wang Q, Gao GF, Niu S (2025) Cross-species recognition of two porcine coronaviruses to their cellular receptor aminopeptidase N of dogs and seven other species. *PLoS Pathog* 21: e1012836

Tsai YX, Chien YC, Hsu MF, Khoo KH, Hsu SD (2025) Molecular basis of host recognition of human coronavirus 229E. *Nat Commun* 16: 2045

1st Revision - Editorial Decision 20th Feb 2026

Dear Dr. Niu,

Thank you for submitting the revised version of your manuscript to The EMBO Journal The study has now been seen by both original referees, and I have included their comments below.

While reviewer #2 is satisfied with the revision, reviewer #1 finds that several of their initial points were not sufficiently addressed or clarified. Since the raised concerns appear addressable, I would like to invite you to address the remaining referee comments in an exceptional round of additional revision.

The comments by the reviewer on the quality of the structural data are essential to address at this revision. While point 4 by reviewer #1 was not raised in the previous assessment round, please nevertheless consider including this analysis in the revision, since it would increase the rigour of the study.

Please note that, if the requested improvements cannot be implemented, your study could be a good candidate for publication in our sister journal EMBO Reports.

As a matter of policy, competing manuscripts published during this period will not negatively impact on our assessment of the conceptual advance presented by your study. However, please contact me as soon as possible upon publication of any related work to discuss the appropriate course of action.

When preparing your letter of response to the referees' comments, please bear in mind that this will form part of the Review Process File and will therefore be available online to the community. For more details on our Transparent Editorial Process, please visit our website: https://link.springer.com/partners/embo-press/editorial-policies#Peer%20review. Please also see the attached document with instructions for guidelines on preparation of the revised manuscript.

Please feel free to contact me if have any questions regarding this final revision. Thank you again for giving us the chance to consider your manuscript for The EMBO Journal. I look forward to receiving the revised version.

With best wishes,

Ieva Gailite

Ieva Gailite, PhD
Senior Scientific Editor
The EMBO Journal
Meyerhofstrasse 1
D-69117 Heidelberg
Tel: +4962218891309
i.gailite@embojournal.org

Instructions for preparing your revised manuscript:

Read our guidance for manuscript revisions and related editorial policies: https://link.springer.com/journal/44318/submission-guidelines#cms-Revised-submissions

Please make sure you upload a letter of response to the referees' comments together with the revised manuscript.

Please also check that the title and abstract of the manuscript are brief, yet explicit, even to non-specialists.

When assembling figures, please refer to our figure preparation guideline in order to ensure proper formatting and readability in print as well as on screen:
https://media.springernature.com/original/springer-cms/rest/v1/content/27825798/data/v1

At EMBO Press we ask authors to provide source data for the main manuscript figures. You will receive a separate email with instructions for providing source data with your revised manuscript, including how to upload and organize the files.

IMPORTANT: When you send the revision we will require
- a point-by-point response to the referees' comments, with a detailed description of the changes made (as a word file).
- a word file of the manuscript text.

- individual production quality figure files (one file per figure)
- a complete author checklist
- Expanded View files (replacing Supplementary Information)
- a Reagents and Tools Table as part of the Methods section

Please remember: Digital image enhancement is acceptable practice, as long as it accurately represents the original data and conforms to community standards. If a figure has been subjected to significant electronic manipulation, this must be noted in the figure legend or in the 'Materials and Methods' section. The editors reserve the right to request original versions of figures and the original images that were used to assemble the figure.

We realize that it is difficult to revise to a specific deadline. In the interest of protecting the conceptual advance provided by the work, we recommend a revision within 3 months (21st May 2026). Please discuss the revision progress ahead of this time with the editor if you require more time to complete the revisions.

Referee #1:

The authors have made significant improvements in the revised manuscript to address the reviewer comments. However, there are some key technical issues remaining as listed below. It would be important to address these issues before the manuscript is accepted for publication.

1. The authors's effort to profile the N-glycosylation of the FIPV spike protein is commendable. However, the raw dataset needs to be deposited in the MassIVE Repository https://massive.ucsd.edu/ and the entry ID reported in the manuscript in order to fulfill the requirement of data transparency. Importantly, the revised manuscript lacks sufficient details about how the quantification of different N-glycoforms was achieved by Byonic. Was it done simply by peptide counts (PSM)? More quantitative open-source approaches are available, e.g., https://doi.org/10.1038/s41592-021-01306-0, but it is important to at least define clearly how the revised Figure 2A/B was generated. Mass spectrometry glycoproteomics tend to underestimate the amount of sialylated glycopeptides (and complex type N-glycans). What precautions were carried to minimize such a bias in the glycoproteomics analysis?

2. Table EV2 lists all the putative N-glycosylation sites. 15 of the 27 putative N-glycosylation sites were not identified in the MS glycoproteomics analysis but the cryo-EM analysis did observe the N-glycosylation in these missing sites, except residues 1314 onwards in the stalk region due to intrinsic flexibility. The missing N-glycosylation site should be indicated in Figure 2A with the domain definition (Figure 2B) included.

3. In the Materials and Method section, the authors reported that "The immobilized protein was separately digested into peptides with 0.5 µg/µL trypsin at 37 {degree sign}C, shaken at 1000 rpm for 16 hours, or with 0.5 µg/µL chymotrypsin at 25 {degree sign}C for 16 hours, both at an enzyme-to-substrate ratio of 1:50 (w/w)." It implies that there were two independent MS glycoproteomics runs (samples) but Figure 2A/B reports only one set of data. The authors should explain how were the two separately digested glycopeptides used to generate the results.

4. Importantly, to improve the coverage of the N-glycosylation analysis, the authors are advised to include other proteases such as Glu-C and alpha-lytic protease to obtain other glycopeptide combinations in cases where the protein sequence of the FIPV spike protein does not favor trypsin and chymotrypsin digestion.

5. The ability to obtain the FIPV spike:cAPN cryo-EM structure in the 6:2 binding stoichiometry in Figure 3 is a major breakthrough. However, the spike protein part in Figure 3c clearly suffers from dynamics-associated map fragmentation. According to Figure EV7, the reported cryo-E< of the FIPV spike:cAPN cryo-EM structure in the 6:2 binding stoichiometry was directly taken after a global heterogeneous refinement without masking/local refinement. Given the intrinsic dynamic nature of the system, the authors need to carry out local refinements by masking important functional domains/segments to improve the local resolution, and merge them into a consensus map as routinely done in contemporary cryo-EM single particle analyses.

6. Likewise, the cryo-EM map of the single spike trimer bound for a cAPN dimer (Figure 3a) needs to be improved by local refinement of the spike, RBD and APN individually.

7. Can the authors include the Uniprot IDs of the APNs used in the multiple sequence alignment in Figure EV12?

Referee #2:

The authors have made substantial efforts to address both my concerns and those of Reviewer 1 regarding the manuscript. As a result, the key claims are better substantiated, and a more nuanced interpretation of the results and the wider literature is provided. I consider my concerns to have been adequately addressed, and I believe the resulting manuscript will make a valuable contribution to EMBO Journal.

One minor additional note for the authors: the term "electron density" should be avoided in the context of cryo-EM, where maps represent Coulomb (electrostatic) potential. The terms "Coulomb potential map," "EM density," or "potential map" are usually preferred.

Referee #1:

The authors have made significant improvements in the revised manuscript to address the reviewer comments. However, there are some key technical issues remaining as listed below. It would be important to address these issues before the manuscript is accepted for publication.

1. The authors's effort to profile the N-glycosylation of the FIPV spike protein is commendable. However, the raw dataset needs to be deposited in the MassIVE Repository https://massive.ucsd.edu/ and the entry ID reported in the manuscript in order to fulfill the requirement of data transparency. Importantly, the revised manuscript lacks sufficient details about how the quantification of different N-glycoforms was achieved by Byonic. Was it done simply by peptide counts (PSM)? More quantitative open-source approaches are available, e.g., https://doi.org/10.1038/s41592-021-01306-0, but it is important to at least define clearly how the revised Figure 2A/B was generated. Mass spectrometry glycoproteomics tend to underestimate the amount of sialylated glycopeptides (and complex type N-glycans). What precautions were carried to minimize such a bias in the glycoproteomics analysis?

Response: Thank you for your suggestive comment. The raw LC-MS/MS data and associated Byonic project files have been deposited in the ProteomeXchange Consortium (http://proteomecentral.proteomexchange.org) via the iProX partner repository under the dataset identifier **PXD077720**.

Regarding quantification of glycoforms, based on the retention times and m/z values, intensities of all glycopeptides were manually checked for areas and corrected for charge states and all charge states for a single glycopeptide were summed. We provided the details in Materials and Method section about classification of glycan types as well as captions in **Appendix Table S2** (for N-glycoforms) and **Appendix Table S3** (for O-glycoforms).

To minimize the underestimation of sialylated glycopeptides, preparation of glycopeptides was performed in reaction buffers with pH > 5 to avoid acidic hydrolysis

and prolonged lyophilization was avoided. All lyophilized glycopeptides were stored at -20 ºC until used. In addition, we employed stepped collision energy (SCE) higher-energy dissociation, HCD, and HCD product ions-triggered electron-transfer/higher-energy collision dissociation to improve the detection of sialylated glycopeptides.

Byonic® software has been widely used for glycosylation of other viruses, for example, SARS-CoV-2 spike glycoprotein as reported in *Science* 2020, *369* (6501), 330-333, as well as "O-Follow-N" pattern, for example, SARS-CoV-2 spike glycoprotein as reported in *Cell Research* 2021, *31*, 1123-1125. Therefore, to the focus of this study as well as future comparison, Byonic® is still suitable platform used in this research.

2. Table EV2 lists all the putative N-glycosylation sites. 15 of the 27 putative N-glycosylation sites were not identified in the MS glycoproteomics analysis but the cryo-EM analysis did observe the N-glycosylation in these missing sites, except residues 1314 onwards in the stalk region due to intrinsic flexibility. The missing N-glycosylation site should be indicated in Figure 2A with the domain definition (Figure 2B) included.

Response: Thank you for your comment. After more detailed glycosylation analysis, we were able to detect 35 N-glycosylation sites, in agreement with the canonical N-sequon. We provided details about the detected N-sites in the figure captions of **Figure 2A and B**.

3. In the Materials and Method section, the authors reported that "The immobilized protein was separately digested into peptides with 0.5 μg/μL trypsin at 37 {degree sign}C, shaken at 1000 rpm for 16 hours, or with 0.5 μg/μL chymotrypsin at 25 {degree sign}C for 16 hours, both at an enzyme-to-substrate ratio of 1:50 (w/w)." It implies that there were two independent MS glycoproteomics runs (samples) but Figure 2A/B reports only one set of data. The authors should explain how were the two separately

digested glycopeptides used to generate the results.

Response: Thank you for your comment. As suggested by Referee #1, to improve the coverage of glycosylation analysis, we performed sample treatment with five different proteases, additional treatment with PNGase F in $H_2{}^{18}O$ or O-glycosidase (IMPa), and used two fragmentation methods (stepped collision energy (SCE) higher-energy dissociation [HCD] and HCD product ion-triggered electron-transfer/higher-energy collision dissociation) and extended the acquisition time. Therefore, the details of the mass spectrometry analysis have been revised and highlighted in the manuscript.

4. Importantly, to improve the coverage of the N-glycosylation analysis, the authors are advised to include other proteases such as Glu-C and alpha-lytic protease to obtain other glycopeptide combinations in cases where the protein sequence of the FIPV spike protein does not favor trypsin and chymotrypsin digestion.

Response: Thank you for your suggestion. In our revised manuscript, in addition to trypsin/Lys-C and chymotrypsin, we performed enzymatic digestion on the FIPV spike protein using α-lytic protease, Gluc-C, and elastase. Moreover, we performed additional treatment with PNGase F in $H_2{}^{18}O$ or O-glycoprotease (IMPa) for complete mapping of glycosylation. As a result, we detected all 35 expected N-sites, among which only N565 could be detected as a deamidated peptide. A detailed analysis of the N-glycosylation sites is reported in **Figure 2 and Appendix Table S2.**

5. The ability to obtain the FIPV spike:cAPN cryo-EM structure in the 6:2 binding stoichiometry in Figure 3 is a major breakthrough. However, the spike protein part in Figure 3c clearly suffers from dynamics-associated map fragmentation. According to Figure EV7, the reported cryo-EM of the FIPV spike:cAPN cryo-EM structure in the 6:2 binding stoichiometry was directly taken after a global heterogeneous refinement without masking/local refinement. Given the intrinsic dynamic nature of the system, the authors need to carry out local refinements by masking important functional domains/segments to improve the local resolution, and merge them into a consensus

map as routinely done in contemporary cryo-EM single particle analyses.

Response: Thank you for your insightful suggestion. We agree that the proposed approach, which uses local refinements with masking of functional domains, can often improve map quality and structural interpretability. In response to the reviewer's concern, we have performed local refinements and masking strategies on the relevant functional domains. Meanwhile, we generated a template from the PDB:8WDE structure (the HCoV-229E spike:hAPN complex), trained a model using Topaz Train, and performed effective particle picking. Although these attempts led to a modest improvement in local resolution, they did not result in any substantial enhancement of the map density for the spike protein regions. Given that even after such treatments the map quality remains limited by the intrinsic dynamic nature of the system, we believe that the current global map, despite its suboptimal local features, still reliably supports the major conclusion of a 6:2 binding stoichiometry between FIPV spike and cAPN.

6. Likewise, the cryo-EM map of the single spike trimer bound for a cAPN dimer (Figure 3a) needs to be improved by local refinement of the spike, RBD and APN individually.

Response: Thank you for your constructive suggestion. As with the previous point regarding the 6:2 complex, we attempted local refinements on the single spike trimer bound to a cAPN dimer (Figure 3A) by applying local refinement for the spike, RBD, and APN regions. Although these local refinement attempts led to only a partial improvement in local resolution, the resolution remains insufficient to allow proper reconstruction of the different domains within the S protein. Nonetheless, the current map still sufficiently supports the key conclusions presented in the Results section.

7. Can the authors include the Uniprot IDs of the APNs used in the multiple sequence alignment in Figure EV12?

Response: We have now provided a new **Reagents and Tools Table** containing the Uniprot IDs for all APN sequences used in the multiple sequence alignment shown in

Appendix Fig. S12. We believe this addition enhances the clarity and reproducibility of our analysis.

Referee #2:

The authors have made substantial efforts to address both my concerns and those of Reviewer 1 regarding the manuscript. As a result, the key claims are better substantiated, and a more nuanced interpretation of the results and the wider literature is provided. I consider my concerns to have been adequately addressed, and I believe the resulting manuscript will make a valuable contribution to EMBO Journal.
One minor additional note for the authors: the term "electron density" should be avoided in the context of cryo-EM, where maps represent Coulomb (electrostatic) potential. The terms "Coulomb potential map," "EM density," or "potential map" are usually preferred.

Response: We are pleased to hear that our efforts have addressed your concerns. We appreciate your positive assessment and believe your feedback has significantly improved the quality and depth of our study.

We also thank you for the additional note regarding terminology. We have carefully reviewed the manuscript and corrected all instances of "electron density" to more appropriate terms "EM density" to accurately reflect the context of cryo-EM. These changes have been highlighted in the revised version.

Dear Dr. Niu,

Thank you for submitting a revised version of your manuscript. I sincerely apologise for the unusually prolonged assessment process for your study. I have now received positive assessment from reviewer #1 on the performed revisions. Therefore, there now remain only a few editorial and formatting points that need to be addressed before I can extend official acceptance of the manuscript:

1. Please note that it is The EMBO Journal policy for the transcript of the editorial process (containing referee reports and your response letters) to be published as an online supplement to each paper. If you should prefer removal of any referee-only figures included in the point-by-point response(s), e.g. because they may still be used for future publication or because they have been reproduced from published work by others, please do let us know immediately via response email.
More information is available here: https://www.embopress.org/transparent-process#Review_Process
2. We are missing the ORCID iDs for the co-corresponding authors Wen-xia Tian and Junqing Sun. In order to link the ORCID iD to the account in our manuscript tracking system, the author in question has to do the following:
- Click the 'Modify Profile' link at the bottom of your homepage in our system.
- On the next page you will see a box halfway down the page titled ORCID*. Below this box is red text reading 'To Register/Link to ORCID, click here'. Please follow that link: you will be taken to ORCID where you can log in to your account (or create an account if you don't have one)
- You will then be asked to authorise the access to your ORCID information. Once you have approved the linking, you will be brought back to our manuscript system.
Unfortunately, we cannot do this linking on the author's behalf for security reasons.
3. The manuscript sections should be in the following order: Title page - Abstract - Introduction - Results - Discussion - Methods - Data Availability - Acknowledgments - Disclosure and Competing Interests Statement - References - Figure Legends - (Main Tables with legends if applicable) - Expanded View Figure Legends.
4. CRediT has replaced the traditional author contributions section because it offers a systematic, machine-readable author contributions format that allows for more effective research assessment. Please remove the Authors Contributions from the manuscript and use the free text boxes beneath each contributing author's name in our online submission system to add specific details on the author's contribution. More information is available in our guide to authors.
5. Author affiliations with biotech companies should be stated in the "Disclosure and competing interests statement".
6. Please update references according to The EMBO Journal style - where there are more than 10 authors on a paper, the first 10 should be listed, followed by 'et al.'
7. Figure panels 4C-E are not mentioned in the manuscript text; please add the corresponding callouts.
8. The Appendix file needs to be in PDF format; title page should state "Appendix for + ms title" (author and affiliation list not necessary) and a brief Table of Contents with the page numbers for the listed items.
9. In the Data Availability section, please add resolvable links to the datasets. More information about the format of this section can be found here: https://link.springer.com/partners/embo-press/editorial-policies#Data%20availability%20statement.
10. Our data editors have indicated that the exact p values need to be provided in the legends of figures 6C, D.
11. In our standard textual plagiarism check, we noticed a couple of passages with high similarity to already published articles, which you can find in the attachment. Please edit these sentences accordingly.
12. Papers published in The EMBO Journal are accompanied online by a 'Synopsis' to enhance discoverability of the manuscript. It consists of A) a short (1-2 sentences) summary of the findings and their significance, B) 3-4 bullet points highlighting key results and C) a synopsis image that is 550x300 pixels large (width x height, jpeg or png format). You can either show a model or key data in the synopsis image. Please note that the image size is rather small and that text needs to be readable at the final size.

Please let me know if you have any questions regarding any of these points. You can use the link below to upload the revised files.

Thank you once more for giving us the chance to consider your manuscript for The EMBO Journal. I look forward to receiving the final version.

With best wishes,

Ieva

Ieva Gailite, PhD
Senior Scientific Editor
The EMBO Journal
Meyerhofstrasse 1
D-69117 Heidelberg
Tel: +4962218891309

i.gailite@embojournal.org

We realize that it is difficult to revise to a specific deadline. In the interest of protecting the conceptual advance provided by the work, we recommend a revision within 3 months (7th Sep 2026). Please discuss the revision progress ahead of this time with the editor if you require more time to complete the revisions.

Referee #1:

The authors have made substantiated improvements in the MS glycoproteomics and Cryo-EM data analyses to address all my comments. I recommend the publication of the revised manuscript.

The authors addressed the remaining editorial issues.

Editor accepted the manuscript for publication.

EMBO Press Author Checklist

Corresponding Author Name: Sheng Niu	
Journal Submitted to: The EMBO Journal	
Manuscript Number: EMBOJ-2025-121870R1	

USEFUL LINKS FOR COMPLETING THIS FORM
The EMBO Journal - Author Guidelines
EMBO Reports - Author Guidelines
Molecular Systems Biology - Author Guidelines
EMBO Molecular Medicine - Author Guidelines

Reporting Checklist for Life Science Articles (updated January
This checklist is adapted from Materials Design Analysis Reporting (MDAR) Checklist for Authors. MDAR establishes a minimum set of requirements in transparent reporting in the life sciences (see Statement of Task: 10.31222/osf.io/9sm4x). Please follow the journal's guidelines in preparing your
Please note that a copy of this checklist will be published alongside your article.

Abridged guidelines for figures
1. Data
The data shown in figures should satisfy the following conditions:
- the data were obtained and processed according to the field's best practice and are presented to reflect the results of the experiments in an accurate and unbiased manner.
- ideally, figure panels should include only measurements that are directly comparable to each other and obtained with the same assay.
- plots include clearly labeled error bars for independent experiments and sample sizes. Unless justified, error bars should not be shown for technical
- if n<5, the individual data points from each experiment should be plotted. Any statistical test employed should be justified.
- Source Data should be included to report the data underlying figures according to the guidelines set out in the authorship guidelines on Data

2. Captions
Each figure caption should contain the following information, for each panel where they are relevant:
- a specification of the experimental system investigated (eg cell line, species name).
- the assay(s) and method(s) used to carry out the reported observations and measurements.
- an explicit mention of the biological and chemical entity(ies) that are being measured.
- an explicit mention of the biological and chemical entity(ies) that are altered/varied/perturbed in a controlled manner.
- the exact sample size (n) for each experimental group/condition, given as a number, not a range;
- a description of the sample collection allowing the reader to understand whether the samples represent technical or biological replicates (including how many animals, litters, cultures, etc.).
- a statement of how many times the experiment shown was independently replicated in the laboratory.
- definitions of statistical methods and measures:
  - common tests, such as t-test (please specify whether paired vs. unpaired), simple χ2 tests, Wilcoxon and Mann-Whitney tests, can be unambiguously identified by name only, but more complex techniques should be described in the methods section;
  - are tests one-sided or two-sided?
  - are there adjustments for multiple comparisons?
  - exact statistical test results, e.g., P values = x but not P values < x;
  - definition of 'center values' as median or average;
  - definition of error bars as s.d. or s.e.m.

Please complete ALL of the questions below.
Select "Not Applicable" only when the requested information is not relevant for your study.

Materials

Newly Created Materials	Information included in the manuscript?	In which section is the information available? (Reagents and Tools Table, Materials and Methods, Figures, Data Availability Section)
New materials and reagents need to be available; do any restrictions apply?	Not Applicable	

Antibodies	Information included in the manuscript?	In which section is the information available? (Reagents and Tools Table, Materials and Methods, Figures, Data Availability Section)
For **antibodies** provide the following information:<br>- Commercial antibodies: RRID (if possible) or supplier name, catalogue number and or/clone number<br>- Non-commercial: RRID or citation	Yes	Reagents and Tools Table

DNA and RNA sequences	Information included in the manuscript?	In which section is the information available? (Reagents and Tools Table, Materials and Methods, Figures, Data Availability Section)
**Short novel DNA or RNA including primers, probes**: provide the sequences.	Not Applicable	

Cell materials	Information included in the manuscript?	In which section is the information available? (Reagents and Tools Table, Materials and Methods, Figures, Data Availability Section)
**Cell lines:** Provide species information, strain. Provide accession number in repository **OR** supplier name, catalog number, clone number, and/**OR** RRID.	Yes	Methods
**Primary cultures:** Provide species, strain, sex of origin, genetic modification status.	Not Applicable	
Report if the cell lines were recently **authenticated** (e.g., by STR profiling) and tested for mycoplasma contamination.	Not Applicable	

Experimental animals	Information included in the manuscript?	In which section is the information available? (Reagents and Tools Table, Materials and Methods, Figures, Data Availability Section)
**Laboratory animals or Model organisms:** Provide species, strain, sex, age, genetic modification status. Provide accession number in repository **OR** supplier name, catalog number, clone number, **OR** RRID.	Not Applicable	
**Animal observed in or captured from the field:** Provide species, sex, and age where possible.	Not Applicable	
Please detail **housing and husbandry conditions**.	Not Applicable	

Plants and microbes	Information included in the manuscript?	In which section is the information available? (Reagents and Tools Table, Materials and Methods, Figures, Data Availability Section)
**Plants:** provide species and strain, ecotype and cultivar where relevant, unique accession number if available, and source (including location for collected wild specimens).	Not Applicable	
**Microbes:** provide species and strain, unique accession number if available, and source.	Yes	Materials and Methods

Human research participants	Information included in the manuscript?	In which section is the information available? (Reagents and Tools Table, Materials and Methods, Figures, Data Availability Section)
If collected and within the bounds of privacy constraints report on age, sex and gender or ethnicity for all study participants.	Not Applicable	

Core facilities	Information included in the manuscript?	In which section is the information available? (Reagents and Tools Table, Materials and Methods, Figures, Data Availability Section)
If your work benefited from core facilities, was their service mentioned in the acknowledgments section?	Not Applicable	

Design

Study protocol	Information included in the manuscript?	In which section is the information available? (Reagents and Tools Table, Materials and Methods, Figures, Data Availability Section)
If study protocol has been **pre-registered, provide DOI in the manuscript**. For clinical trials, provide the trial registration number **OR** cite DOI.	Not Applicable	
Report the **clinical trial registration number** (at ClinicalTrials.gov or equivalent), where applicable.	Not Applicable	

Laboratory protocol	Information included in the manuscript?	In which section is the information available? (Reagents and Tools Table, Materials and Methods, Figures, Data Availability Section)
Provide DOI OR other citation details if **external detailed step-by-step protocols** are available.	Yes	Methods

Experimental study design and statistics	Information included in the manuscript?	In which section is the information available? (Reagents and Tools Table, Materials and Methods, Figures, Data Availability Section)
Include a statement about **sample size** estimate even if no statistical methods were used.	Yes	Methods
Were any steps taken to minimize the effects of subjective bias when allocating animals/samples to treatment (e.g. **randomization procedure**)? If yes, have they been described?	Yes	Methods
Include a statement about **blinding** even if no blinding was done.	Not Applicable	
Describe **inclusion/exclusion criteria** if samples or animals were excluded from the analysis. Were the criteria pre-established? If sample or data points were omitted from analysis, report if this was due to attrition or intentional exclusion and provide justification.	Not Applicable	
For every figure, are **statistical tests** justified as appropriate? Do the data meet the assumptions of the tests (e.g., normal distribution)? Describe any methods used to assess it. Is there an estimate of variation within each group of data? Is the variance similar between the groups that are being statistically compared?	Yes	Methods

Sample definition and in-laboratory replication	Information included in the manuscript?	In which section is the information available? (Reagents and Tools Table, Materials and Methods, Figures, Data Availability Section)
In the figure legends: state number of times the experiment was **replicated** in laboratory.	Yes	Figures
In the figure legends: define whether data describe **technical or biological replicates**.	Yes	Figures

Ethics

Ethics	Information included in the manuscript?	In which section is the information available? (Reagents and Tools Table, Materials and Methods, Figures, Data Availability Section)
Studies involving **human participants**: State details of **authority granting ethics approval** (IRB or equivalent committee(s), provide reference number for approval.	Not Applicable	
Studies involving **human participants**: Include a statement confirming that **informed consent** was obtained from all subjects and that the experiments conformed to the principles set out in the WMA Declaration of Helsinki and the Department of Health and Human Services Belmont Report.	Not Applicable	
Studies involving **human participants: For** publication of **patient photos**, include a statement confirming that consent to publish was obtained.	Not Applicable	
Studies involving experimental **animals**: State details of **authority granting ethics approval** (IRB or equivalent committee(s), provide reference number for approval. Include a statement of compliance with ethical regulations.	Not Applicable	
Studies involving **specimen and field samples:** State if relevant **permits** obtained, provide details of authority approving study; if none were required, explain why.	Not Applicable	

Dual Use Research of Concern (DURC)	Information included in the manuscript?	In which section is the information available? (Reagents and Tools Table, Materials and Methods, Figures, Data Availability Section)
Could your study fall under dual use research restrictions? Please check biosecurity documents and list of **select agents and toxins** (CDC): https://www.selectagents.gov/sat/list.htm	Not Applicable	
If you used a select agent, is the security level of the lab appropriate and reported in the manuscript?	Not Applicable	
If a study is subject to dual use research of concern regulations, is the name of the **authority granting approval and reference number** for the regulatory approval provided in the manuscript?	Not Applicable	

Reporting

The MDAR framework recommends adoption of discipline-specific guidelines, established and endorsed through community initiatives. Journals have their own policy about requiring specific guidelines and recommendations to complement MDAR.

Adherence to community standards	Information included in the manuscript?	In which section is the information available? (Reagents and Tools Table, Materials and Methods, Figures, Data Availability Section)
State if relevant guidelines or checklists (e.g., **ICMJE, MIBBI, ARRIVE, PRISMA**) have been followed or provided.	Not Applicable	
For **tumor marker prognostic studies,** we recommend that you follow the **REMARK** reporting guidelines (see link list at top right). See author guidelines, under 'Reporting Guidelines'. Please confirm you have followed these guidelines.	Not Applicable	
For **phase II and III randomized controlled trials**, please refer to the **CONSORT** flow diagram (see link list at top right) and submit the CONSORT checklist (see link list at top right) with your submission. See author guidelines, under 'Reporting Guidelines'. Please confirm you have submitted this list.	Not Applicable	

Data Availability

Data availability	Information included in the manuscript?	In which section is the information available? (Reagents and Tools Table, Materials and Methods, Figures, Data Availability Section)
Have **primary datasets** been deposited according to the journal's guidelines (see 'Data Deposition' section) and the respective accession numbers provided in the Data Availability Section?	Yes	Data Abailability Section
Were **human clinical and genomic datasets** deposited in a public access-controlled repository in accordance to ethical obligations to the patients and to the applicable consent agreement?	Not Applicable	
Are **computational models** that are central and integral to a study available without restrictions in a machine-readable form? Were the relevant accession numbers or links provided?	Not Applicable	
If publicly available data were reused, provide the respective **data citations in the reference list**.	Not Applicable	
